# Portfolio analysis of single-cell RNA-sequencing and transcriptomic data unravels immune cells and telomere-related biomarkers in sepsis

**DOI:** 10.3389/fimmu.2025.1638156

**Published:** 2025-10-30

**Authors:** Dan Chen, Xiyi Huang, Chun Wang, Cheng Zheng, Yunhao Liu

**Affiliations:** 1 Department of Environmental Health and Occupational Medicine, School of Public Health, Wuhan University of Science and Technology, Wuhan, China; 2 Department of Intensive Care Unit, The Central Hospital of Wuhan, Tongji Medical College, Huazhong University of Science and Technology, Wuhan, China; 3 Shantou University Medical College, Shantou, China

**Keywords:** sepsis, single-cell RNA sequencing, 101-machine learning, telomere, immune cells

## Abstract

**Background:**

Early diagnosis of sepsis is essential to reducing mortality. Immune cells and telomeres play important roles in sepsis, but their mechanisms were still unclear. This study aimed to explore the value of immune cells and telomere-related genes in sepsis.

**Methods:**

In this study, the transcriptomic data with sepsis and control samples were obtained from public database. Multiple methods including differential expression analysis, immune infiltration analysis, weighted gene co-expression network analysis (WGCNA), 101-machine learning algorithm combinations were used to identify biomarkers which related to the immune cells and telomere. Afterwards, a nomogram was constructed to assess the clinical predictive value of biomarkers. In addition, gene set enrichment analysis (GSEA), regulatory network construction and drug prediction analysis were adopted to demonstrate the role of biomarkers in sepsis. The key cells were also identified using a single-cell dataset. Finally, the expression of biomarkers was further validated in clinical samples by reverse transcription quantitative polymerase chain reaction (RT-qPCR).

**Results:**

This study obtained a total of 4 biomarkers (*MYO10*, *SULT1B1*, *MKI67*, and *CREB5*), and the analysis of nomogram showed that the biomarkers had good clinical predictive value to sepsis. The enrichment analysis results revealed that the four biomarkers were enriched in the ribosome pathway. Besides, a lncRNAs-miRNAs-biomarkers network was constructed for the four biomarkers. Finally, we obtained a candidate drug (MS-275) and a key cell (CD16+ and CD14+ monocytes) respectively based on drug prediction and cell identification analysis. In addition, we found that the expression levels of *CREB5* and *SULT1B1* had significant changes during the process of key cell differentiation. The RT-qPCR results showed biomarkers were upregulated in the sepsis group, consistent with the bioinformatics analysis results.

**Conclusion:**

This study identified 4 biomarkers, namely *MYO10*, *SULT1B1*, *MKI67*, and *CREB5* and explored the pathogenesis of sepsis, providing new insights for potential treatment strategies by integrating transcriptomic data and single-cell analysis.

## 1 Introduction

Sepsis is a life-threatening organ dysfunction caused by a dysregulated host response to infection. It is characterized by systemic immune activation, metabolic abnormalities, and multi-organ dysfunction (1). It causes approximately 11 million deaths globally each year (2). Although the incidence and mortality rates have decreased in recent years, sepsis remains a significant health burden (3). Due to its nonspecific clinical presentation and the limitations of current diagnostic methods, early diagnosis remains challenging, making the discovery of novel biomarkers a key focus of research (4).

The pathogenesis involves complex disorders of immune regulation. Early intervention targeting excessive inflammation has shown poor outcomes. Recent studies have revealed that sepsis is often accompanied by adaptive immune suppression, with functional exhaustion of immune cells linked to adverse prognoses (5). This process may be associated with telomere dysfunction: telomere shortening can trigger cellular senescence and genomic instability, while excessive activation of immune cells in sepsis may accelerate telomere attrition. Clinical studies have demonstrated an association between peripheral blood leukocyte telomere length and survival rates in patients (6). Given the limited sensitivity of traditional biomarkers, the functional state of immune cells and telomere length may offer more precise assessments of disease severity. This study aims to identify gene markers associated with telomeres and immune cells through integrated bioinformatics analysis, in order to elucidate their roles in sepsis progression and uncover novel therapeutic targets.

Single-cell RNA sequencing (scRNA-seq) is a revolutionary technology that, compared to traditional bulk sequencing methods, enables multi-omics analysis (genomic, transcriptomic, epigenomic) at the single-cell level to reveal intercellular heterogeneity (7, 8). Its advantages include the identification of rare cell subsets, decoding of cell-cell communication, and tracking of dynamic changes, providing high-precision data for disease mechanism research (9–11). Machine learning (ML), which extracts patterns from complex data via algorithms, has been widely applied in biomedicine, but it faces challenges in interpretability and multimodal data integration (12, 13). To address these limitations, researchers are continuously exploring novel ML approaches. For example, “101-machine learning” is an emerging technique designed to enhance ML performance and applicability through efficient data processing and model training. In this study, we utilized ML to optimize feature selection and model training, thereby improving the efficiency and accuracy of target screening (14).

This study was based on transcriptomic data from public databases, and 101-machine learning algorithms were employed to screen for sepsis-related biomarkers. Through in-depth analysis of their clinical diagnostic value, enriched functions, involved biological statistics, and interactions with the immune microenvironment, we explored the functions and regulatory mechanisms of these genes across different biological levels. Finally, single-cell analysis techniques were used to elucidate the cell-type-specific expression and distribution patterns of these genes in sepsis. These findings lay a solid foundation for developing novel therapeutic strategies for sepsis and are expected to advance innovative treatments and personalized medicine in this field.

## 2 Materials and methods

### 2.1 Data collection

**Figure 1:**
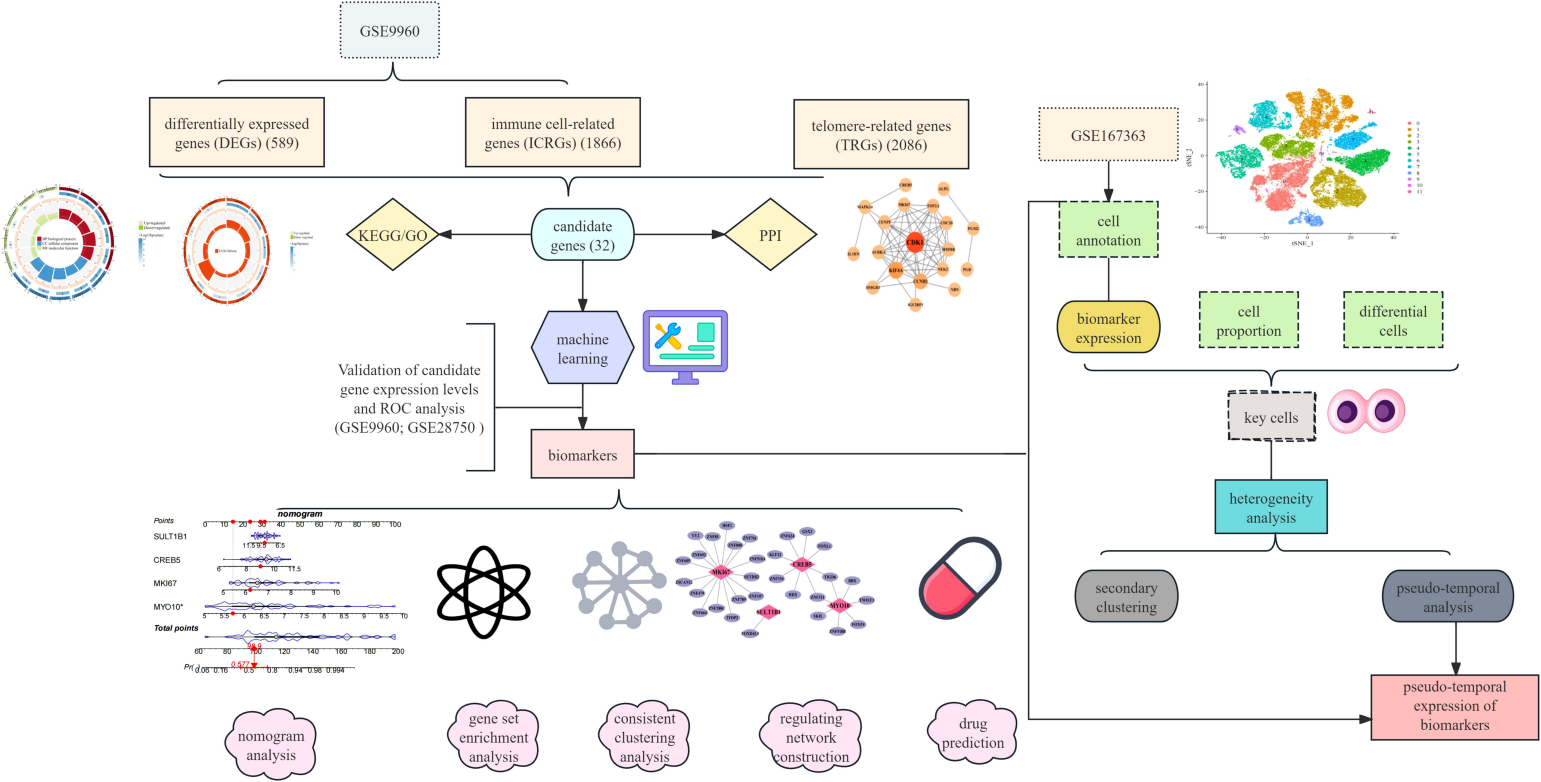
This study analyzes the flowchart.

Three microarray datasets in this study were obtained from the Gene Expression Omnibus (GEO) database using the “GEOquery” package (version 4.2.1) (15). Among them, GSE9960 and GSE28750 both used the GPL570 sequencing platform (16). GSE9960 contained 70 peripheral blood mononuclear cell samples, including 54 sepsis samples as the case samples and 16 normal controls as the control samples. GSE28750 contained 30 blood samples, of which 10 sepsis samples were the case samples and the remaining were normal controls. The scRNA-seq dataset based on the GPL24676 sequencing platform, GSE167363 contained 12 peripheral blood mononuclear cells samples, including 10 sepsis case samples and 2 normal control samples (17). The 2086 telomere-related genes (TRGs) were extracted from TelNet database (http://www.cancertelsys.org/telnet) (18). The analysis process of this study is shown in **Figure 1**.

### 2.2 Differential expression analysis

The PCA in GSE9960 dataset was performed through the “scatterplot3d” package (v 0.3-42) (19). The differentially expressed genes (DEGs) between sepsis and normal samples in GSE9960 dataset were identified using the “limma” package (v 3.54.1) (20). The screening criteria were *P* < 0.05 & |log_2_Fold Change (FC)| > 0.5. The top 10 genes up-regulated and down-regulated in the sepsis samples based on the |log_2_FC| values were displayed by volcano plot and heat plot which made use of “ggplot2” package (v 3.4.1) (21) and “ComplexHeatmap” package (v 2.14.0) (22), respectively.

### 2.3 Immune infiltration analysis and weighted gene co-expression network analysis

In our study, the relative distribution ratios of 22 immune cells in each sample in GSE9960 were analyzed by using the cell identification by estimating relative subsets of RNA transcripts (CIBERSORT) algorithm (v 0.1.0) (23), and immune cells with a proportion of 0 over 30% samples were removed in the subsequent analysis. The differences of immune cells infiltration between the case and control group were completed through the Wilcoxon rank sum test (*P* < 0.05).Module genes highly correlated with immune cells were selected using the “WGCNA” package (v 1.71) (24). Sample cluster analysis used the hierarchical clustering (HCLUST) function to identify and eliminate outliers. Then, we set R^2^ = 0.85 to screen for soft thresholds (β). Topology overlap and adjacency matrices were established based on the gene expression data. Afterwards, the minimum gene count per module was set to 100 with the altitude of models being set to 0.4, and the modules were merged according to the standards of the dynamic tree cutting algorithm. Then, differential immune cell scores were used as the phenotype to calculate the correlations between key module and phenotype. Finally, the modules with the best comprehensive score from the modules which significantly correlated with phenotype were obtained as the final module. The genes in final modules significantly correlated with phenotype were selected as the immune cell-related genes (ICRGs) (*P* < 0.05).

### 2.4 Enrichment analysis of candidate genes and protein-protein interaction network

The candidate genes in our study were obtained by overlapping the ICRGs, DEGs and TRGs, which made use of the “ggvenn” package (v 1.7.3) (23).

The functions of candidate genes were explored by the kyoto encyclopedia of genes and genomes (KEGG) and gene ontology (GO) analyses with the “clusterProfiler” package (v 4.2.2) (25) (*P* < 0.05). Furthermore, the protein level interactions of candidate genes were analyzed by PPI network using data from the search tool for the recurring instances of neighboring genes (STRING) database (confidence > 0.4) (26).

### 2.5 Construction of 101-machine learning models and identification of biomarkers

In order to identify feature genes associated with sepsis, the leave-one-out cross-validation (LOOCV) framework was utilized in the GSE9960 and GSE28750 datasets that integrated ten different machine learning algorithms into 101 algorithm combinations. The input data for machine learning algorithms was the candidate genes, and the ten machine learning algorithms comprised RSF and Enet by the “glmnet” package (v 4.1.4) (27), least absolute shrinkage and selection operator (LASSO) by the “glmnet” package (v 4.1.4) (27), Ridge by the “glmnet” package (v 4.1.4) (27), stepwise Cox by the “caret” package (v 6.0-93) (28), xBoost by the “xgboost” package (v 1.7.3.1) (29), plsRcox by the (v 3.2.2), SuperPC by the “superpc” package (v 1.12) (30), generalized linear mixed model (GBRM) by the “gbm” package (v 2.1.8.1) (31), and survival-SVM by the “e1071” package (v 1.7-13) (32). Subsequently, the receiver operating characteristic (ROC) curve drawn using the “pROC” package (v 1.18.0) (33) was used to validate the predictive performance of the 101-machine learning algorithm combinations when the minimum and the maximum number of genes in the model was from 2 to 10. The combination model with the highest area under the curve (AUC) value of ROC curve was considered as the optimal model. The genes of the optimal model were considered as the candidate biomarkers for subsequent analysis.

In this study, ROC curve and expression level analysis were also performed on the GSE9960 and GSE28750 datasets to identify biomarkers with sepsis (AUC > 0.7). The expression level analysis was used by Wilcoxon test, which required that the expression trends of genes in two datasets were consistent (*P* < 0.05).

### 2.6 Gene correlation analysis and distribution analysis in organs

In this study, the genotype-tissue expression (GTEx) database was employed mainly for querying the expression levels of biomarkers in 28 normal tissues and organs of the human body. Besides, correlations among biomarkers were carried out using Spearman’s correlation test with the “psych” package (v 2.2.9) (34) in the GSE9960 datasets.

### 2.7 Nomogram analysis

To investigate the specific role of biomarkers in the diagnosis of sepsis, the nomogram based on biomarkers was constructed using the “regplot” package (v 1.1) (35) in the GSE9960 datasets. The accuracy of prediction with nomogram was determined by the calibration curve, which was drawn by the “rms” package (v 6.5.0) (36). Furthermore, the predicting value of the nomogram was also appraised by ROC curve.

### 2.8 Gene set enrichment analysis

In order to explore the biological pathways and functions with biomarkers, gsea was executed by “clusterProfiler” package in the GSE9960 datasets (37). Primarily, the Spearman correlation coefficients between biomarkers and all genes were calculated in the disease samples of the GSE9960 dataset. The GSEA of biomarkers was conducted according to the sequencing list of genes which corresponded to biomarkers (*P*.adjust < 0.05, |normalized enrichment score (NES) |> 1). In this step of analysis, the gene set from the “org.Hs.eg.dbR” package (v 3.1.0) (38) was employed as the background gene set.

### 2.9 Consistent clustering analysis

In order to cluster the sepsis samples into different clusters in the GSE9960 dataset, the k-means algorithm with 1000 iterations was executed by “ConsensusClusterPlus” package. In order to determine the expression of biomarkers and the differences in immune cell infiltration different subtypes on sepsis, the expression of biomarkers and immune infiltration analysis was performed between the different clusters according to the previously referenced method (*P* < 0.05).

### 2.10 Regulating network construction and drug prediction

LncRNAs can control the expression of mRNAs by binding to shared miRNAs. So, in this study, the miRTarBase v9.0 database and TarBase v9.0 database were manipulated based on the NetworkAnalyst platform to forecast the miRNAs which could target biomarkers. Then, the final miRNAs were obtained by crossing over the miRNAs from the two databases. Finally, the miRNet database was used to predict the lncRNAs which could target the final miRNAs, and the lncRNA-miRNA-mRNA network was visualized by Cytoscape software (v 3.9.1) (39).

In addition, this study also employed ChEA3 database to forecast the transcription factors (TFs) which could target biomarkers. The biological function (*P* < 0.05) and the distribution in the tissues of TFs also were obtained from the ChEA3 database.

To search for potential therapeutic drugs related to biomarkers for sepsis, the “enrichR” package (v 3.2) (39) was used based on the Drug Signatures Database (DSigDB) database to predict genes-drug interactions (*P* < 0.05). Afterwards, in order to evaluate the binding ability between drugs and biomarkers, the drugs which had the highest significance with biomarkers were selected to perform molecular docking. The three-dimensional structure with proteins of biomarkers was retrieved from the Protein Data Bank (PDB) database (https://www.rcsb.org/) and the three-dimensional structure with molecular ligands of key active ingredients was retrieved from the PubChem database. Finally, molecular ligands and proteins were docked using online website.

### 2.11 Single-cell RNA-sequencing analysis

The scRNA-seq analysis was conducted using “Seurat” package (v 5.0.1) (40). To ensure the accuracy of single-cell data, all samples in the GSE167363 dataset were dealt with the PercentageEigenSet function of the “Seurat” package. The data processing conditions were as follows: the number of genes in cells ranges from 300 to 10000, the expression level of genes in cells between 300-2000, the genes expressing in at least three cells, and the proportion of mitochondrial genes less than 15% in cells. Then, in light of the GSE167363 dataset, data were normalized by the “NormalizeData” function in the “Seurat” package (v 5.0.1), and highly variable genes (HVGs) were selected by the “FindVariableFeatures” function. Next, the “ScaleData” function in the “Seurat” package (v 5.0.1) was applied to scale data before principal components analysis (PCA). Subsequently, the “JackStraw” function within the “Seurat” package (v 5.0.1) was applied to execute PCA on HVGs. The “ElbowPlot” function within the “Seurat” package (v 5.0.1) was thereafter applied to draw a scree plot of the top 30 principal components (PCs), aiming to identify PCs that notably contributed to variation for subsequent analysis (*P* < 0.05). Afterward, cell cluster analysis was conducted on cells after dimensionality reduction utilizing “FindNeighbors” and “FindClusters” functions (resolution = 0.1, dimension = 30). After that, the cells were clustered by the uniform tSNE clustering method (41). Marker genes for cell annotation in this study were obtained from the literature (15).

To determine the key cell types of sepsis in the GSE167363 dataset, the proportion of different types of cells in different samples, the differential infiltration of cells, and expression of biomarkers in different cell types were all considered. So, the cells that satisfied the following criteria simultaneously as key cells: (1). high proportion of cells in the sample; (2). cells with different infiltration ratios between the disease and control; (3). cells with different expression of biomarkers (*P* < 0.05). Subsequently, the secondary clustering analysis on key cells was performed which referred to the previous method, and the pseudo time analysis was proceeded by the “Monocle” package (v 2.30.0) (42) to study the developmental trajectory and differentiation directions of key cell types. Eventually, the expression of biomarkers during cell differentiation was observed.

### 2.12 Reverse transcription quantitative polymerase chain reaction

The assessment of biomarkers expression was conducted on clinical tissue samples using RT-qPCR. A total of 5 pairs of blood samples were obtained from Wuhan Central Hospital Affiliated to Huazhong University of Science and Technology, including 5 sepsis and 5 control. All participants needed to sign and fill the informed consent form, and the ethical approval agency was Ethics Committee of The Central Hospital of Wuhan (No. WHZXKYL2024-164). Firstly, the total RNA of 5 pairs of tissue samples was derived by TRizol reagent (Ambion, U.S.A). The RNA concentrations were computered by NanoPhotometer N50. Secondly, mRNA was reversely transcribed into complementary DNA (cDNA) utilizing SureScript-First-strand-cDNA-synthesis-CREB5B test kit (Servicebio, Wuhan, China). Finally, the RT-qPCR was conducted. The expression levels of biomarkers between sepsis and control samples were calculated by 2^-ΔΔCt^. The internal reference gene was glyceraldehyde-3-phosphate dehydrogenase (GAPDH), which was employed to normalize the results. The results were calculated by GraphPad Prism 5. Detailed information of primers and machine testing conditions was listed in **Supplementary Table S1**.

### 2.13 Statistical analysis

Bioinformatics analyses were performed utilizing the R programming language (v 4.2.2). Wilcoxon rank sum test was used to compare the differences between two groups. *P* < 0.05 was considered statistically significant.

## 3 Results

### 3.1 Identification of candidate genes

**Figure 2:**
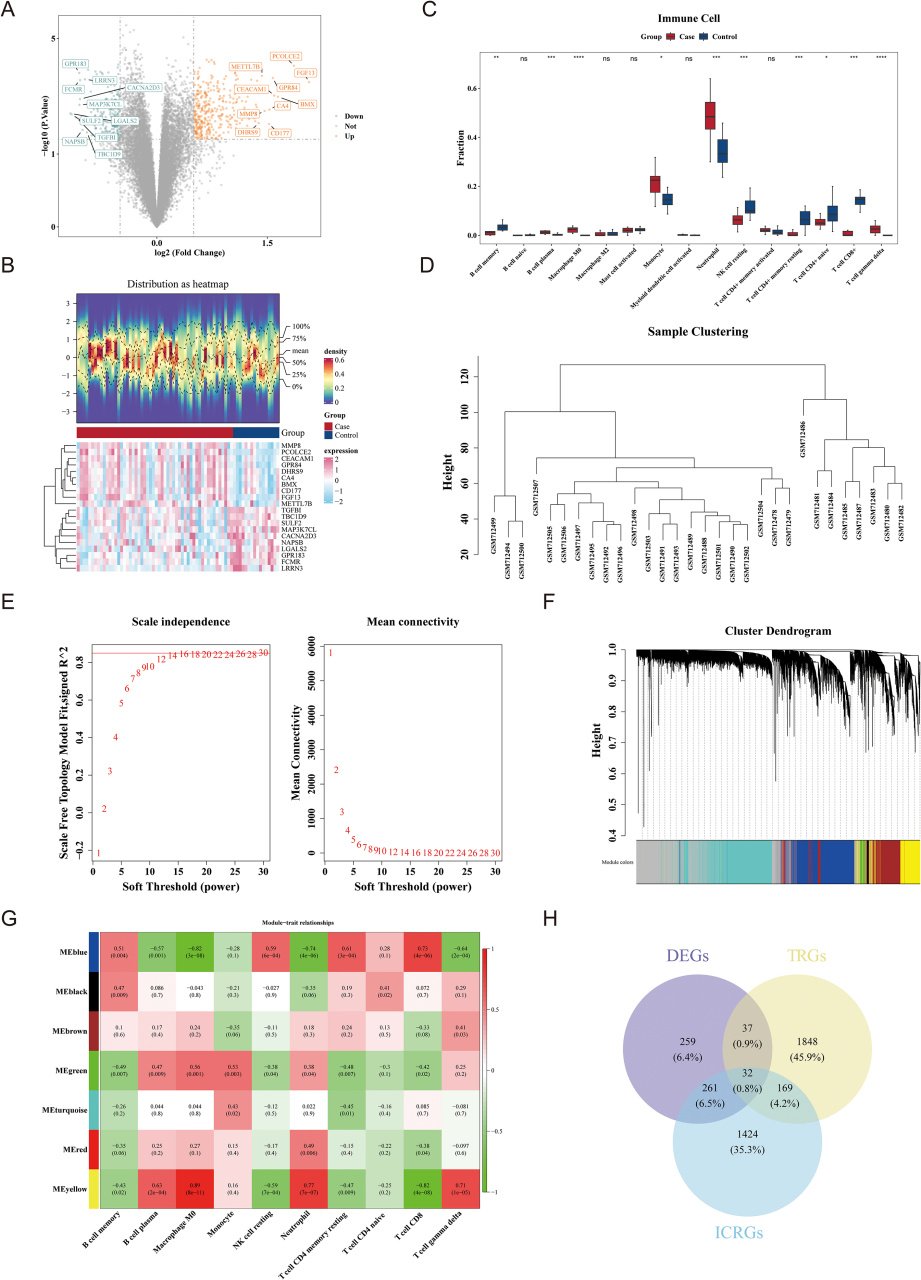
Identification of candidate genes in the GSE9960 dataset. **(A)** Volcano plot showing DEGs between case and control samples in GSE9960 (|logFC| > 1, adjusted *P* < 0.05). ns means not significant; *means P < 0.05; ** means P < 0.01; *** means P < 0.001; **** means P < 0.0001. Upregulated and downregulated genes are marked in orange and blue, respectively. **(B)** Heatmap showing expression profiles of DEGs across samples. **(C)** Boxplot comparing immune cell infiltration levels between groups. **(D)** Sample clustering dendrogram confirming the absence of outliers for WGCNA. **(E)** Scale-free topology model fit and mean connectivity across soft-thresholding powers. **(F)** Gene clustering dendrogram with color-coded modules identified by WGCNA. **(G)** Heatmap illustrating module–trait correlations between WGCNA modules and immune cell scores. **(H)** Venn diagram showing intersecting genes among DEGs, ICRGs, and TRGs.

The results of PCA showed that the case and control samples in the GSE9960 dataset were relatively separated, but there were no outlier samples, indicating good stability of the group samples (**Supplementary Figure S1**). So, the DEGs were obtained between all case and control samples in this dataset. A total of 589 DEGs were identified, of which 342 were up-regulated and 247 were down-regulated in the case group (**Figures 2A, B**). In the immune infiltration analysis, 15 types of immune cells were remained after removing cells that did not conform to the criteria (**Supplementary Figure S2**). Then, a significant difference was observed in the infiltration of 10 immune cells between the case and the control group, including memory B cell, plasma B cell, Macrophage M0, Monocyte, Neutrophil, resting natural killer (NK) cell, CD+ resting memory T-cell, naive CD4+ T cell naive, CD8+ T cell CD8+, and gammadelta T cell. The 10 immune cells were included in subsequent analysis (**Figure 2C**).

In WGCNA, no outlier samples were detected and subsequent analysis was based on all samples (**Figure 2D**). Afterwards, the soft threshold was 30 when R^2^ = 0.85, and a total of 7 co-expression modules were obtained (**Figures 2E, F**). The correlation analysis indicated that the MEyellow module exhibited the strongest correlation with the immune cell score, and 1,886 ICRGs were identified (**Figure 2G**). Ultimately, 32 candidate genes were obtained by taking the intersection of ICRGs, DEGs, and TRGs (**Figure 2H**).

### 3.2 The analysis of enrichment analysis and PPI network

**Figure 3:**
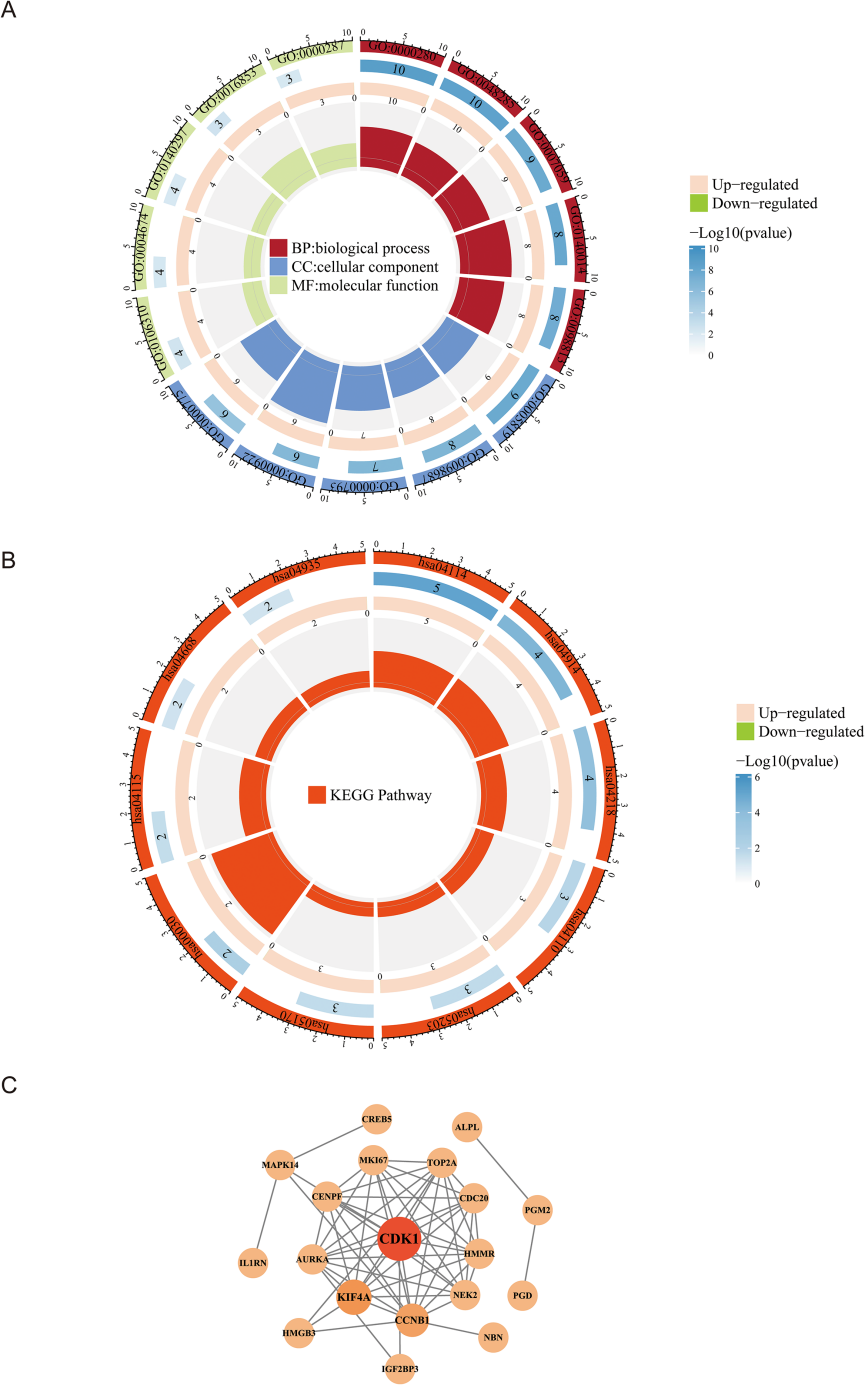
Functional enrichment and interaction analysis of candidate genes. **(A)** GO enrichment analysis of 32 candidate genes visualized in a circular layout. **(B)** KEGG pathway enrichment visualized in a circular plot. **(C)** PPI network constructed from the candidate genes, with CDK1 identified as the central hub.

The GO enrichment analysis of the candidate genes revealed that there were 440 significant functions, including nuclear division, organelle fission, spindle, chromosomal region, protein serine kinase activity, protein serine/threonine kinase activity, etc. (**Figure 3A**) (**Supplementary Table S2**). Meanwhile, a total of 15 significant functions were enriched in the KEGG analysis which included oocyte meiosis, progesterone-mediated maturation of oocytes, cellularsenescence, cell cycle, vral carcinogenesis, etc. (**Figure 3B**) (**Supplementary Table S3**). The PPI network was established based on the candidate genes, 19 genes had interactive network relationship. It was worth noting that CDK1 exhibited interactions with the most genes (**Figure 3C**).

### 3.3 Identification of biomarkers

**Figure 4:**
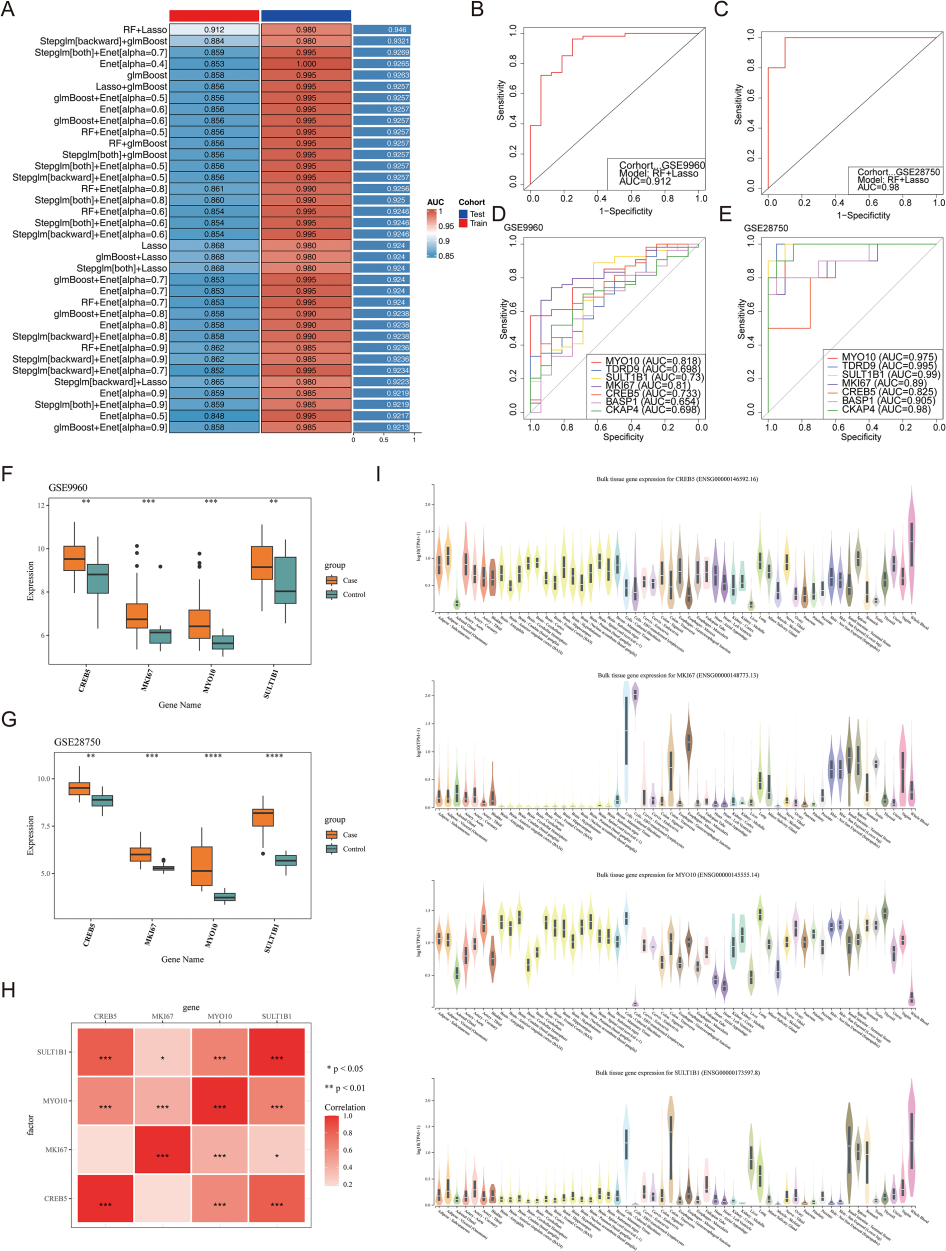
Identification and validation of diagnostic biomarkers for sepsis. **(A)** Heatmap of AUC values from multiple machine learning models, including RF, LASSO, and glmBoost. **(B)** ROC curve evaluating model performance in the GSE9960 dataset. **(C)** ROC curve evaluating model performance in the GSE28750 dataset. **(D)** ROC curves for *MYO10*, *MKI67*, *CREB5*, and *SULT1B1* in GSE9960. **(E)** ROC curves for the same genes in GSE28750. **(F)** Expression levels of four genes in sepsis and control groups in GSE9960. ** means P < 0.01; *** means P < 0.001. **(G)** Expression levels of four genes in sepsis and control groups in GSE28750. ** means P < 0.01; *** means P < 0.001; ****means P < 0.0001. **(H)** Correlation heatmap showing pairwise associations among the four candidate genes. * means P < 0.05; *** means P < 0.001. **(I)** Violin plots showing tissue-specific expression of the four genes based on GTEx data.

A total of 36 models were obtained when machine learning combination models were selected, among which the “RF+Lasso” model was considered the optimal model due to its highest value of AUC (**Figures 4A–C**). At the same time, the 7 genes such as *MYO10*, TDRD9, *SULT1B1*, *MKI67*, *CREB5*, BASP1, and CKAP4 in the “RF+Lasso” model were placed in subsequent analysis. The ROC curve analysis of the7 genes indicated that the AUC values of *MYO10*, *SULT1B1*, *MKI67*, and *CREB5* were greater than 0.7 in the two datasets (**Figures 4D, E**). These 4 genes were differentially expressed between groups in both datasets, and all were up-regulated in the case group (**Figures 4F, G**). Therefore, *MYO10*, *SULT1B1*, *MKI67*, and *CREB5* were selected as the biomarkers for this study. At that time, all 4 biomarkers showed a positive correlation, and the highest positive correlation occurred between *SULT1B1* and *CREB5* (cor = 0.823182574, p = 2.24×10^-18^) (**Figure 4H**). Otherwise, the GTEx database was occupied to observe the distribution of biomarkers in human tissues and organs (**Figure 4I**). The results indicated that *CREB5* and *SULT1B1* had the highest expression level in whole blood tissue, and *MKI67* had the highest expression level in cell-Cultured fibroblasts. Unusually, the levels of expression with *MYO10* in all tissues were similar.

### 3.4 Construction of nomogram

**Figure 5:**
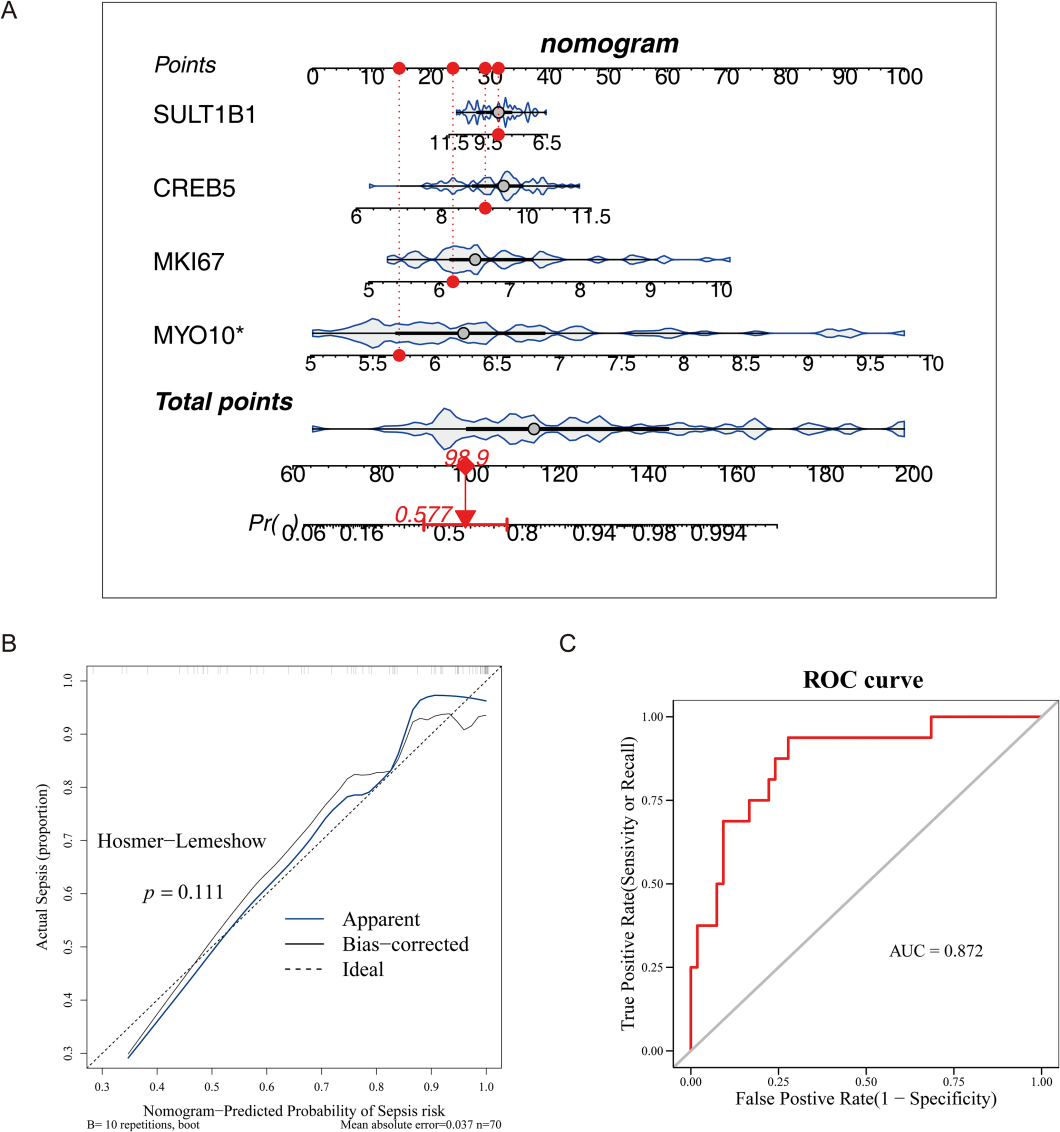
Diagnostic nomogram construction and validation for sepsis. **(A)** Nomogram constructed using four candidate genes to predict sepsis probability. **(B)** Calibration curve comparing predicted and observed outcomes. **(C)** ROC curve assessing the diagnostic performance of the nomogram.

The nomogram consisted of “points” and “total points”, with the points representing the points of biomarkers and the latter representing the total points of all biomarkers. The sum of gene points indicated that the higher the total score, the higher likelihood of sepsis in this sample. As shown in the **Figure 5A**, the point of *MYO10* was the highest indicated that *MYO10* had the greatest impact on the predictive value of sepsis and the result was significant (*P* < 0.05). In addition, the P-value of the calibration curve was 0.111, indicating the accuracy of prediction with the nomogram was quite well (**Figure 5B**). Similarly, the AUC in ROC of the model was 0.872, also indicating good prediction performance of the model (**Figure 5C**).

### 3.5 The enrichment pathway of the biomarkers

**Figure 6:**
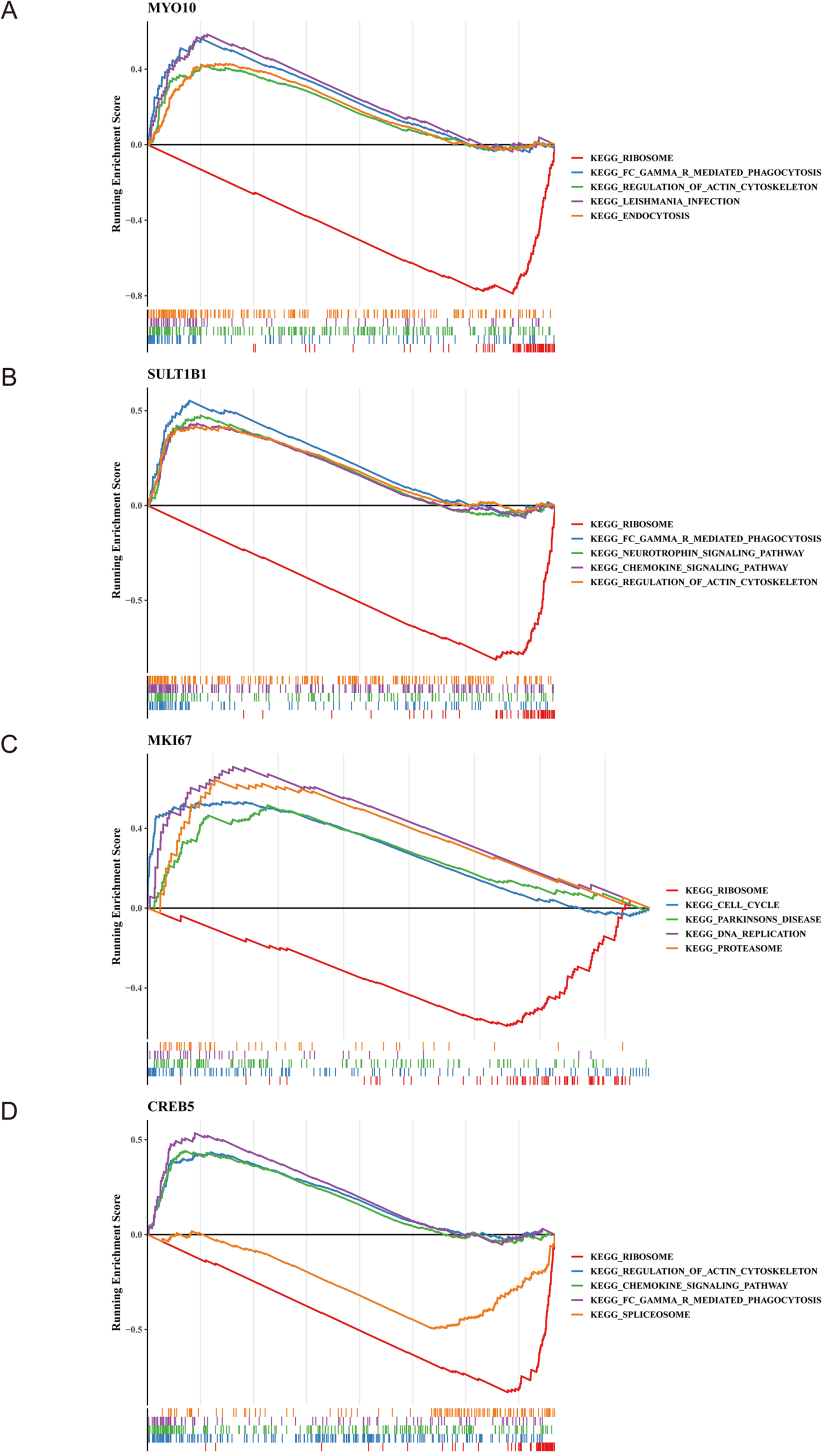
KEGG pathway enrichment analysis for *MYO10*, *SULT1B1*, *MKI67*, and *CREB5*. Pathway enrichment analysis based on GSEA for *MYO10***(A)**, *SULT1B1***(B)**, *MKI67***(C)**, and *CREB5***(D)**.

The GSEA revealed the enrichment pathway of the biomarkers. The results showed that the four biomarkers were all enriched in ribosome, and 3 biomarkers (*MYO10*, *SULT1B1*, *CREB5*) were enriched in Fc gamma R-mediated phagocytosis. The common enrichment pathway of *CREB5* and *SULT1B1* was the chemokine signaling pathway. The co-enrichment pathway of *CREB5* and *MYO10* was the regulation of actin cytoskeleton (**Figures 6A–D**).

### 3.6 Consensus clustering analysis of biomarkers

**Figure 7:**
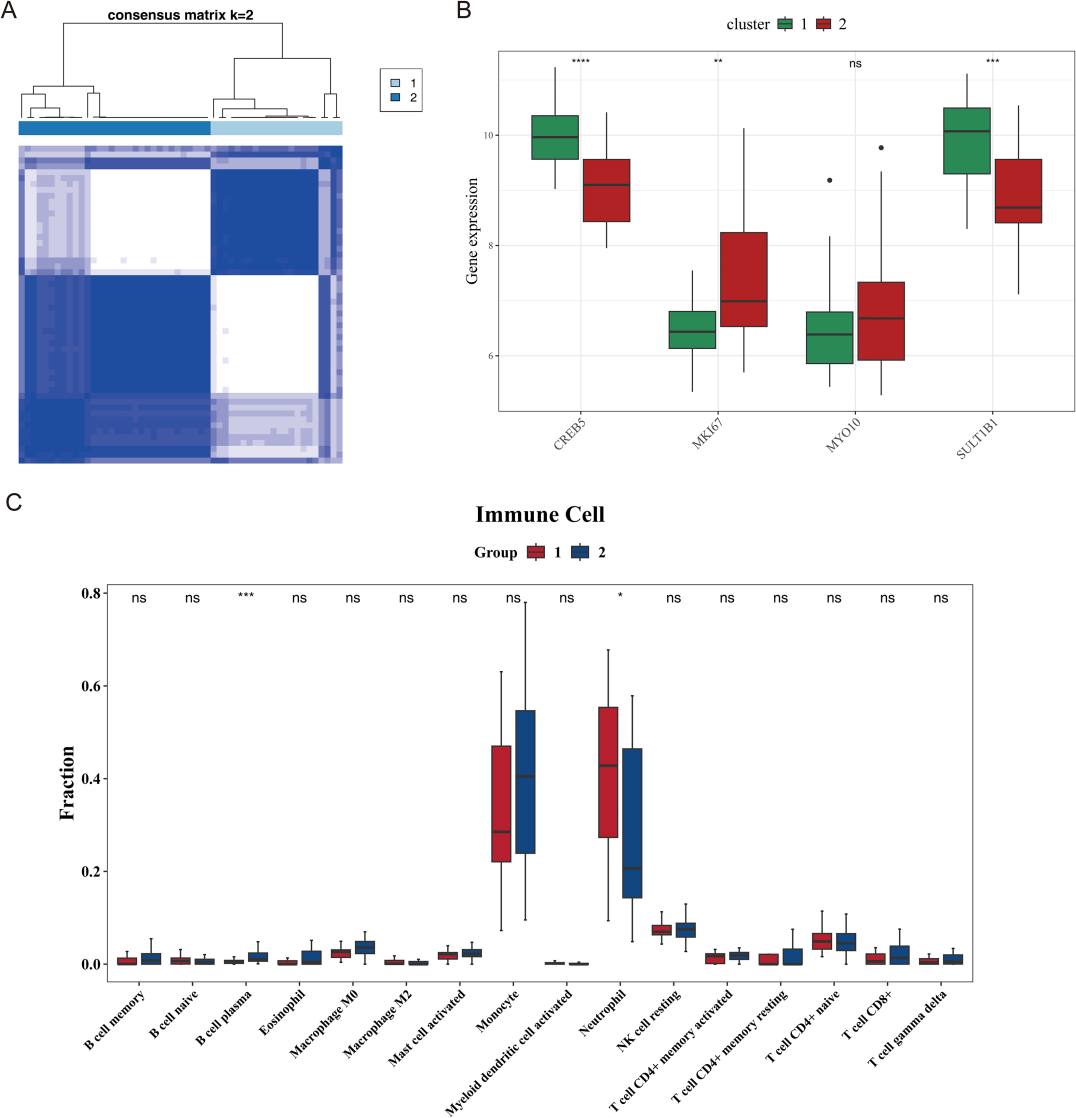
Consensus clustering of sepsis subtypes and associated immune characteristics. **(A)** Consensus matrix heatmap identifying two stable clusters (k = 2) among sepsis samples. **(B)** Expression levels of *CREB5*, *MKI67*, *MYO10*, and *SULT1B1* in the two clusters. ns means not significant; ** means P < 0.01; **** means P < 0.0001. **(C)** Comparison of immune cell infiltration levels between the two clusters. ns means not significant; * means P < 0.05; *** means P < 0.001.

As shown in the **Figure 7A**, the best cluster stability was achieved when the number of clusters (K) equaled 2. Consequently, the sepsis samples in the GSE28750 dataset were divided into two subgroups, including cluster1 and cluster2. To obtain the expression trends of biomarkers between cluster1 and cluster2, the Wilcoxon test was used. The results indicated that the expression of *MKI67*, *SULT1B1* and *CREB5* were significantly different between two clusters. Specifically, *MKI67* was up-regulated in cluster2, while *CREB5* and *SULT1B1* were up-regulated in cluster1 (**Figure 7B**). To investigate the different proportion of immune cell infiltration between two clusters, 22 immune cells were included based on the CIBERSORT algorithm (**Supplementary Figure S3**). But only 16 immune cells were included in differential analysis, and only two cells stypes (B cell plasma b and Neutrophil) showed significant differences between clusters (*P* < 0.05) (**Figure 7C**).

### 3.7 Identification of non-coding RNA and TFs

**Figure 8:**
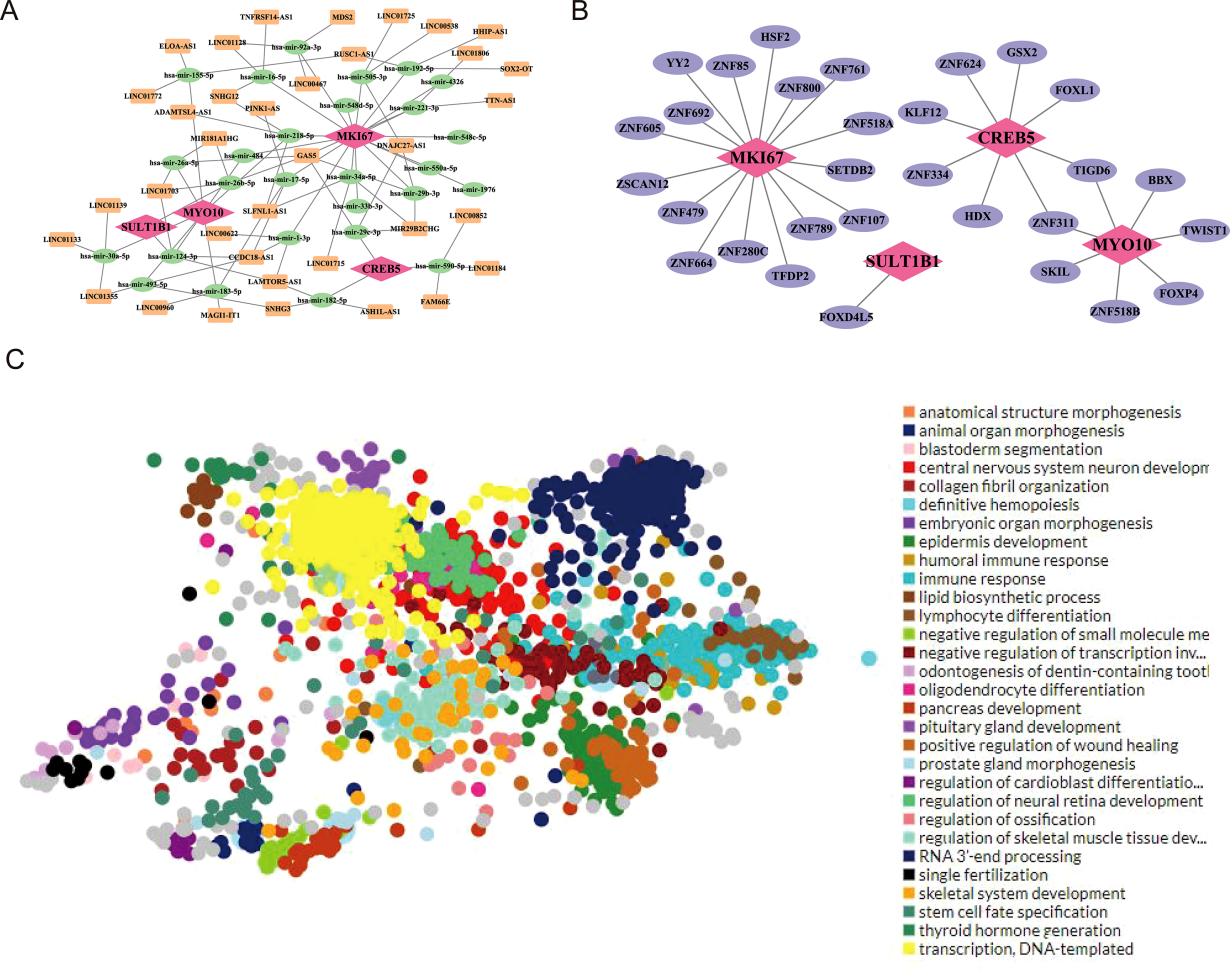
Regulatory network analysis of candidate genes. **(A)** lncRNA–miRNA–mRNA regulatory network constructed for *MYO10*, *MKI67*, *CREB5*, and *SULT1B1*. **(B)** Transcription factor regulatory network associated with the four candidate genes. **(C)** GO functional annotation of regulatory modules.

By using the NetworkAnalyst platform, a total of 30 miRNAs were identified for four biomarkers. Among them, 3 miRNAs were predicted with *CREB5*, 17 miRNAs were predicted with *MKI67*, 8 miRNAs were predicted with *MYO10*, and 2 miRNAs were predicted with *SULT1B1*. Specifically, the miRNA which targeted *MYO10* and *SULT1B1* was hsa-mir-124-3p. while that targeted *MYO10* and *MKI67* was hsa-mir-218-5p. Next, 1,055 lncRNA-miRNA interactions were obtained by the miRNet database, and the top 3 lncRNAs related to each miRNA such as which included *MKI67*-hsa-mir-484-SNHG12, *MKI67*-hsa-mir-218-5p-SLFNL1-AS, and *MYO10*-hsa-mir-218-5p-ADAMTSL4-AS1 were selected to construct a network diagram. The lncRNAs-miRNAs-biomarkers network were exhibited in the **Figure 8A**, Besides, a total of 703 TFs were identified for four biomarkers. We selected the top 30 TFs which had higher correlation with the biomarkers for the TF-biomarkers network. The network was visualized by Cytoscape software. The number of TFs predicted with *MKI67* was the largest (**Figure 8B**). The enrich function of TF mainly included anatomical structure morphogenesis, animal organ morphogenesis, and blastoderm segmentation. Finally, we also observed the expression of TFs in the tissue distribution, and the results displayed that the expression of TFs were observed in nerve, colon, uterus, skin, and blood vessel (**Figure 8C**).

### 3.8 Drug prediction and molecular docking

**Figure 9:**
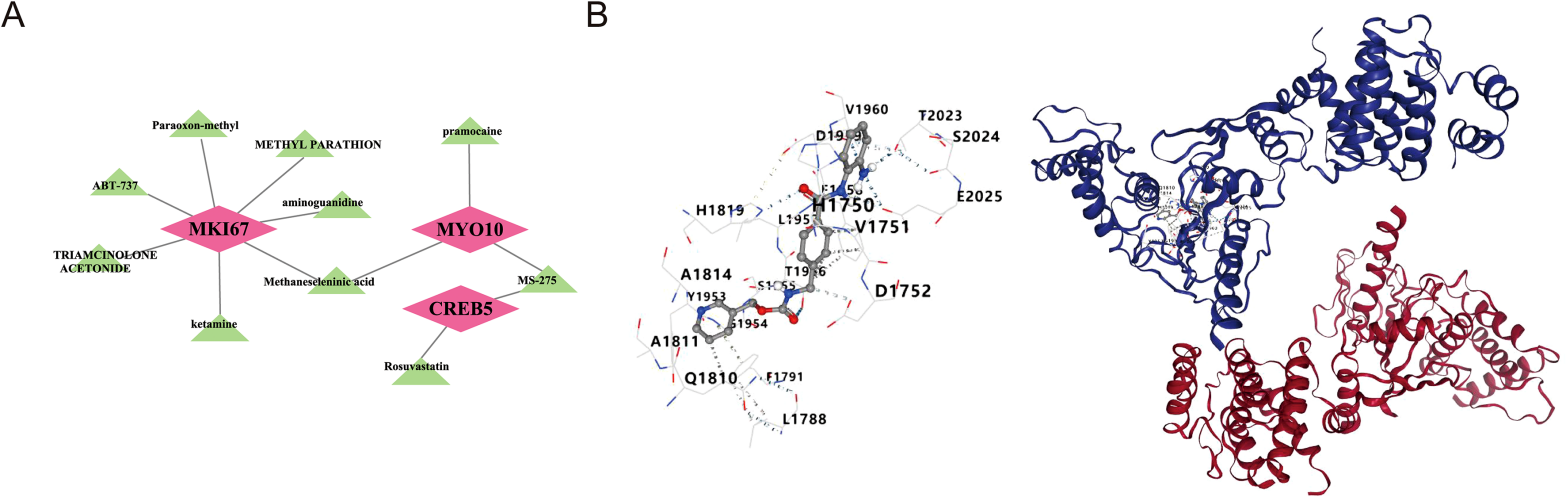
Drug–gene interaction network and molecular docking analysis. **(A)** Drug–gene interaction network for *MYO10*, *MKI67*, and *CREB5*. **(B)** Molecular docking model of a representative small molecule and its target protein.

A total of 80 drugs were chosen in the DSigDB database. Subsequently, we selected the top 10 drugs with higher significance to construct drug-biomarkers network by Cytoscape software. In the drug-biomarkers network, it could be observed that Methaneseleninie acid was targeted to the *MYO10* and *MKI67* while MS-275 was targeted to the *MYO10* and *CREB5* (**Figure 9A**). Due to its highest saliency with biomarkers and three-dimensional structure, MS-275 was selected to perform molecular docking analysis. *MYO10* was selected as the gene also because of the three-dimensional structure for molecular docking analysis. It is generally believed that |total score| > 7.0 kcal/mol indicated a stronger binding activity. In our study, the total score was -9.4 kcal/mol, indicating that the biomarkers had preferable binding activity with the target protein (**Table 1**) (**Figure 9B**).

**Table 1:** Title.

Biomarkers-drug	Total score	Center
*MYO10*-(MS-275)	-9.4	12,1,-62

### 3.9 Identification of key cells

**Figure 10:**
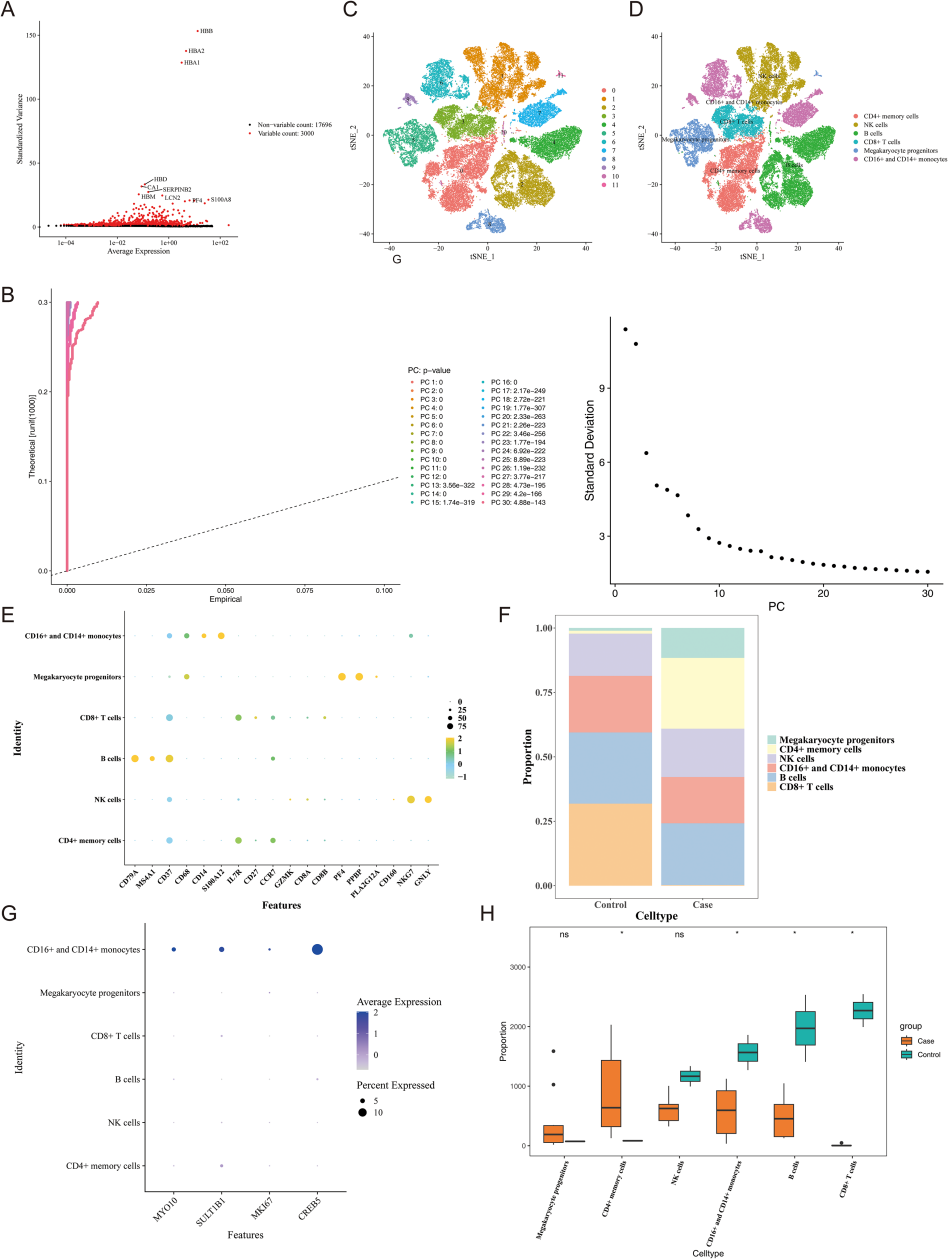
Single-cell RNA-seq analysis reveals immune cell composition in sepsis. **(A)** Highly variable genes identified based on expression dispersion. **(B)** PCA of variable genes across single cells. **(C)** t-SNE projection of cell clusters based on top principal components. **(D)** Annotated t-SNE plot showing major immune cell types. **(E)** Dot plot showing expression of marker genes across annotated cell types. **(F)** Cell type composition in control and sepsis groups. **(G)** Expression levels of *MYO10*, *SULT1B1*, *MKI67*, and *CREB5* across immune cell types. **(H)** Comparison of immune cell proportions between control and sepsis samples.ns means not significant, * means *P* < 0.05.

**Figure 11:**
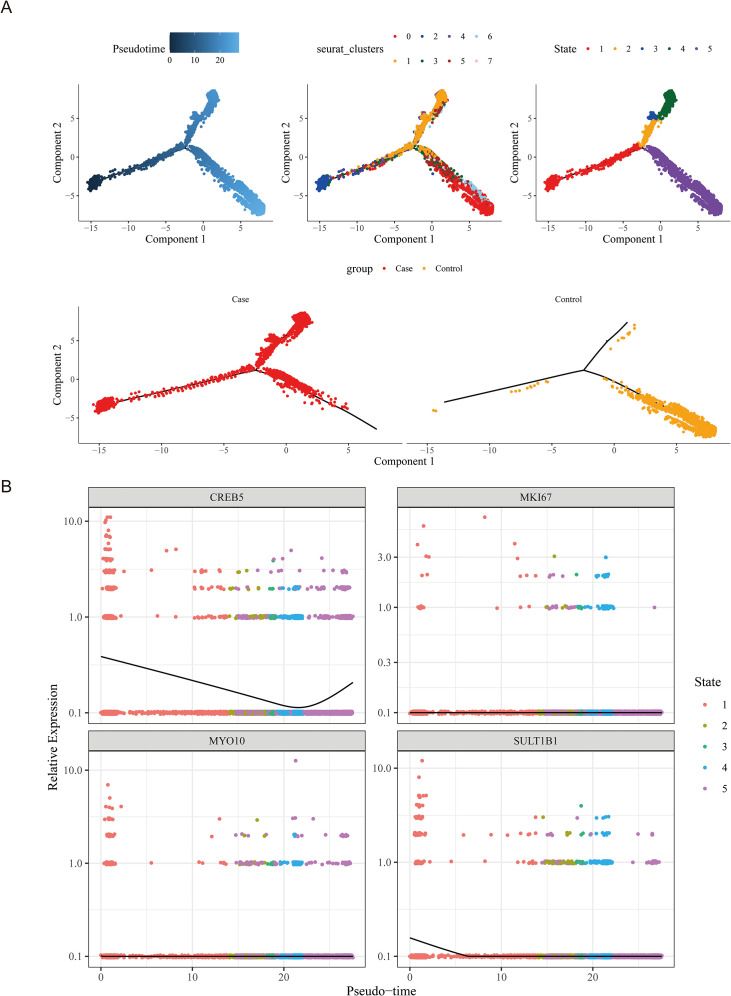
Pseudotime trajectory analysis of immune cell states in sepsis. **(A)** Pseudotime trajectories of single cells colored by pseudotime (top left), Seurat clusters (top center), cell states (top right), and experimental groups (bottom). **(B)** Expression trends of *CREB5*, *MKI67*, *MYO10*, and *SULT1B1* along the pseudotime axis.

After undergoing quality control, 47,080 cells and 20,696 genes were retained for further analysis (**Supplementary Figure S4**). Afterwards, the top 3000 hypervariable genes were selected for PCA (**Figure 10A**). In this study, the top 30 principal components were selected for cluster analysis, and all cells were ultimately divided into 12 clusters (**Figures 10B, C**). A total of 6 cells (B cells, CD16+ and CD14+ monocytes, CD4+memory cells, CD8+ T cells, Megakaryocyte progenitors, NK cells) were annotated based on the expression of marker genes in different cell clusters (**Figure 10D**). Simultaneously, the expressions of marker genes were shown in the six cell types (**Figure 10E**). To further demonstrate the expression of marker genes in each cell cluster, we generated a heatmap of the top 5 marker genes with the highest expression levels in each cluster (**Supplementary Figure S5**), aiming to more intuitively reflect the gene expression characteristics of different cell clusters. The cell proportion bar stack graph shows the high specificity of the marker gene, confirming the accuracy of the annotation (**Figure 10F**). To identify the key cell type in this study, we analyzed the expression levels of biomarkers in different cells. The results showed that compared with other immune cell types, all four biomarkers exhibited high expression in CD16+ and CD14+ monocytes (**Figure 10G**). We further compared the infiltration differences of each cell type between the sepsis group and the control group using the Wilcoxon test (*P* < 0.05), and found that there were significant differences in CD4+ memory cells, B cells, CD16+ and CD14+ monocytes, and CD8+ T cells between the two groups (**Figure 10H**). By synthesizing the evidence from both the above-mentioned biomarker expression profiles and cell infiltration differences, we finally identified CD16+ and CD14+ monocytes as the key cells. After secondary clustering, key cells were divided into seven clusters of key cells (**Supplementary Figure S6**). In pseudo-time analysis, a total of 5 underwent distinct states of CD16+ and CD14+ monocytes were discovered, and the expression of the cell was reduced over time in the disease group (**Figure 11A**).

During the process of cell differentiation, *MKI67* and *MYO10* showed no significant changes, while the expression level of *CREB5* first decreased and then increased, mostly distributed in the state 1 stage, which was the early stage of differentiation. The expression levels of *SULT1B1* first decreased and then stabilized, mostly distributed in the state 1 stage. The results indicated that the development of diseases might be closely related to *CREB5* and *SULT1B1* (**Figure 11B**).

### 3.10 The expression analysis of biomarkers in the clinical samples

**Figure 12:**
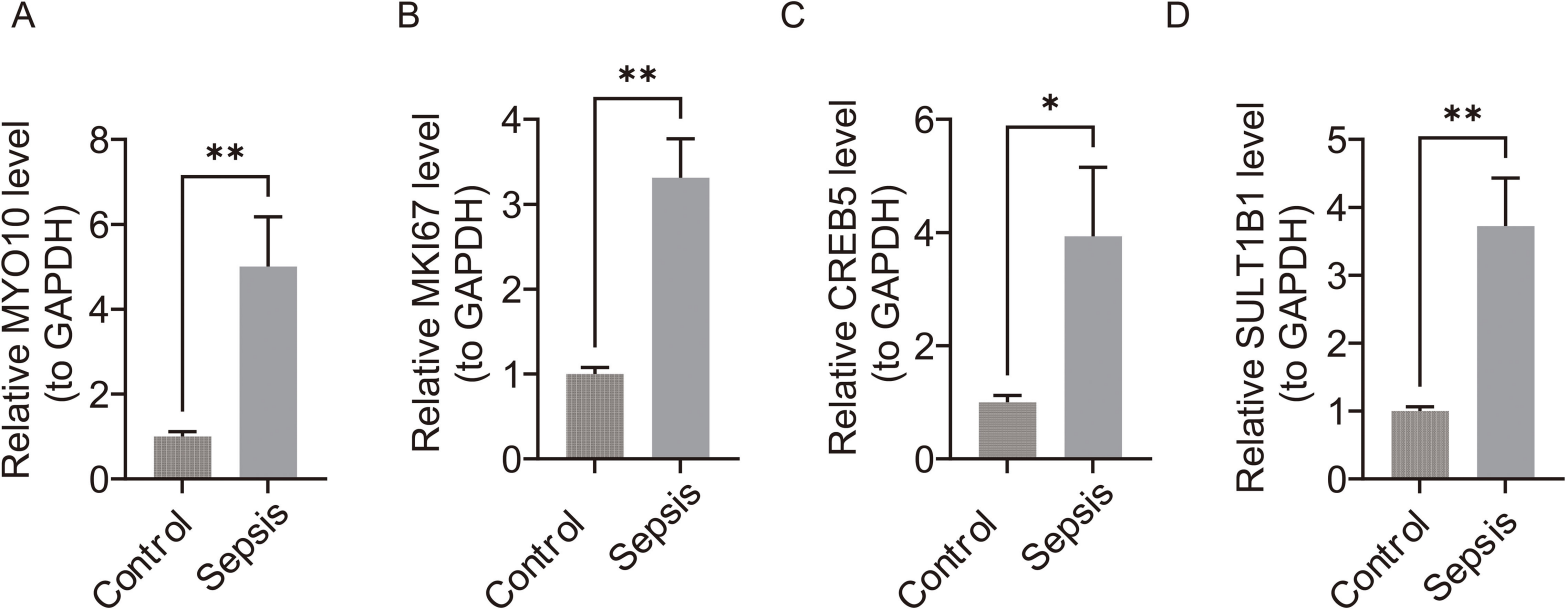
The validation of the expression levels of biomarkers. **(A)***MYO10.***(B)***MKI67*. **(C)***CREB5.***(D)***SULT1B1*. * means *P* < 0.05, ** means *P* < 0.01.

As shown in the **Figure 12**, The RT-qPCR results showed the expression of *MYO10*, *SULT1B1*, *MKI67*, and *CREB5* had significant differences between controls and sepsis samples (*P* < 0.05). The expression of 4 biomarkers were all upregulated in the sepsis group which were consistent in the results with the bioinformatics analysis results, indicating that preliminary results were reliable in our study.

## 4 Discussion

Sepsis is typically triggered by infection, leading to an excessive reaction of the body’s immune system that damages its own tissues and organs. Immune cells play a critical role in the onset and development of sepsis. In sepsis, immune cell phenotypes influence susceptibility and mortality, and telomere shortening is closely associated with the pathological processes of sepsis—such shortening may induce a phenotype resembling accelerated aging in survivors (43, 44). Mendelian randomization studies have confirmed a potential causal relationship between genetically predicted telomere shortening and increased sepsis susceptibility (42). In-depth research on the association between immune cells and telomeres can provide new directions for deciphering the pathological mechanisms of sepsis and improving its diagnosis and treatment. In this study, 34 candidate genes were obtained through steps including differentially expressed gene screening, WGCNA analysis, and intersection gene acquisition. Subsequently, four biomarkers were identified using 101-machine learning algorithms, ROC curve analysis, and gene expression profiling.

The potential mechanisms of the four biomarkers (*MYO10*, *SULT1B1*, *MKI67*, *CREB5*) identified in this study in sepsis can be preliminarily interpreted through their known functions and related pathways. *MYO10*, It is a key regulator of mitosis and genome stability (45). Although not yet directly implicated in sepsis, it may participate in the disease process by influencing immune-cell proliferation and the SHH/WNT signaling pathways (45). For example, abnormal activation and apoptotic imbalance of immune cells in sepsis may be related to its regulation of the mitotic checkpoint, while dysregulation of the SHH/WNT pathway may further exacerbate endothelial barrier damage or organ repair disorders, suggesting the potential role of *MYO10* in sepsis-induced multiple organ dysfunction (46–48). *SULT1B1*, a critical enzyme in sulfur metabolism and hormone modification (49), may affect common metabolic disorders in sepsis patients (such as non-thyroidal illness syndrome) by regulating thyroid hormone metabolism. Its sulfonation reaction may also modify inflammatory mediators (e.g., chemokines), thereby regulating the intensity of systemic inflammatory responses. Although this gene has been confirmed to participate in metabolic reprogramming in liver and gastric cancers, its role in sepsis remains to be further validated, particularly whether it indirectly regulates inflammatory signals through the NF-κB or MAPK pathways (50, 51). *MKI67*, a classical proliferation marker, may have a dual role in the sepsis immune microenvironment: high expression may occur during early excessive activation of immune cells, while decreased proliferative capacity may accompany the late immunosuppressive state. This dynamic change may reflect disease severity and prognosis. Its enrichment in the cell cycle and p53 pathways suggests that it may influence sepsis progression by regulating the balance between immune cell proliferation and exhaustion, similar to its role in the tumor microenvironment (52, 53). *CREB5*, a key transcription factor of the cAMP-signaling pathway, plays an important role in the initiation and progression of multiple cancers (54).Although direct research on *CREB5* in sepsis is currently limited, existing evidence suggests that it may be involved in immunomodulatory processes. For example, studies have found that miR-582-5p/miR-590-5p can induce monocyte immunosuppression by targeting the CREB1/CREB5-NF-κB signaling pathway cascade that ultimately drives monocytic immunosuppression (55), suggesting that *CREB5* may act as a regulator of the immune imbalance characteristic of sepsis. Furthermore, the functions of CREB5 in cell proliferation and survival (56), as well as its identified role in immunotherapy resistance, also suggest that it may have a potential role in sepsis-related cell damage repair and immunosuppressive processes. In the future, further exploration is needed into the specific molecular mechanisms of *CREB5* in sepsis and its clinical significance (57–59).

In summary, these biomarkers may be involved in sepsis through mechanisms related to cell cycle regulation (*MYO10*, *MKI67*), metabolic reprogramming (*SULT1B1*), and inflammatory signal transduction (*CREB5*). While their mechanisms share some commonalities with functions in other diseases, they may also exhibit specificity due to the unique pathological environment of sepsis (e.g., systemic inflammation, immune paralysis). For example, the genomic stability regulation of *MYO10* may be associated with apoptosis resistance caused by mitochondrial damage in immune cells during sepsis, while the metabolic modification function of *SULT1B1* may be involved in sepsis-related disorders of adrenocortical hormone metabolism (60). Future studies should validate the expression patterns and functions of these genes through *in vitro* and *in vivo* experiments, and use single-cell sequencing to clarify their dynamic changes in specific immune cell subsets (e.g., neutrophils, monocytes) to reveal their potential as diagnostic markers or therapeutic targets. Additionally, evaluating the efficacy of small-molecule inhibitors targeting *CREB5* or *MYO10* in sepsis models may provide a theoretical basis for developing novel therapies targeting immune metabolism or cell cycle regulation.

Literature on *SULT1B1* involvement in sepsis-related adrenocortical hormone metabolic disorders shows: Recent studies have confirmed that ribosomal dysfunction and FcγR-mediated phagocytic abnormalities play critical roles in sepsis. The former downregulates the ribosomal biosynthesis pathway in peripheral blood monocytes of patients, reducing IL-10 synthesis and exacerbating immune imbalance (61), consistent with the findings in this study that *MYO10*, *MKI67*, and other genes regulate protein synthesis through the ribosomal pathway to affect immune cell compensatory capacity; the latter has dual effects, where overactivated FcγR signaling induces endothelial cell apoptosis via reactive oxygen species (ROS) burst, while inhibiting this pathway alleviates lung injury, echoing the hypothesis that *MYO10* and *CREB5* regulate phagocytic efficiency through actin remodeling (62). In terms of metabolic regulation, SULT1A1 modifies the Fc segment of IgG to enhance its binding to FcγR for pathogen clearance, providing evidence for a similar immune-regulatory mechanism of *SULT1B1* in the FcγR phagocytic pathway (63). Cluster analysis divides sepsis patients into two groups: Cluster 2 has an increased proportion of plasma B cells, linked to *MKI67*-driven B cell differentiation, though their hyperactivation or exhaustion may impair immunity (61, 62); Cluster 1 shows increased neutrophil infiltration, associated with activation of the *CREB5*/*SULT1B1*-regulated chemokine pathway, whose phagocytic function is coordinately regulated by the FcγR pathway and *MYO10*/*CREB5 (*61, 63). Additionally, B cells and neutrophils interact via factors such as IgG, IL-6, and IL-10, with this balance disrupted in different subgroups, leading to immune phenotype polarization—Cluster 1 favors an inflammatory response, while Cluster 2 favors a compensatory antibody response (61).

In this study, through regulatory network analysis, it was found that four key biomarkers are potentially regulated by multiple miRNAs (including hsa-mir-218-5p) as well as a large number of TFs, indicating that their expression may be subject to precise regulation at the complex transcriptional and post-transcriptional levels. These findings indicate that the identified TFs and ncRNAs themselves could serve as potential intervention targets for the aforementioned biomarkers. Moreover, drug prediction based on the DSigDB database identified the class I–selective histone deacetylase (HDAC) inhibitor MS-275 (entinostat) as a candidate compound predicted to interact with both *MYO10* and *CREB5*.Notably, existing studies have shown that MS-275, as a class I-specific histone deacetylase (HDAC) inhibitor, may affect energy metabolism to a certain extent and accelerate the migration of cells across endothelial cell monolayers (64).existing studies have shown that MS-275, as a class I-specific histone deacetylase (HDAC) inhibitor, may affect energy metabolism and accelerate cell migration through endothelial cell monolayers to a moderate extent. MS-275 has also been investigated as a radiosensitizer for treating inherently radioresistant PAX3-FOXO1 rhabdomyosarcoma. Literature further indicates that MS-275 inhibits Robo4 expression by suppressing HDAC3 in endothelial cells and enhances endothelial and vascular permeability (65).

Single-cell sequencing analysis suggests that CD16+ and CD14+ monocytes are key immune subgroups in the pathogenesis of sepsis. Although this study found that the proportion of cells of these four biomarkers in this monocyte subpopulation was not high, it is important to interpret the single cell data in depth. Studies have shown that gene expression has inherent heterogeneity in cell populations, especially the expression of key regulatory genes is often limited to subpopulations of cells with specific functional states or activation stages (66). CD16+/CD14+ monocytes represent the primary convergence point for overlapping signaling pathways among these four biomarkers, including cytoskeletal regulation, metabolic processes, proliferation, and inflammatory responses. During sepsis, significant changes in the abundance of this cell population, combined with coordinated expression patterns within the biomarker panel, suggest that a functionally specialized monocyte subpopulation undergoes critical functional reprogramming essential to sepsis pathogenesis. This finding is consistent with the known biological properties of CD16+ monocytes as a key driver of inflammation (67, 68). In the complex pathogenesis of sepsis, CD16+/CD14+ monocytes play a central role in their functional reprogramming and abnormal association with multiple signaling pathways, including cytoskeletal regulation, metabolism, proliferation and inflammatory signaling pathways. Secondary clustering and pseudotime analysis of monocytes showed that *CREB5* and *SULT1B1* tended to be stable in their five differentiation states after significant downregulation in early differentiation, suggesting that they may be involved in the progression of sepsis. Studies have shown that significant changes in the composition and function of peripheral blood monocytes (PBMCs) occur in patients with sepsis (69). In patients with severe sepsis and septic shock, the proportion of CD14+CD16+ monocytes was significantly elevated (70). These findings demonstrate that CD16+/CD14+ monocytes act as central regulators of sepsis through dysfunctional pathways involving inflammation and immune metabolism. Targeting these cells or their key regulatory factors could provide novel therapeutic approaches, though this requires validation in future clinical cohort studies to optimize intervention strategies (71–73).

This study found that *CREB5* was significantly upregulated in sepsis, and was specifically enriched in CD16+ and CD14+ monocytes, and its expression level showed dynamic changes with cell differentiation. Gene set enrichment analysis suggested that *CREB5* was involved in ribosome and chemokine signaling pathways. As a key transcription factor in the cAMP signaling pathway, *CREB*5 recognizes and binds to the cAMP-responsive element in the promoter region of the target gene through the basic leucine zipper domain, and then regulates gene transcription reprogramming (58, 59). In the early stage of sepsis, the upregulation of *CREB5* may enhance the transcriptional activity of inflammatory factors such as IL-6 and TNF-α by directly binding to the promoter region of inflammatory factors, thus driving the inflammatory response of CD16+ monocytes. Its expression pattern during differentiation follows a “first decrease then increase” dynamic pattern, suggesting that *CREB5* may exert dual regulatory roles at different disease stages. Additionally, *CREB5* might influence monocyte metabolic reprogramming by affecting the transcription of glycolysis-related genes (59), thereby regulating their immune functions. In conclusion, *CREB5* is not only a biomarker, but its DNA binding ability may play a central role in the integration of septic stress signals and the reprogramming of immune cell function. Future studies should further clarify the downstream gene network of *CREB5* to further reveal its specific mechanism in sepsis.

Multi-omics analysis reveals that four biomarkers—*MYO10*, *SULT1B1*, *MKI67*, and *CREB5*—are upregulated in sepsis patients and hold potential as therapeutic targets: *MYO10*, an actin regulator, may be involved in monocyte migration and adhesion; *SULT1B1* shows early downregulation followed by stabilization during monocyte differentiation, and its reduced lipopolysaccharide(LPS) detoxification capacity exacerbates inflammation, suggesting that targeting its activation could enhance metabolic function; upregulation of *MKI67* indicates that abnormal monocyte proliferation drives inflammation, and inhibiting its expression may alleviate systemic inflammation (74); *CREB5* exhibits a biphasic expression pattern during differentiation, with early downregulation exacerbating oxidative damage and late upregulation exerting compensatory anti-inflammatory effects via antioxidant genes, and activating its pathway can induce monocyte polarization toward an anti-inflammatory phenotype to reduce organ damage (75).

This study identified immune cell- and telomere-related biomarkers in sepsis based on mRNA transcriptome data and their expression levels at the single-cell level, providing a theoretical basis for understanding sepsis and guiding subsequent diagnosis and treatment of sepsis patients. However, this study still has some limitations. First, all analyses were based on retrospective data from a public database with a relatively small sample size. Although the results confirm changes in biomarker expression levels in septic patients as a whole, this approach does not distinguish which immune cells these transcripts are derived from, and there is no independent external cohort validation. Secondly, while single-cell transcriptomic analysis has revealed expression dynamics of these genes in immune cell subpopulations, their specific functional mechanisms in sepsis remain unverified through experimental approaches such as gene knockout or overexpression. This lack of experimental validation prevents definitive causal relationships from being established. Furthermore, although single-cell sequencing suggests CD16+/CD14+ monocytes may be a critical source, whole blood data cannot provide direct experimental evidence to support this hypothesis. Furthermore, the predicted candidate drug MS-275’s interaction with genes has only been preliminarily evaluated through molecular docking, lacking support from *in vitro* and *in vivo* pharmacodynamic experiments. Future studies should validate the diagnostic efficacy of biomarkers in larger, multicenter prospective clinical cohorts. Flow cytometry should be employed to isolate key immune cell subpopulations (e.g., CD16+/CD14+ monocytes), enabling confirmation of their expression sources at the cellular level. Combined with single-cell proteomics technology, this approach will verify mRNA-protein level consistency, thereby enhancing the reliability of conclusions. Secondly, the mechanism must be deeply explained through functional experiments. *In vitro*, gene editing or overexpression technology can be used to regulate the expression of biomarkers in primary immune cells or cell lines, and observe their effects on immune response (such as inflammatory factor release, phagocytosis, cell migration and proliferation); *In vivo*, we can establish myeloid cell conditional knockout mouse models to clarify their causal role in the pathogenesis of sepsis. Finally, for the predicted candidate drug MS-275, systematic pharmacodynamic evaluations at the cellular level and efficacy validation in animal models should be conducted to determine its potential value in treating sepsis by modulating the aforementioned therapeutic targets. *In vivo*, we can establish myeloid cell conditional knockout mouse models to clarify their causal role in the pathogenesis of sepsis. Finally, for the predicted candidate drug MS-275, systematic pharmacodynamic evaluations at the cellular level and efficacy validation in animal models should be conducted to determine its potential value in treating sepsis by modulating the aforementioned therapeutic targets. These studies will help to promote the clinical translation of the biomarkers identified in this study from basic research to clinical applications.

## Data Availability

The original contributions presented in the study are included in the article/**Supplementary Material**. Further inquiries can be directed to the corresponding author.

## Supplementary material

Supplementary Figure 1 Three-dimensional scatter plot of case and control samples. 3D scatter plot based on dimensionality reduction.

Supplementary Figure 2 Immune cell composition in sepsis and control samples.

Supplementary Figure 3 Immune cell composition in sepsis subtypes.

Supplementary Figure 4 Quality control of single-cell RNA-seq data.

Supplementary Figure 5 Heat maps of the top5 marker genes in each cluster.

Supplementary Figure 6 Principal component analysis and dimensionality reduction of single-cell data. *JackStraw* plot (top) and scree plot (bottom left) were used to assess significance and variance explained by principal components. The t-SNE plot (bottom right) displays cell clusters projected from top-ranked PCs.

Supplementary Table 1 qPCR experimental setup and primer sequences. This table includes (A) the qPCR reaction system, (B) thermocycling conditions, and (C) sequences of primers used to detect *MYO10*, *SULT1B1*, *MKI67*, *CREB5*, and the internal reference gene GAPDH.

Supplementary Table 2 Gene Ontology (GO) biological process enrichment analysis of candidate genes. Listed are enriched GO-BP terms with their GO ID, term name, gene ratio, adjusted *P*-value, and core enrichment genes.

Supplementary Table 3 KEGG pathway enrichment analysis of candidate genes. Pathways were identified using the KEGG database. Listed are the top enriched pathways with adjusted *P* < 0.05.

## References

[1] Singer M Deutschman CS Seymour CW Shankar-Hari M Annane D Bauer M . The third international consensus definitions for sepsis and septic shock (Sepsis-3). Jama. (2016) 315:801–10. doi: 10.1001/jama.2016.0287, PMID: 26903338 PMC4968574

[2] Gao X Cai S Li X Wu G . Sepsis-induced immunosuppression: mechanisms, biomarkers and immunotherapy. Front Immunol. (2025) 16:1577105. doi: 10.3389/fimmu.2025.1577105, PMID: 40364841 PMC12069044

[3] Fleischmann C Scherag A Adhikari NK Hartog CS Tsaganos T Schlattmann P . Assessment of global incidence and mortality of hospital-treated sepsis. Current estimates and limitations. Am J Respir Crit Care Med. (2016) 193:259–72. doi: 10.1164/rccm.201504-0781OC, PMID: 26414292

[4] Schenz J Weigand MA Uhle F . Molecular and biomarker-based diagnostics in early sepsis: current challenges and future perspectives. Expert Rev Mol Diagn. (2019) 19:1069–78. doi: 10.1080/14737159.2020.1680285, PMID: 31608730

[5] van der Poll T Shankar-Hari M Wiersinga WJ . The immunology of sepsis. Immunity. (2021) 54:2450–64. doi: 10.1016/j.immuni.2021.10.012, PMID: 34758337

[6] Liu S Wang C Green G Zhuo H Liu KD Kangelaris KN . Peripheral blood leukocyte telomere length is associated with survival of sepsis patients. Eur Respir J. (2020) 55:1901044. doi: 10.1183/13993003.01044-2019, PMID: 31619475 PMC7359873

[7] Sacks D Baxter B Campbell BCV Carpenter JS Cognard C Dippel D . Multisociety consensus quality improvement revised consensus statement for endovascular therapy of acute ischemic stroke. Int J Stroke: Off J Int Stroke Soc. (2018) 13:612–32. doi: 10.1177/1747493018778713, PMID: 29786478

[8] Chen J Lin A Luo P . Advancing pharmaceutical research: A comprehensive review of cutting-edge tools and technologies. Curr Pharm Anal. (2024) 21:1–19. doi: 10.1016/j.cpan.2024.11.001

[9] Schmauck-Medina T Molière A Lautrup S Zhang J Chlopicki S Madsen HB . New hallmarks of ageing: A 2022 copenhagen ageing meeting summary. Aging. (2022) 14:6829–39. doi: 10.18632/aging.204248, PMID: 36040386 PMC9467401

[10] Niu X Wang B . A network medical framework based on inflammatory genes to identify drug candidates for abdominal aortic aneurysms. Curr Mol Pharmacol. (2024) 17:e170523216998. doi: 10.2174/1874467217666230517104426, PMID: 37198994

[11] Jiang T Zheng J Li N Li X He J Zhou J . Dissecting the mechanisms of intestinal immune homeostasis by analyzing T-cell immune response in crohn’s disease and colorectal cancer. Curr Gene Ther. (2024) 24:422–40. doi: 10.2174/0115665232294568240201073417, PMID: 38682449

[12] Goecks J Jalili V Heiser LM Gray JW . How machine learning will transform biomedicine. Cell. (2020) 181:92–101. doi: 10.1016/j.cell.2020.03.022, PMID: 32243801 PMC7141410

[13] Dara S Dhamercherla S Jadav SS Babu CM Ahsan MJ . Machine learning in drug discovery: A review. Artif Intell Rev. (2022) 55:1947–99. doi: 10.1007/s10462-021-10058-4, PMID: 34393317 PMC8356896

[14] Li J Kong Z Qi Y Wang W Su Q Huang W . Single-cell and bulk rna-sequence identified fibroblasts signature and cd8 + T-cell - fibroblast subtype predicting prognosis and immune therapeutic response of bladder cancer, based on machine learning: bioinformatics multi-omics study. Int J Surg (London England). (2024) 110:4911–31. doi: 10.1097/js9.0000000000001516, PMID: 38759695 PMC11325897

[15] Dai W Zheng P Wu J Chen S Deng M Tong X . Integrated analysis of single-cell rna-seq and chipset data unravels panoptosis-related genes in sepsis. Front Immunol. (2023) 14:1247131. doi: 10.3389/fimmu.2023.1247131, PMID: 38239341 PMC10795179

[16] Couch FJ McCarthy TV Gregg RG Hogan K . Dinucleotide repeat polymorphism at the D17s518 locus. Nucleic Acids Res. (1991) 19:5093. Available online at:https://pubmed.ncbi.nlm.nih.gov/1923789/, PMID: 1923789 PMC328837

[17] Qiu X Li J Bonenfant J Jaroszewski L Mittal A Klein W . Dynamic changes in human single-cell transcriptional signatures during fatal sepsis. J Leukocyte Biol. (2021) 110:1253–68. doi: 10.1002/jlb.5ma0721-825r, PMID: 34558746 PMC8629881

[18] Liu Z Liu X . Prognostic model of osteosarcoma based on telomere-related genes and analysis of immune characteristics. Int Immunopharmacol. (2025) 151:114198. doi: 10.1016/j.intimp.2025.114198, PMID: 39983416

[19] Sun Z Wang J Fan Z Yang Y Meng X Ma Z . Investigating the prognostic role of lncrnas associated with disulfidptosis-related genes in clear cell renal cell carcinoma. J Gene Med. (2024) 26:e3608. doi: 10.1002/jgm.3608, PMID: 37897262

[20] Love MI Huber W Anders S . Moderated estimation of fold change and dispersion for rna-seq data with deseq2. Genome Biol. (2014) 15:550. doi: 10.1186/s13059-014-0550-8, PMID: 25516281 PMC4302049

[21] Gustavsson EK Zhang D Reynolds RH Garcia-Ruiz S Ryten M . Ggtranscript: an R package for the visualization and interpretation of transcript isoforms using ggplot2. Bioinf (Oxford England). (2022) 38:3844–6. doi: 10.1093/bioinformatics/btac409, PMID: 35751589 PMC9344834

[22] Gu Z . Complex heatmap visualization. iMeta. (2022) 1:e43. doi: 10.1002/imt2.43, PMID: 38868715 PMC10989952

[23] Chen Y Huang W Ouyang J Wang J Xie Z . Identification of anoikis-related subgroups and prognosis model in liver hepatocellular carcinoma. Int J Mol Sci. (2023) 24:2862. doi: 10.3390/ijms24032862, PMID: 36769187 PMC9918018

[24] Rajalingam A Sekar K Ganjiwale A . Identification of potential genes and critical pathways in postoperative recurrence of crohn’s disease by machine learning and wgcna network analysis. Curr Genomics. (2023) 24:84–99. doi: 10.2174/1389202924666230601122334, PMID: 37994325 PMC10662376

[25] Yu G Wang LG Han Y He QY . Clusterprofiler: an R package for comparing biological themes among gene clusters. Omics: J Integr Biol. (2012) 16:284–7. doi: 10.1089/omi.2011.0118, PMID: 22455463 PMC3339379

[26] Wang W Hwang S Park D Park YD . The features of shared genes among transcriptomes probed in atopic dermatitis, psoriasis, and inflammatory acne: S100a9 selection as the target gene. Protein Pept Lett. (2024) 31:356–74. doi: 10.2174/0109298665290166240426072642, PMID: 38766834

[27] Engebretsen S Bohlin J . Statistical predictions with glmnet. Clin Epigenet. (2019) 11:123. doi: 10.1186/s13148-019-0730-1, PMID: 31443682 PMC6708235

[28] López-Díaz JÓM Méndez-González J López-Serrano PM Sánchez-Pérez FJ Méndez-Encina FM Mendieta-Oviedo R . Dummy regression to predict dry fiber in agave lechuguilla torr. In two large-scale bioclimatic regions in Mexico. PloS One. (2022) 17:e0274641. doi: 10.1371/journal.pone.0274641, PMID: 36108072 PMC9477326

[29] Hou N Li M He L Xie B Wang L Zhang R . Predicting 30-days mortality for mimic-iii patients with sepsis-3: A machine learning approach using xgboost. J Trans Med. (2020) 18:462. doi: 10.1186/s12967-020-02620-5, PMID: 33287854 PMC7720497

[30] Cavalcante T Ospina R Leiva V Cabezas X Martin-Barreiro C . Weibull regression and machine learning survival models: methodology, comparison, and application to biomedical data related to cardiac surgery. Biology. (2023) 12:442. doi: 10.3390/biology12030442, PMID: 36979135 PMC10045304

[31] Li K Yao S Zhang Z Cao B Wilson CM Kalos D . Efficient gradient boosting for prognostic biomarker discovery. Bioinf (Oxford England). (2022) 38:1631–8. doi: 10.1093/bioinformatics/btab869, PMID: 34978570 PMC10060728

[32] Yang L Pan X Zhang Y Zhao D Wang L Yuan G . Bioinformatics analysis to screen for genes related to myocardial infarction. Front Genet. (2022) 13:990888. doi: 10.3389/fgene.2022.990888, PMID: 36299582 PMC9589498

[33] Robin X Turck N Hainard A Tiberti N Lisacek F Sanchez JC . Proc: an open-source package for R and S+ to analyze and compare roc curves. BMC Bioinf. (2011) 12:77. doi: 10.1186/1471-2105-12-77, PMID: 21414208 PMC3068975

[34] Robles-Jimenez LE Aranda-Aguirre E Castelan-Ortega OA Shettino-Bermudez BS Ortiz-Salinas R Miranda M . Worldwide traceability of antibiotic residues from livestock in wastewater and soil: A systematic review. Anim: An Open Access J MDPI. (2021) 12:60. doi: 10.3390/ani12010060, PMID: 35011166 PMC8749557

[35] Zhang Z Cortese G Combescure C Marshall R Lee M Lim HJ . Overview of model validation for survival regression model with competing risks using melanoma study data. Ann Trans Med. (2018) 6:325. doi: 10.21037/atm.2018.07.38, PMID: 30364028 PMC6186983

[36] Li M Wei X Zhang SS Li S Chen SH Shi SJ . Recognition of refractory mycoplasma pneumoniae pneumonia among myocoplasma pneumoniae pneumonia in hospitalized children: development and validation of a predictive nomogram model. BMC Pulmonary Med. (2023) 23:383. doi: 10.1186/s12890-023-02684-1, PMID: 37817172 PMC10566172

[37] Zhang Y Zhao Z Huang W Kim BS Lin L Li X . Pan-cancer single-cell analysis revealing the heterogeneity of cancer-associated fibroblasts in skin tumors. Curr Gene Ther. (2024). doi: 10.2174/0115665232331353240911080642, PMID: 39323331

[38] Qing J Li C Hu X Song W Tirichen H Yaigoub H . Differentiation of T helper 17 cells may mediate the abnormal humoral immunity in iga nephropathy and inflammatory bowel disease based on shared genetic effects. Front Immunol. (2022) 13:916934. doi: 10.3389/fimmu.2022.916934, PMID: 35769467 PMC9234173

[39] Shannon P Markiel A Ozier O Baliga NS Wang JT Ramage D . Cytoscape: A software environment for integrated models of biomolecular interaction networks. Genome Res. (2003) 13:2498–504. doi: 10.1101/gr.1239303, PMID: 14597658 PMC403769

[40] Hao Y Hao S Andersen-Nissen E Mauck WM 3rd Zheng S Butler A . Integrated analysis of multimodal single-cell data. Cell. (2021) 184:3573–87.e29. doi: 10.1016/j.cell.2021.04.048, PMID: 34062119 PMC8238499

[41] Tu X Huang H Xu S Li C Luo S . Single-cell transcriptomics reveals immune infiltrate in sepsis. Front Pharmacol. (2023) 14:1133145. doi: 10.3389/fphar.2023.1133145, PMID: 37113759 PMC10126435

[42] Qiu X Mao Q Tang Y Wang L Chawla R Pliner HA . Reversed graph embedding resolves complex single-cell trajectories. Nat Methods. (2017) 14:979–82. doi: 10.1038/nmeth.4402, PMID: 28825705 PMC5764547

[43] Liu H Liu H Zhou L Wen S Liu T Ju L . The relationship between circulating immune cell phenotypes and sepsis: A mendelian randomization study. Shock (Augusta Ga). (2024) 61:577–84. doi: 10.1097/shk.0000000000002334, PMID: 38517244

[44] Oliveira NM Rios ECS de Lima TM Victorino VJ Barbeiro H Pinheiro da Silva F . Sepsis induces telomere shortening: A potential mechanism responsible for delayed pathophysiological events in sepsis survivors? Mol Med (Cambridge Mass). (2017) 22:886–91. doi: 10.2119/molmed.2016.00225, PMID: 27925632 PMC5319203

[45] Shangguan J Rock RS . Hundreds of myosin 10s are pushed to the tips of filopodia and could cause traffic jams on actin. eLife. (2024) 12:546598. doi: 10.7554/eLife.90603, PMID: 39480891 PMC11527427

[46] Hall ET Dillard ME Cleverdon ER Zhang Y Daly CA Ansari SS . Cytoneme signaling provides essential contributions to mammalian tissue patterning. Cell. (2024) 187:276–93.e23. doi: 10.1016/j.cell.2023.12.003, PMID: 38171360 PMC10842732

[47] Mayca Pozo F Geng X Miyagi M Amin AL Huang AY Zhang Y . Myo10 regulates genome stability and cancer inflammation through mediating mitosis. Cell Rep. (2023) 42:112531. doi: 10.1016/j.celrep.2023.112531, PMID: 37200188 PMC10293887

[48] Ou H Wang L Xi Z Shen H Jiang Y Zhou F . Myo10 contributes to the Malignant phenotypes of colorectal cancer via rack1 by activating integrin/src/fak signaling. Cancer Sci. (2022) 113:3838–51. doi: 10.1111/cas.15519, PMID: 35912545 PMC9633311

[49] Yu W Zhou R Li N Lei ZC Guo D Peng F . Author correction: histone tyrosine sulfation by sult1b1 regulates H4r3me2a and gene transcription. Nat Chem Biol. (2025), 855–64. doi: 10.1038/s41589-025-02029-5, PMID: 40890508

[50] Yu W Zhou R Li N Lei ZC Guo D Peng F . Histone tyrosine sulfation by sult1b1 regulates H4r3me2a and gene transcription. Nat Chem Biol. (2023) 19:855–64. doi: 10.1038/s41589-023-01267-9, PMID: 36805701

[51] Yin S Yu W Zhou R Zeng X Jiang L Wang Y . Histone H3y99sulf regulates hepatocellular carcinoma responding to hypoxia. J Biol Chem. (2024) 300:105721. doi: 10.1016/j.jbc.2024.105721, PMID: 38311175 PMC10910123

[52] Wu SY Liao P Yan LY Zhao QY Xie ZY Dong J . Correlation of mki67 with prognosis, immune infiltration, and T cell exhaustion in hepatocellular carcinoma. BMC Gastroenterol. (2021) 21:416. doi: 10.1186/s12876-021-01984-2, PMID: 34724892 PMC8561917

[53] Zhang A Wang X Fan C Mao X . The role of ki67 in evaluating neoadjuvant endocrine therapy of hormone receptor-positive breast cancer. Front Endocrinol. (2021) 12:687244. doi: 10.3389/fendo.2021.687244, PMID: 34803903 PMC8597938

[54] Wang S Qiu J Liu L Su C Qi L Huang C . Creb5 promotes invasiveness and metastasis in colorectal cancer by directly activating met. J Exp Clin Cancer Res: CR. (2020) 39:168. doi: 10.1186/s13046-020-01673-0, PMID: 32843066 PMC7446182

[55] Long X Li Y Qiu S Liu J He L Peng Y . Mir-582-5p/mir-590-5p targeted creb1/creb5-nf-κb signaling and caused opioid-induced immunosuppression in human monocytes. Trans Psychiatry. (2016) 6:e757. doi: 10.1038/tp.2016.4, PMID: 26978739 PMC4872460

[56] Yu T Zhang H Zhang C Ma G Shen T Luan Y . Creb5 promotes the proliferation of neural stem/progenitor cells in the rat subventricular zone via the regulation of nfix expression. Cells. (2025) 14:1240. doi: 10.3390/cells14161240, PMID: 40862718 PMC12384836

[57] Dinsdale RL Roache CE Meredith AL . Disease-associated kcnma1 variants decrease circadian clock robustness in channelopathy mouse models. J Gen Physiol. (2023) 155:e202313357. doi: 10.1085/jgp.202313357, PMID: 37728576 PMC10510740

[58] Kim HJ Jeon HM Batara DC Lee S Lee SJ Yin J . Creb5 promotes the proliferation and self-renewal ability of glioma stem cells. Cell Death Discov. (2024) 10:103. doi: 10.1038/s41420-024-01873-z, PMID: 38418476 PMC10901809

[59] Zhang CH Gao Y Hung HH Zhuo Z Grodzinsky AJ Lassar AB . Creb5 coordinates synovial joint formation with the genesis of articular cartilage. Nat Commun. (2022) 13:7295. doi: 10.1038/s41467-022-35010-0, PMID: 36435829 PMC9701237

[60] Choughule KV Locuson CW Coughtrie MW . Characterization of bovine phenol sulfotransferases: evidence of a major role for sult1b1 in the liver. Xenobiot Fate Foreign Compounds Biol Syst. (2015) 45:495–502. doi: 10.3109/00498254.2014.997325, PMID: 25539458

[61] Ni C Buszczak M . Ribosome biogenesis and function in development and disease. Dev (Cambridge England). (2023) 150:dev201187. doi: 10.1242/dev.201187, PMID: 36897354 PMC10108708

[62] Liu XS Zhou LM Yuan LL Gao Y Kui XY Liu XY . Npm1 is a prognostic biomarker involved in immune infiltration of lung adenocarcinoma and associated with M6a modification and glycolysis. Front Immunol. (2021) 12:724741. doi: 10.3389/fimmu.2021.724741, PMID: 34335635 PMC8324208

[63] Dang VD Stefanski AL Lino AC Dörner T . B- and plasma cell subsets in autoimmune diseases: translational perspectives. J Invest Dermatol. (2022) 142:811–22. doi: 10.1016/j.jid.2021.05.038, PMID: 34955289

[64] Deng Y Liu J Pu Z Wang Y Li T Jiang Z . Targeting the hla-E-nkg2a axis in combination with ms-275 enhances nk cell-based immunotherapy against dmg. J Exp Clin Cancer Res: CR. (2025) 44:133. doi: 10.1186/s13046-025-03390-y, PMID: 40296045 PMC12039099

[65] Kashio T Shirakura K Kinoshita M Morita M Ishiba R Muraoka K . Hdac inhibitor, ms-275, increases vascular permeability by suppressing robo4 expression in endothelial cells. Tissue Barriers. (2021) 9:1911195. doi: 10.1080/21688370.2021.1911195, PMID: 33955828 PMC8489956

[66] Papalexi E Satija R . Single-cell rna sequencing to explore immune cell heterogeneity. Nat Rev Immunol. (2018) 18:35–45. doi: 10.1038/nri.2017.76, PMID: 28787399

[67] Zhang T Fu JN Chen GB Zhang X . Plac8-erk pathway modulation of monocyte function in sepsis. Cell Death Discov. (2024) 10:308. doi: 10.1038/s41420-024-02012-4, PMID: 38961068 PMC11222481

[68] Qiu G Zheng G Ge M Huang L Tong H Chen P . Adipose-derived mesenchymal stem cells modulate cd14(++)Cd16(+) expression on monocytes from sepsis patients *in vitro* via prostaglandin E2. Stem Cell Res Ther. (2017) 8:97. doi: 10.1186/s13287-017-0546-x, PMID: 28446249 PMC5406890

[69] Wen M Cai G Ye J Liu X Ding H Zeng H . Single-cell transcriptomics reveals the alteration of peripheral blood mononuclear cells driven by sepsis. Ann Trans Med. (2020) 8:125. doi: 10.21037/atm.2020.02.35, PMID: 32175418 PMC7049046

[70] Fingerle G Pforte A Passlick B Blumenstein M Ströbel M Ziegler-Heitbrock HW . The novel subset of cd14+/cd16+ Blood monocytes is expanded in sepsis patients. Blood. (1993) 82:3170–6. doi: 10.1182/blood.V82.10.3170.3170, PMID: 7693040

[71] Vishnyakova P Kuznetsova M Poltavets A Fomina M Kiseleva V Muminova K . Distinct gene expression patterns for cd14++ and cd16++ Monocytes in preeclampsia. Sci Rep. (2022) 12:15469. doi: 10.1038/s41598-022-19847-5, PMID: 36104441 PMC9474473

[72] Ludwig K Chichelnitskiy E Kühne JF Wiegmann B Iske J Ledwoch N . Cd14(High)Cd16(+) monocytes are the main producers of interleukin-10 following clinical heart transplantation. Front Immunol. (2023) 14:1257526. doi: 10.3389/fimmu.2023.1257526, PMID: 37936714 PMC10627027

[73] Ziegler-Heitbrock L . The cd14+ Cd16+ Blood monocytes: their role in infection and inflammation. J Leukocyte Biol. (2007) 81:584–92. doi: 10.1189/jlb.0806510, PMID: 17135573

[74] Cui K Li Z . Identification and analysis of type 2 diabetes-mellitus-associated autophagy-related genes. Front Endocrinol. (2023) 14:1164112. doi: 10.3389/fendo.2023.1164112, PMID: 37223013 PMC10200926

[75] Zhao J He K Du H Wei G Wen Y Wang J . Bioinformatics prediction and experimental verification of key biomarkers for diabetic kidney disease based on transcriptome sequencing in mice. PeerJ. (2022) 10:e13932. doi: 10.7717/peerj.13932, PMID: 36157062 PMC9504448

