# Correction: Portfolio analysis of single-cell RNA-sequencing and transcriptomic data unravels immune cells and telomere-related biomarkers in sepsis

**DOI:** 10.3389/fimmu.2025.1746347

**Published:** 2025-12-10

**Authors:** Dan Chen, Xiyi Huang, Chun Wang, Cheng Zheng, Yunhao Liu

**Affiliations:** 1 Department of Environmental Health and Occupational Medicine, School of Public Health, Wuhan University of Science and Technology, Wuhan, China; 2 Department of Intensive Care Unit, The Central Hospital of Wuhan, Tongji Medical College, Huazhong University of Science and Technology, Wuhan, China; 3 Shantou University Medical College, Shantou, China

**Keywords:** sepsis, single-cell RNA sequencing, 101-machine learning, telomere, immune cells

Affiliation 3 “Shantou University Medical College, Shantou, China” was erroneously given as “Huazhong University of Science and Technology, Wuhan, China”.

The original version of this article has been updated.

