# Supplementary material for: Portfolio analysis of single-cell RNA-sequencing and transcriptomic data unravels immune cells and telomere-related biomarkers in sepsis
Source: Front Immunol. 2025 Oct 30;16:1638156. doi: 10.3389/fimmu.2025.1638156 (PMC12611921; doi:10.3389/fimmu.2025.1638156)
Supplement: Supplementary Figure 1 — Three-dimensional scatter plot of case and control samples. 3D scatter plot based on dimensionality reduction. [file Table1.docx]

Supplementary Material


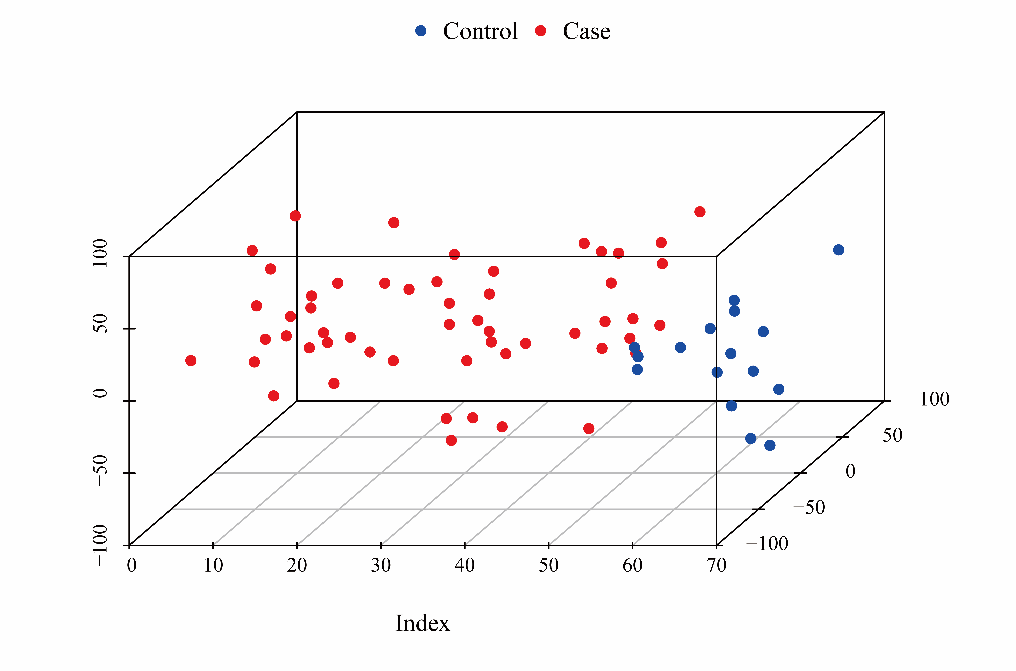


**Supplementary Figure S1. Three-dimensional scatter plot of case and control samples.** 3D scatter plot based on dimensionality reduction.


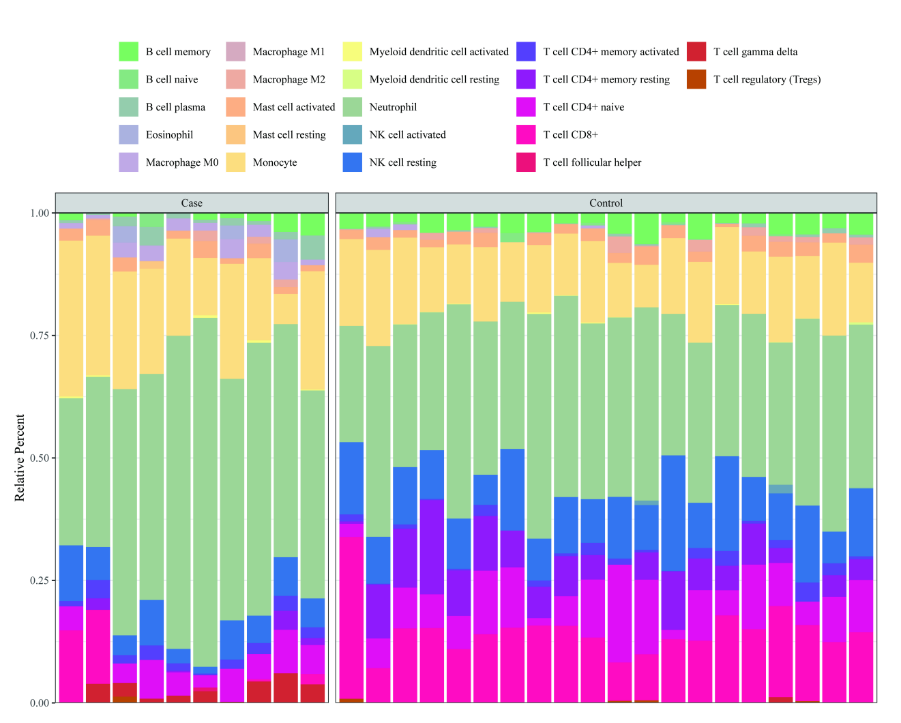


**Supplementary Figure S2. Immune cell composition in sepsis and control samples.**

**
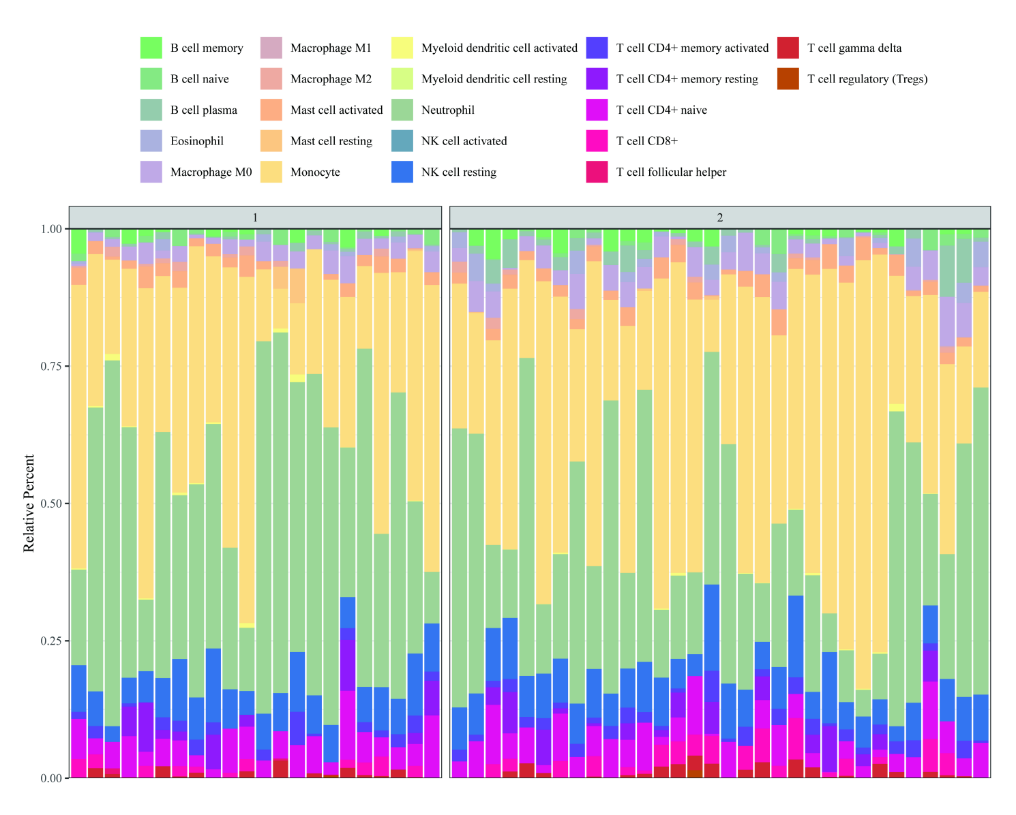
**

**Supplementary Figure S3. Immune cell composition in sepsis subtypes.**

**
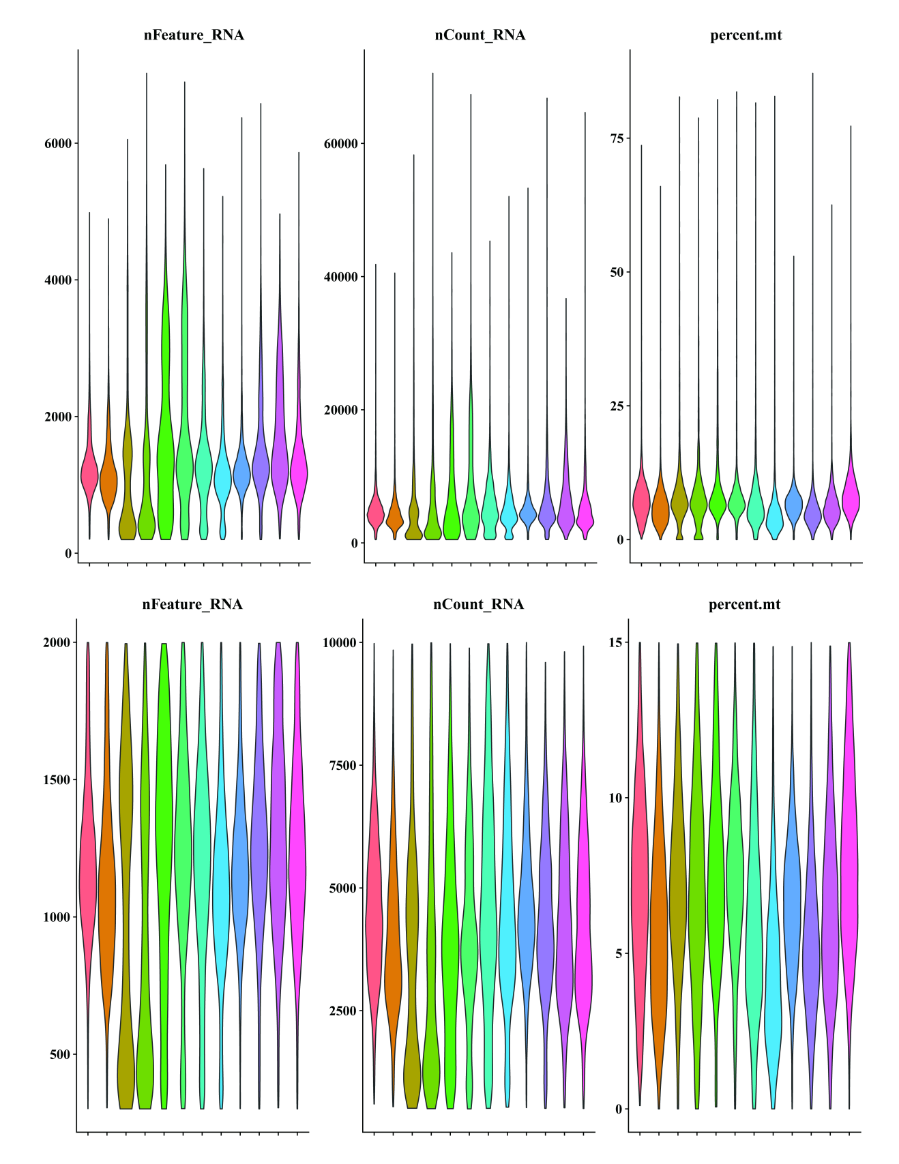
**

**Supplementary Figure S4. Quality control of single-cell RNA-seq data.**

**
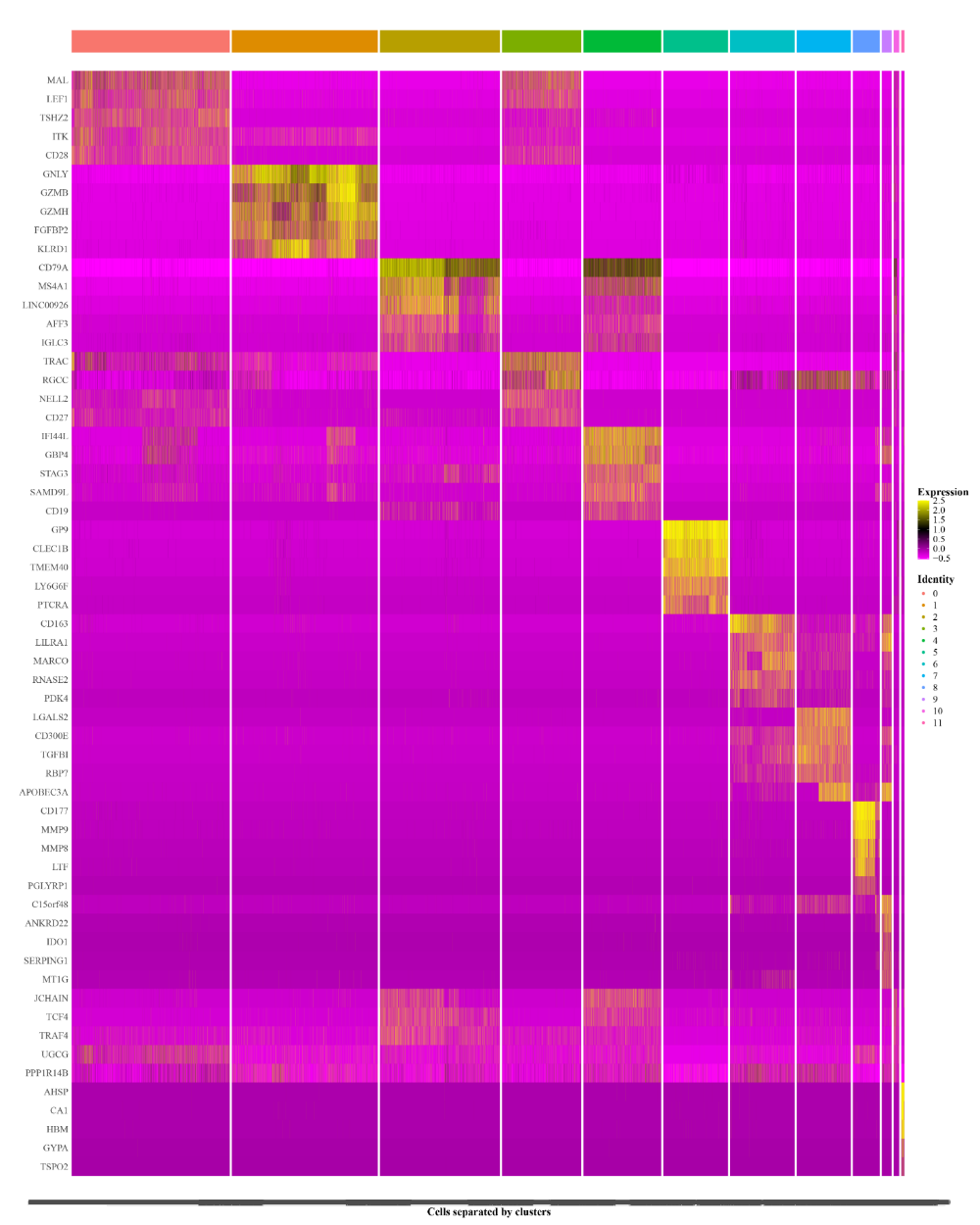
**

**Supplementary Figure S5. Heat maps of the top5 marker genes in each cluster.**

**
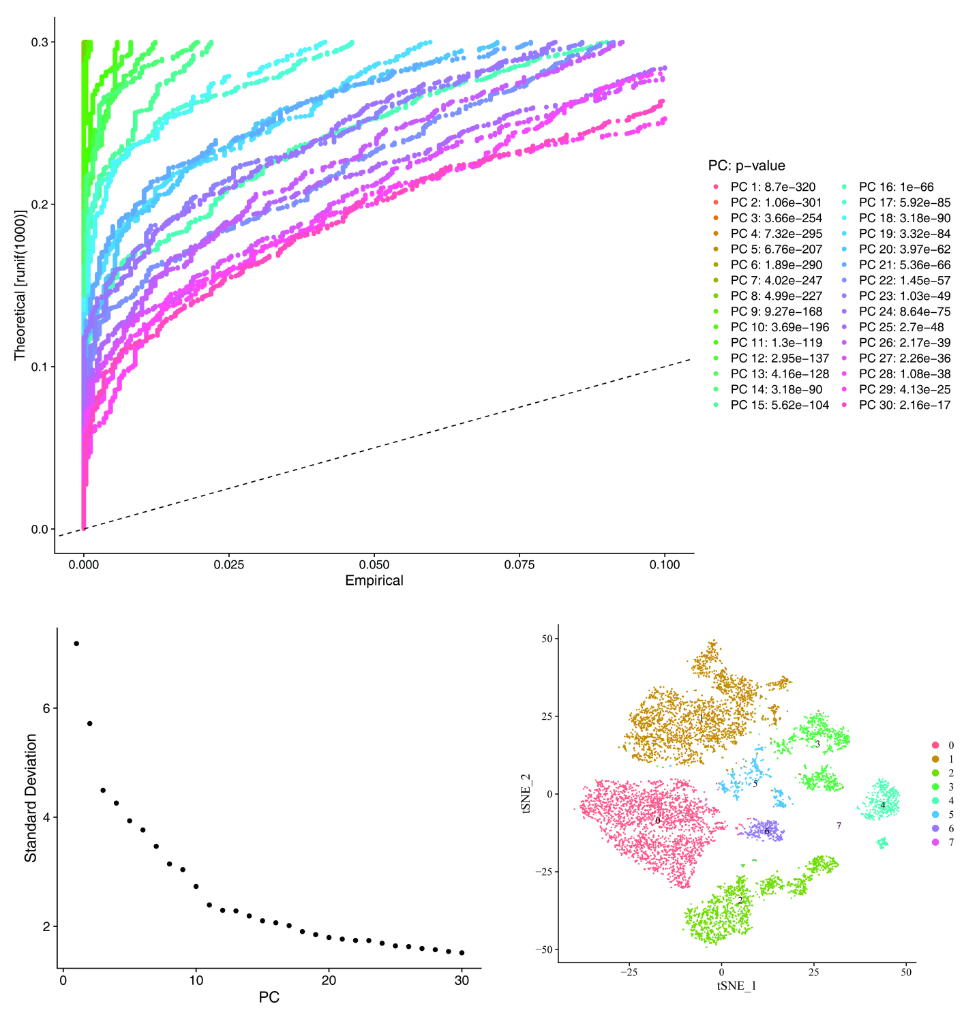
**

**Supplementary Figure S6. Principal component analysis and dimensionality reduction of single-cell data.***JackStraw* plot (top) and scree plot (bottom left) were used to assess significance and variance explained by principal components. The t-SNE plot (bottom right) displays cell clusters projected from top-ranked PCs.

**Supplementary Table S1. qPCR experimental setup and primer sequences.**

| **Reaction of qPCR** | |  |
| --- | --- | --- |
| Component | Volume |  |
| cDNA | 3ul |  |
| 2xUniversal Blue SYBR Green qPCR Master Mix | 5ul |  |
| Forward primer (10μM) | 1ul |  |
| Reverse primer (10μM) | 1ul |  |
|  |  |  |
| **Amplification conditions of PCR** | |  |
|  | temperatures | times |
| Pre denaturation | 95℃ | 1min |
| denaturation | 95℃ | 20s |
| annealing | 55℃ | 20s |
| extend | 72℃ | 30s |
|  |  |  |
| **Detailed information of primers** | |  |
| primers | sequences |  |
| MYO10 F | ACTTGGCCTCCTAGCCCTTA |  |
| MYO10 R | CTGCAACTCTGGGCTTCACA |  |
| SULT1B1 F | ATCCCCATGTGCAGCAAAGT |  |
| SULT1B1 R | ACAAGTAAGGGCCCAGGGTA |  |
| MKI67 F | GGCACTGTGACACGGTTCA |  |
| MKI67 R | TTCACCAAACAGGTAGTTGTTCA |  |
| CREB5 F | AGGGTTGGAGGCTAGACAGT |  |
| CREB5 R | GACTCTGGTACCTCCCTCGT |  |
| internal reference-GAPDH F | CGAAGGTGGAGTCAACGGATTT |  |
| internal reference-GAPDH R | ATGGGTGGAATCATATTGGAAC |  |

This table includes (A) the qPCR reaction system, (B) thermocycling conditions, and (C) sequences of primers used to detect MYO10, SULT1B1, MKI67, CREB5, and the internal reference gene GAPDH.

**Supplementary Table S2 Gene Ontology (GO) biological process enrichment analysis of candidate genes.**

| ONTOLOGY | ID | Description | BgRatio | pvalue | p.adjust | qvalue | geneID | Count | richFactor |
| --- | --- | --- | --- | --- | --- | --- | --- | --- | --- |
| BP | GO:0000280 | nuclear division | 446/18888 | 1.39E-09 | 1.32E-06 | 8.77E-07 | TDRD9/CDK1/CCNB1/MKI67/TOP2A/CDC20/AURKA/NEK2/CENPF/KIF4A | 10 | 0.224215247 |
| BP | GO:0048285 | organelle fission | 493/18888 | 3.63E-09 | 1.74E-06 | 1.15E-06 | TDRD9/CDK1/CCNB1/MKI67/TOP2A/CDC20/AURKA/NEK2/CENPF/KIF4A | 10 | 0.202839757 |
| BP | GO:0140014 | mitotic nuclear division | 277/18888 | 1.14E-08 | 3.63E-06 | 2.40E-06 | CDK1/CCNB1/MKI67/CDC20/AURKA/NEK2/CENPF/KIF4A | 8 | 0.288808664 |
| BP | GO:0007059 | chromosome segregation | 423/18888 | 1.70E-08 | 4.05E-06 | 2.68E-06 | CDK1/CCNB1/MKI67/TOP2A/CDC20/AURKA/NEK2/CENPF/KIF4A | 9 | 0.212765957 |
| BP | GO:0098813 | nuclear chromosome segregation | 317/18888 | 3.25E-08 | 5.40E-06 | 3.57E-06 | CDK1/CCNB1/TOP2A/CDC20/AURKA/NEK2/CENPF/KIF4A | 8 | 0.252365931 |
| BP | GO:0007088 | regulation of mitotic nuclear division | 118/18888 | 3.39E-08 | 5.40E-06 | 3.57E-06 | CCNB1/MKI67/CDC20/AURKA/NEK2/CENPF | 6 | 0.508474576 |
| BP | GO:0000819 | sister chromatid segregation | 229/18888 | 7.21E-08 | 9.84E-06 | 6.52E-06 | CDK1/CCNB1/TOP2A/CDC20/NEK2/CENPF/KIF4A | 7 | 0.305676856 |
| BP | GO:0033044 | regulation of chromosome organization | 247/18888 | 1.21E-07 | 1.33E-05 | 8.84E-06 | CDK1/CCNB1/TOP2A/CDC20/NEK2/CENPF/NBN | 7 | 0.28340081 |
| BP | GO:0051783 | regulation of nuclear division | 147/18888 | 1.26E-07 | 1.33E-05 | 8.84E-06 | CCNB1/MKI67/CDC20/AURKA/NEK2/CENPF | 6 | 0.408163265 |
| BP | GO:1902850 | microtubule cytoskeleton organization involved in mitosis | 164/18888 | 2.40E-07 | 2.30E-05 | 1.52E-05 | CDK1/CCNB1/CDC20/AURKA/NEK2/KIF4A | 6 | 0.365853659 |
| BP | GO:2001251 | negative regulation of chromosome organization | 93/18888 | 3.99E-07 | 3.46E-05 | 2.29E-05 | CCNB1/TOP2A/CDC20/CENPF/NBN | 5 | 0.537634409 |
| BP | GO:0000070 | mitotic sister chromatid segregation | 188/18888 | 5.37E-07 | 4.28E-05 | 2.83E-05 | CDK1/CCNB1/CDC20/NEK2/CENPF/KIF4A | 6 | 0.319148936 |
| BP | GO:0000075 | cell cycle checkpoint signaling | 191/18888 | 5.90E-07 | 4.33E-05 | 2.87E-05 | CDK1/CCNB1/MAPK14/CDC20/CENPF/NBN | 6 | 0.314136126 |
| BP | GO:0010948 | negative regulation of cell cycle process | 316/18888 | 6.39E-07 | 4.36E-05 | 2.89E-05 | CDK1/CCNB1/MAPK14/CDC20/NEK2/CENPF/NBN | 7 | 0.221518987 |
| BP | GO:0010389 | regulation of G2/M transition of mitotic cell cycle | 104/18888 | 6.97E-07 | 4.43E-05 | 2.94E-05 | CDK1/CCNB1/AURKA/CENPF/NBN | 5 | 0.480769231 |
| BP | GO:1902749 | regulation of cell cycle G2/M phase transition | 116/18888 | 1.20E-06 | 7.15E-05 | 4.74E-05 | CDK1/CCNB1/AURKA/CENPF/NBN | 5 | 0.431034483 |
| BP | GO:0033047 | regulation of mitotic sister chromatid segregation | 54/18888 | 1.77E-06 | 9.96E-05 | 6.60E-05 | CDK1/CCNB1/CDC20/CENPF | 4 | 0.740740741 |
| BP | GO:0007052 | mitotic spindle organization | 131/18888 | 2.19E-06 | 0.000109829 | 7.28E-05 | CCNB1/CDC20/AURKA/NEK2/KIF4A | 5 | 0.381679389 |
| BP | GO:0051983 | regulation of chromosome segregation | 131/18888 | 2.19E-06 | 0.000109829 | 7.28E-05 | CDK1/CCNB1/MKI67/CDC20/CENPF | 5 | 0.381679389 |
| BP | GO:0000086 | G2/M transition of mitotic cell cycle | 136/18888 | 2.63E-06 | 0.000125467 | 8.31E-05 | CDK1/CCNB1/AURKA/CENPF/NBN | 5 | 0.367647059 |
| BP | GO:0045786 | negative regulation of cell cycle | 405/18888 | 3.34E-06 | 0.000145957 | 9.67E-05 | CDK1/CCNB1/MAPK14/CDC20/NEK2/CENPF/NBN | 7 | 0.172839506 |
| BP | GO:0007093 | mitotic cell cycle checkpoint signaling | 143/18888 | 3.36E-06 | 0.000145957 | 9.67E-05 | CDK1/CCNB1/CDC20/CENPF/NBN | 5 | 0.34965035 |
| BP | GO:0044839 | cell cycle G2/M phase transition | 152/18888 | 4.53E-06 | 0.000186621 | 0.000123625 | CDK1/CCNB1/AURKA/CENPF/NBN | 5 | 0.328947368 |
| BP | GO:1901988 | negative regulation of cell cycle phase transition | 273/18888 | 4.69E-06 | 0.000186621 | 0.000123625 | CDK1/CCNB1/MAPK14/CDC20/CENPF/NBN | 6 | 0.21978022 |
| BP | GO:0051321 | meiotic cell cycle | 291/18888 | 6.76E-06 | 0.000258332 | 0.00017113 | TDRD9/TOP2A/CDC20/AURKA/NEK2/NBN | 6 | 0.206185567 |
| BP | GO:1901987 | regulation of cell cycle phase transition | 455/18888 | 7.16E-06 | 0.000263099 | 0.000174288 | CDK1/CCNB1/MAPK14/CDC20/AURKA/CENPF/NBN | 7 | 0.153846154 |
| BP | GO:1901991 | negative regulation of mitotic cell cycle phase transition | 195/18888 | 1.52E-05 | 0.00053833 | 0.000356612 | CDK1/CCNB1/CDC20/CENPF/NBN | 5 | 0.256410256 |
| BP | GO:0007051 | spindle organization | 197/18888 | 1.60E-05 | 0.000545309 | 0.000361235 | CCNB1/CDC20/AURKA/NEK2/KIF4A | 5 | 0.253807107 |
| BP | GO:1901990 | regulation of mitotic cell cycle phase transition | 356/18888 | 2.12E-05 | 0.000699719 | 0.000463523 | CDK1/CCNB1/CDC20/AURKA/CENPF/NBN | 6 | 0.168539326 |
| BP | GO:0033045 | regulation of sister chromatid segregation | 104/18888 | 2.43E-05 | 0.000774823 | 0.000513275 | CDK1/CCNB1/CDC20/CENPF | 4 | 0.384615385 |
| BP | GO:0060421 | positive regulation of heart growth | 41/18888 | 4.09E-05 | 0.001260202 | 0.00083481 | CDK1/MAPK14/BASP1 | 3 | 0.731707317 |
| BP | GO:0045930 | negative regulation of mitotic cell cycle | 248/18888 | 4.82E-05 | 0.00143775 | 0.000952425 | CDK1/CCNB1/CDC20/CENPF/NBN | 5 | 0.201612903 |
| BP | GO:0051225 | spindle assembly | 129/18888 | 5.66E-05 | 0.001497487 | 0.000991998 | CDC20/AURKA/NEK2/KIF4A | 4 | 0.310077519 |
| BP | GO:0007094 | mitotic spindle assembly checkpoint signaling | 46/18888 | 5.79E-05 | 0.001497487 | 0.000991998 | CCNB1/CDC20/CENPF | 3 | 0.652173913 |
| BP | GO:0071173 | spindle assembly checkpoint signaling | 46/18888 | 5.79E-05 | 0.001497487 | 0.000991998 | CCNB1/CDC20/CENPF | 3 | 0.652173913 |
| BP | GO:0071174 | mitotic spindle checkpoint signaling | 46/18888 | 5.79E-05 | 0.001497487 | 0.000991998 | CCNB1/CDC20/CENPF | 3 | 0.652173913 |
| BP | GO:0045931 | positive regulation of mitotic cell cycle | 130/18888 | 5.84E-05 | 0.001497487 | 0.000991998 | CDK1/CCNB1/CDC20/AURKA | 4 | 0.307692308 |
| BP | GO:0031577 | spindle checkpoint signaling | 47/18888 | 6.18E-05 | 0.001497487 | 0.000991998 | CCNB1/CDC20/CENPF | 3 | 0.638297872 |
| BP | GO:0033046 | negative regulation of sister chromatid segregation | 48/18888 | 6.59E-05 | 0.001497487 | 0.000991998 | CCNB1/CDC20/CENPF | 3 | 0.625 |
| BP | GO:0033048 | negative regulation of mitotic sister chromatid segregation | 48/18888 | 6.59E-05 | 0.001497487 | 0.000991998 | CCNB1/CDC20/CENPF | 3 | 0.625 |
| BP | GO:0045841 | negative regulation of mitotic metaphase/anaphase transition | 48/18888 | 6.59E-05 | 0.001497487 | 0.000991998 | CCNB1/CDC20/CENPF | 3 | 0.625 |
| BP | GO:2000816 | negative regulation of mitotic sister chromatid separation | 48/18888 | 6.59E-05 | 0.001497487 | 0.000991998 | CCNB1/CDC20/CENPF | 3 | 0.625 |
| BP | GO:0051985 | negative regulation of chromosome segregation | 50/18888 | 7.45E-05 | 0.001572317 | 0.001041568 | CCNB1/CDC20/CENPF | 3 | 0.6 |
| BP | GO:1902100 | negative regulation of metaphase/anaphase transition of cell cycle | 50/18888 | 7.45E-05 | 0.001572317 | 0.001041568 | CCNB1/CDC20/CENPF | 3 | 0.6 |
| BP | GO:1905819 | negative regulation of chromosome separation | 50/18888 | 7.45E-05 | 0.001572317 | 0.001041568 | CCNB1/CDC20/CENPF | 3 | 0.6 |
| BP | GO:0061982 | meiosis I cell cycle process | 139/18888 | 7.57E-05 | 0.001572317 | 0.001041568 | TDRD9/TOP2A/CDC20/AURKA | 4 | 0.287769784 |
| BP | GO:0044772 | mitotic cell cycle phase transition | 451/18888 | 7.95E-05 | 0.00161521 | 0.001069982 | CDK1/CCNB1/CDC20/AURKA/CENPF/NBN | 6 | 0.133037694 |
| BP | GO:0046622 | positive regulation of organ growth | 53/18888 | 8.87E-05 | 0.001764865 | 0.001169119 | CDK1/MAPK14/BASP1 | 3 | 0.566037736 |
| BP | GO:0045839 | negative regulation of mitotic nuclear division | 56/18888 | 0.000104616 | 0.00203894 | 0.001350678 | CCNB1/CDC20/CENPF | 3 | 0.535714286 |
| BP | GO:0010965 | regulation of mitotic sister chromatid separation | 59/18888 | 0.000122281 | 0.002335575 | 0.001547182 | CCNB1/CDC20/CENPF | 3 | 0.508474576 |
| BP | GO:0051306 | mitotic sister chromatid separation | 62/18888 | 0.000141785 | 0.002655001 | 0.001758783 | CCNB1/CDC20/CENPF | 3 | 0.483870968 |
| BP | GO:0051784 | negative regulation of nuclear division | 63/18888 | 0.000148709 | 0.002731106 | 0.001809198 | CCNB1/CDC20/CENPF | 3 | 0.476190476 |
| BP | GO:0060420 | regulation of heart growth | 73/18888 | 0.000230339 | 0.004068937 | 0.002695432 | CDK1/MAPK14/BASP1 | 3 | 0.410958904 |
| BP | GO:0090307 | mitotic spindle assembly | 73/18888 | 0.000230339 | 0.004068937 | 0.002695432 | CDC20/NEK2/KIF4A | 3 | 0.410958904 |
| BP | GO:0007100 | mitotic centrosome separation | 14/18888 | 0.000234337 | 0.004068937 | 0.002695432 | AURKA/NEK2 | 2 | 1.428571429 |
| BP | GO:1905818 | regulation of chromosome separation | 74/18888 | 0.000239806 | 0.004089545 | 0.002709084 | CCNB1/CDC20/CENPF | 3 | 0.405405405 |
| BP | GO:0051299 | centrosome separation | 15/18888 | 0.000270112 | 0.00452556 | 0.002997919 | AURKA/NEK2 | 2 | 1.333333333 |
| BP | GO:0140013 | meiotic nuclear division | 197/18888 | 0.000289665 | 0.00476796 | 0.003158494 | TDRD9/TOP2A/CDC20/AURKA | 4 | 0.203045685 |
| BP | GO:0010639 | negative regulation of organelle organization | 365/18888 | 0.000294565 | 0.00476796 | 0.003158494 | CCNB1/TOP2A/CDC20/CENPF/NBN | 5 | 0.136986301 |
| BP | GO:0051304 | chromosome separation | 81/18888 | 0.000313185 | 0.004984856 | 0.003302175 | CCNB1/CDC20/CENPF | 3 | 0.37037037 |
| BP | GO:0032392 | DNA geometric change | 85/18888 | 0.00036095 | 0.005650938 | 0.003743416 | TOP2A/HMGB3/NBN | 3 | 0.352941176 |
| BP | GO:1903046 | meiotic cell cycle process | 218/18888 | 0.000425323 | 0.006472014 | 0.00428733 | TDRD9/TOP2A/CDC20/AURKA | 4 | 0.183486239 |
| BP | GO:0030071 | regulation of mitotic metaphase/anaphase transition | 90/18888 | 0.00042695 | 0.006472014 | 0.00428733 | CCNB1/CDC20/CENPF | 3 | 0.333333333 |
| BP | GO:0071103 | DNA conformation change | 92/18888 | 0.000455375 | 0.006795055 | 0.004501326 | TOP2A/HMGB3/NBN | 3 | 0.326086957 |
| BP | GO:1902099 | regulation of metaphase/anaphase transition of cell cycle | 93/18888 | 0.000470033 | 0.006905868 | 0.004574733 | CCNB1/CDC20/CENPF | 3 | 0.322580645 |
| BP | GO:0007091 | metaphase/anaphase transition of mitotic cell cycle | 94/18888 | 0.00048499 | 0.007017661 | 0.004648789 | CCNB1/CDC20/CENPF | 3 | 0.319148936 |
| BP | GO:0140694 | non-membrane-bounded organelle assembly | 413/18888 | 0.000518601 | 0.007392002 | 0.004896768 | CDC20/AURKA/NEK2/CENPF/KIF4A | 5 | 0.121065375 |
| BP | GO:0044784 | metaphase/anaphase transition of cell cycle | 97/18888 | 0.000531687 | 0.007431268 | 0.00492278 | CCNB1/CDC20/CENPF | 3 | 0.309278351 |
| BP | GO:0051988 | regulation of attachment of spindle microtubules to kinetochore | 21/18888 | 0.000536919 | 0.007431268 | 0.00492278 | CCNB1/NEK2 | 2 | 0.952380952 |
| BP | GO:0060419 | heart growth | 99/18888 | 0.000564363 | 0.007591084 | 0.005028649 | CDK1/MAPK14/BASP1 | 3 | 0.303030303 |
| BP | GO:1901992 | positive regulation of mitotic cell cycle phase transition | 99/18888 | 0.000564363 | 0.007591084 | 0.005028649 | CDK1/CCNB1/CDC20 | 3 | 0.303030303 |
| BP | GO:0051310 | metaphase chromosome alignment | 100/18888 | 0.000581173 | 0.007708614 | 0.005106505 | CCNB1/NEK2/CENPF | 3 | 0.3 |
| BP | GO:0046620 | regulation of organ growth | 102/18888 | 0.000615747 | 0.008055322 | 0.005336179 | CDK1/MAPK14/BASP1 | 3 | 0.294117647 |
| BP | GO:2001252 | positive regulation of chromosome organization | 110/18888 | 0.000767134 | 0.00990018 | 0.00655829 | CDK1/NEK2/NBN | 3 | 0.272727273 |
| BP | GO:0051303 | establishment of chromosome localization | 112/18888 | 0.00080835 | 0.010292986 | 0.006818501 | CCNB1/NEK2/CENPF | 3 | 0.267857143 |
| BP | GO:1904353 | regulation of telomere capping | 26/18888 | 0.000826709 | 0.010388255 | 0.006881611 | NEK2/NBN | 2 | 0.769230769 |
| BP | GO:0090068 | positive regulation of cell cycle process | 263/18888 | 0.000859896 | 0.010664942 | 0.0070649 | CDK1/CCNB1/CDC20/AURKA | 4 | 0.152091255 |
| BP | GO:0051984 | positive regulation of chromosome segregation | 27/18888 | 0.000891934 | 0.010920474 | 0.007234175 | CDK1/CCNB1 | 2 | 0.740740741 |
| BP | GO:1901989 | positive regulation of cell cycle phase transition | 117/18888 | 0.000917488 | 0.01109115 | 0.007347237 | CDK1/CCNB1/CDC20 | 3 | 0.256410256 |
| BP | GO:0010971 | positive regulation of G2/M transition of mitotic cell cycle | 28/18888 | 0.000959563 | 0.011454786 | 0.007588125 | CDK1/CCNB1 | 2 | 0.714285714 |
| BP | GO:0050000 | chromosome localization | 120/18888 | 0.000987245 | 0.011639737 | 0.007710644 | CCNB1/NEK2/CENPF | 3 | 0.25 |
| BP | GO:0000077 | DNA damage checkpoint signaling | 125/18888 | 0.001110824 | 0.01293703 | 0.008570025 | CDK1/MAPK14/NBN | 3 | 0.24 |
| BP | GO:0060045 | positive regulation of cardiac muscle cell proliferation | 31/18888 | 0.001176802 | 0.013379122 | 0.008862885 | CDK1/MAPK14 | 2 | 0.64516129 |
| BP | GO:1902751 | positive regulation of cell cycle G2/M phase transition | 31/18888 | 0.001176802 | 0.013379122 | 0.008862885 | CDK1/CCNB1 | 2 | 0.64516129 |
| BP | GO:0007127 | meiosis I | 131/18888 | 0.001271527 | 0.014285981 | 0.009463626 | TDRD9/TOP2A/AURKA | 3 | 0.229007634 |
| BP | GO:0031570 | DNA integrity checkpoint signaling | 134/18888 | 0.001357089 | 0.015070001 | 0.009982993 | CDK1/MAPK14/NBN | 3 | 0.223880597 |
| BP | GO:0007098 | centrosome cycle | 139/18888 | 0.001507604 | 0.016548988 | 0.010962736 | CDK1/AURKA/NEK2 | 3 | 0.215827338 |
| BP | GO:0007095 | mitotic G2 DNA damage checkpoint signaling | 37/18888 | 0.001675188 | 0.017975328 | 0.011907602 | CDK1/NBN | 2 | 0.540540541 |
| BP | GO:0055023 | positive regulation of cardiac muscle tissue growth | 37/18888 | 0.001675188 | 0.017975328 | 0.011907602 | CDK1/MAPK14 | 2 | 0.540540541 |
| BP | GO:0007143 | female meiotic nuclear division | 39/18888 | 0.001860033 | 0.019520126 | 0.01293094 | TOP2A/AURKA | 2 | 0.512820513 |
| BP | GO:0016233 | telomere capping | 39/18888 | 0.001860033 | 0.019520126 | 0.01293094 | NEK2/NBN | 2 | 0.512820513 |
| BP | GO:0031023 | microtubule organizing center organization | 152/18888 | 0.001946854 | 0.020085105 | 0.013305206 | CDK1/AURKA/NEK2 | 3 | 0.197368421 |
| BP | GO:0042307 | positive regulation of protein import into nucleus | 40/18888 | 0.001955932 | 0.020085105 | 0.013305206 | CDK1/MAPK14 | 2 | 0.5 |
| BP | GO:0045787 | positive regulation of cell cycle | 332/18888 | 0.002027775 | 0.020601328 | 0.013647174 | CDK1/CCNB1/CDC20/AURKA | 4 | 0.120481928 |
| BP | GO:0045840 | positive regulation of mitotic nuclear division | 44/18888 | 0.00236253 | 0.02374964 | 0.015732746 | CDC20/AURKA | 2 | 0.454545455 |
| BP | GO:0030261 | chromosome condensation | 45/18888 | 0.002469893 | 0.024570293 | 0.01627638 | CDK1/TOP2A | 2 | 0.444444444 |
| BP | GO:0048639 | positive regulation of developmental growth | 169/18888 | 0.002631261 | 0.025905712 | 0.017161017 | CDK1/MAPK14/BASP1 | 3 | 0.177514793 |
| BP | GO:0007517 | muscle organ development | 359/18888 | 0.002691851 | 0.026231812 | 0.017377039 | MAPK14/KLF5/CENPF/BASP1 | 4 | 0.111420613 |
| BP | GO:0060043 | regulation of cardiac muscle cell proliferation | 48/18888 | 0.002805582 | 0.027063945 | 0.017928279 | CDK1/MAPK14 | 2 | 0.416666667 |
| BP | GO:0035265 | organ growth | 180/18888 | 0.003144221 | 0.030027315 | 0.019891338 | CDK1/MAPK14/BASP1 | 3 | 0.166666667 |
| BP | GO:0060538 | skeletal muscle organ development | 182/18888 | 0.003243617 | 0.030669846 | 0.020316977 | MAPK14/KLF5/BASP1 | 3 | 0.164835165 |
| BP | GO:0008608 | attachment of spindle microtubules to kinetochore | 52/18888 | 0.003284624 | 0.030753098 | 0.020372127 | CCNB1/NEK2 | 2 | 0.384615385 |
| BP | GO:0044818 | mitotic G2/M transition checkpoint | 53/18888 | 0.003409954 | 0.031616566 | 0.020944124 | CDK1/NBN | 2 | 0.377358491 |
| BP | GO:0042770 | signal transduction in response to DNA damage | 188/18888 | 0.003553355 | 0.032629363 | 0.021615042 | CDK1/MAPK14/NBN | 3 | 0.159574468 |
| BP | GO:0046777 | protein autophosphorylation | 192/18888 | 0.00376959 | 0.034285316 | 0.022712014 | AURKA/NEK2/NBN | 3 | 0.15625 |
| BP | GO:0042306 | regulation of protein import into nucleus | 58/18888 | 0.004069666 | 0.036665383 | 0.024288669 | CDK1/MAPK14 | 2 | 0.344827586 |
| BP | GO:0009948 | anterior/posterior axis specification | 59/18888 | 0.00420817 | 0.037367504 | 0.024753783 | AURKA/BASP1 | 2 | 0.338983051 |
| BP | GO:0031099 | regeneration | 200/18888 | 0.004225854 | 0.037367504 | 0.024753783 | CDK1/KLF5/AURKA | 3 | 0.15 |
| BP | GO:0060038 | cardiac muscle cell proliferation | 60/18888 | 0.004348846 | 0.038102274 | 0.025240526 | CDK1/MAPK14 | 2 | 0.333333333 |
| BP | GO:0051785 | positive regulation of nuclear division | 61/18888 | 0.004491686 | 0.038995998 | 0.025832565 | CDC20/AURKA | 2 | 0.327868852 |
| BP | GO:0046824 | positive regulation of nucleocytoplasmic transport | 62/18888 | 0.004636682 | 0.039892177 | 0.026426231 | CDK1/MAPK14 | 2 | 0.322580645 |
| BP | GO:0031100 | animal organ regeneration | 66/18888 | 0.005238099 | 0.04426889 | 0.029325548 | CDK1/AURKA | 2 | 0.303030303 |
| BP | GO:0048255 | mRNA stabilization | 66/18888 | 0.005238099 | 0.04426889 | 0.029325548 | IGF2BP3/MAPK14 | 2 | 0.303030303 |
| BP | GO:0055021 | regulation of cardiac muscle tissue growth | 67/18888 | 0.005393776 | 0.045184702 | 0.02993222 | CDK1/MAPK14 | 2 | 0.298507463 |
| BP | GO:0060537 | muscle tissue development | 438/18888 | 0.005466834 | 0.045314082 | 0.030017926 | CDK1/MAPK14/KLF5/CENPF | 4 | 0.091324201 |
| BP | GO:0010972 | negative regulation of G2/M transition of mitotic cell cycle | 68/18888 | 0.005551568 | 0.045314082 | 0.030017926 | CDK1/NBN | 2 | 0.294117647 |
| BP | GO:0035825 | homologous recombination | 68/18888 | 0.005551568 | 0.045314082 | 0.030017926 | TOP2A/NBN | 2 | 0.294117647 |
| BP | GO:0031330 | negative regulation of cellular catabolic process | 224/18888 | 0.005791087 | 0.046868546 | 0.031047667 | IGF2BP3/MAPK14/RRAGD | 3 | 0.133928571 |
| BP | GO:1902750 | negative regulation of cell cycle G2/M phase transition | 70/18888 | 0.005873471 | 0.047135835 | 0.031224731 | CDK1/NBN | 2 | 0.285714286 |
| BP | GO:0032206 | positive regulation of telomere maintenance | 71/18888 | 0.006037567 | 0.047651872 | 0.031566575 | NEK2/NBN | 2 | 0.281690141 |
| BP | GO:0045600 | positive regulation of fat cell differentiation | 71/18888 | 0.006037567 | 0.047651872 | 0.031566575 | MAPK14/KLF5 | 2 | 0.281690141 |
| BP | GO:0043489 | RNA stabilization | 75/18888 | 0.006714762 | 0.052562277 | 0.034819431 | IGF2BP3/MAPK14 | 2 | 0.266666667 |
| BP | GO:1902373 | negative regulation of mRNA catabolic process | 76/18888 | 0.006889229 | 0.053489542 | 0.035433689 | IGF2BP3/MAPK14 | 2 | 0.263157895 |
| BP | GO:0032436 | positive regulation of proteasomal ubiquitin-dependent protein catabolic process | 77/18888 | 0.00706575 | 0.054417668 | 0.036048519 | CDC20/AURKA | 2 | 0.25974026 |
| BP | GO:0050807 | regulation of synapse organization | 243/18888 | 0.007247479 | 0.055370738 | 0.036679872 | LRRN1/MAPK14/CDC20 | 3 | 0.12345679 |
| BP | GO:0032508 | DNA duplex unwinding | 79/18888 | 0.007424925 | 0.056276216 | 0.037279697 | TOP2A/NBN | 2 | 0.253164557 |
| BP | GO:0050803 | regulation of synapse structure or activity | 249/18888 | 0.00774858 | 0.058266879 | 0.038598395 | LRRN1/MAPK14/CDC20 | 3 | 0.120481928 |
| BP | GO:0005996 | monosaccharide metabolic process | 252/18888 | 0.008006664 | 0.059737216 | 0.039572408 | MAPK14/PGD/PGM2 | 3 | 0.119047619 |
| BP | GO:0002437 | inflammatory response to antigenic stimulus | 84/18888 | 0.008358331 | 0.06093287 | 0.040364459 | MAPK14/IL1RN | 2 | 0.238095238 |
| BP | GO:0014855 | striated muscle cell proliferation | 84/18888 | 0.008358331 | 0.06093287 | 0.040364459 | CDK1/MAPK14 | 2 | 0.238095238 |
| BP | GO:0044773 | mitotic DNA damage checkpoint signaling | 84/18888 | 0.008358331 | 0.06093287 | 0.040364459 | CDK1/NBN | 2 | 0.238095238 |
| BP | GO:0045927 | positive regulation of growth | 258/18888 | 0.008538016 | 0.061771255 | 0.040919839 | CDK1/MAPK14/BASP1 | 3 | 0.11627907 |
| BP | GO:0044774 | mitotic DNA integrity checkpoint signaling | 88/18888 | 0.009141097 | 0.064966318 | 0.043036381 | CDK1/NBN | 2 | 0.227272727 |
| BP | GO:1902369 | negative regulation of RNA catabolic process | 88/18888 | 0.009141097 | 0.064966318 | 0.043036381 | IGF2BP3/MAPK14 | 2 | 0.227272727 |
| BP | GO:0014706 | striated muscle tissue development | 265/18888 | 0.00918372 | 0.064966318 | 0.043036381 | CDK1/MAPK14/CENPF | 3 | 0.113207547 |
| BP | GO:0055017 | cardiac muscle tissue growth | 91/18888 | 0.009748917 | 0.068457466 | 0.045349063 | CDK1/MAPK14 | 2 | 0.21978022 |
| BP | GO:1900182 | positive regulation of protein localization to nucleus | 94/18888 | 0.010374322 | 0.072317351 | 0.04790601 | CDK1/MAPK14 | 2 | 0.212765957 |
| BP | GO:0090316 | positive regulation of intracellular protein transport | 95/18888 | 0.010586667 | 0.073262806 | 0.048532319 | CDK1/MAPK14 | 2 | 0.210526316 |
| BP | GO:0018105 | peptidyl-serine phosphorylation | 282/18888 | 0.010869064 | 0.074675946 | 0.049468441 | CDK1/MAPK14/AURKA | 3 | 0.106382979 |
| BP | GO:0009798 | axis specification | 99/18888 | 0.011455263 | 0.078141261 | 0.05176401 | AURKA/BASP1 | 2 | 0.202020202 |
| BP | GO:1903312 | negative regulation of mRNA metabolic process | 100/18888 | 0.011677184 | 0.079090144 | 0.052392589 | IGF2BP3/MAPK14 | 2 | 0.2 |
| BP | GO:0042773 | ATP synthesis coupled electron transport | 102/18888 | 0.012126704 | 0.08008909 | 0.053054333 | CDK1/CCNB1 | 2 | 0.196078431 |
| BP | GO:0042775 | mitochondrial ATP synthesis coupled electron transport | 102/18888 | 0.012126704 | 0.08008909 | 0.053054333 | CDK1/CCNB1 | 2 | 0.196078431 |
| BP | GO:2000060 | positive regulation of ubiquitin-dependent protein catabolic process | 102/18888 | 0.012126704 | 0.08008909 | 0.053054333 | CDC20/AURKA | 2 | 0.196078431 |
| BP | GO:0018209 | peptidyl-serine modification | 294/18888 | 0.012160124 | 0.08008909 | 0.053054333 | CDK1/MAPK14/AURKA | 3 | 0.102040816 |
| BP | GO:0051054 | positive regulation of DNA metabolic process | 301/18888 | 0.012952481 | 0.084723423 | 0.056124307 | CDK1/NEK2/NBN | 3 | 0.099667774 |
| BP | GO:0032204 | regulation of telomere maintenance | 106/18888 | 0.013048289 | 0.084769499 | 0.056154829 | NEK2/NBN | 2 | 0.188679245 |
| BP | GO:1901800 | positive regulation of proteasomal protein catabolic process | 109/18888 | 0.013759003 | 0.088782758 | 0.058813379 | CDC20/AURKA | 2 | 0.183486239 |
| BP | GO:0048144 | fibroblast proliferation | 110/18888 | 0.013999594 | 0.089728939 | 0.059440168 | CDK1/CCNB1 | 2 | 0.181818182 |
| BP | GO:0051146 | striated muscle cell differentiation | 313/18888 | 0.014378692 | 0.09154434 | 0.060642765 | CDK1/MAPK14/KLF5 | 3 | 0.095846645 |
| BP | GO:0046822 | regulation of nucleocytoplasmic transport | 112/18888 | 0.014486264 | 0.091618428 | 0.060691844 | CDK1/MAPK14 | 2 | 0.178571429 |
| BP | GO:0042752 | regulation of circadian rhythm | 114/18888 | 0.014980215 | 0.094119111 | 0.0623484 | CDK1/TOP2A | 2 | 0.175438596 |
| BP | GO:0001824 | blastocyst development | 117/18888 | 0.015734679 | 0.095474825 | 0.063246481 | NEK2/NBN | 2 | 0.170940171 |
| BP | GO:0006655 | phosphatidylglycerol biosynthetic process | 10/18888 | 0.016295703 | 0.095474825 | 0.063246481 | PGS1 | 1 | 1 |
| BP | GO:0035646 | endosome to melanosome transport | 10/18888 | 0.016295703 | 0.095474825 | 0.063246481 | RAB32 | 1 | 1 |
| BP | GO:0043485 | endosome to pigment granule transport | 10/18888 | 0.016295703 | 0.095474825 | 0.063246481 | RAB32 | 1 | 1 |
| BP | GO:0048757 | pigment granule maturation | 10/18888 | 0.016295703 | 0.095474825 | 0.063246481 | RAB32 | 1 | 1 |
| BP | GO:0055015 | ventricular cardiac muscle cell development | 10/18888 | 0.016295703 | 0.095474825 | 0.063246481 | CDK1 | 1 | 1 |
| BP | GO:0070391 | response to lipoteichoic acid | 10/18888 | 0.016295703 | 0.095474825 | 0.063246481 | MAPK14 | 1 | 1 |
| BP | GO:0071223 | cellular response to lipoteichoic acid | 10/18888 | 0.016295703 | 0.095474825 | 0.063246481 | MAPK14 | 1 | 1 |
| BP | GO:1904383 | response to sodium phosphate | 10/18888 | 0.016295703 | 0.095474825 | 0.063246481 | ALPL | 1 | 1 |
| BP | GO:1990253 | cellular response to leucine starvation | 10/18888 | 0.016295703 | 0.095474825 | 0.063246481 | RRAGD | 1 | 1 |
| BP | GO:2000659 | regulation of interleukin-1-mediated signaling pathway | 10/18888 | 0.016295703 | 0.095474825 | 0.063246481 | IL1RN | 1 | 1 |
| BP | GO:0048638 | regulation of developmental growth | 330/18888 | 0.016547241 | 0.096095891 | 0.063657901 | CDK1/MAPK14/BASP1 | 3 | 0.090909091 |
| BP | GO:0022904 | respiratory electron transport chain | 123/18888 | 0.017291742 | 0.096095891 | 0.063657901 | CDK1/CCNB1 | 2 | 0.162601626 |
| BP | GO:0015980 | energy derivation by oxidation of organic compounds | 338/18888 | 0.017628263 | 0.096095891 | 0.063657901 | CDK1/CCNB1/PGM2 | 3 | 0.088757396 |
| BP | GO:0006310 | DNA recombination | 339/18888 | 0.017766126 | 0.096095891 | 0.063657901 | TOP2A/HMGB3/NBN | 3 | 0.088495575 |
| BP | GO:0032434 | regulation of proteasomal ubiquitin-dependent protein catabolic process | 125/18888 | 0.017824844 | 0.096095891 | 0.063657901 | CDC20/AURKA | 2 | 0.16 |
| BP | GO:0031915 | positive regulation of synaptic plasticity | 11/18888 | 0.017911067 | 0.096095891 | 0.063657901 | CDC20 | 1 | 0.909090909 |
| BP | GO:0034115 | negative regulation of heterotypic cell-cell adhesion | 11/18888 | 0.017911067 | 0.096095891 | 0.063657901 | IL1RN | 1 | 0.909090909 |
| BP | GO:0042451 | purine nucleoside biosynthetic process | 11/18888 | 0.017911067 | 0.096095891 | 0.063657901 | PGM2 | 1 | 0.909090909 |
| BP | GO:0042455 | ribonucleoside biosynthetic process | 11/18888 | 0.017911067 | 0.096095891 | 0.063657901 | PGM2 | 1 | 0.909090909 |
| BP | GO:0046129 | purine ribonucleoside biosynthetic process | 11/18888 | 0.017911067 | 0.096095891 | 0.063657901 | PGM2 | 1 | 0.909090909 |
| BP | GO:0051256 | mitotic spindle midzone assembly | 11/18888 | 0.017911067 | 0.096095891 | 0.063657901 | KIF4A | 1 | 0.909090909 |
| BP | GO:0051987 | positive regulation of attachment of spindle microtubules to kinetochore | 11/18888 | 0.017911067 | 0.096095891 | 0.063657901 | CCNB1 | 1 | 0.909090909 |
| BP | GO:0070587 | regulation of cell-cell adhesion involved in gastrulation | 11/18888 | 0.017911067 | 0.096095891 | 0.063657901 | IL1RN | 1 | 0.909090909 |
| BP | GO:0070934 | CRD-mediated mRNA stabilization | 11/18888 | 0.017911067 | 0.096095891 | 0.063657901 | IGF2BP3 | 1 | 0.909090909 |
| BP | GO:0141007 | siRNA-mediated retrotransposon silencing by heterochromatin formation | 11/18888 | 0.017911067 | 0.096095891 | 0.063657901 | TDRD9 | 1 | 0.909090909 |
| BP | GO:0021700 | developmental maturation | 341/18888 | 0.01804368 | 0.096266561 | 0.06377096 | CDC20/AURKA/RAB32 | 3 | 0.08797654 |
| BP | GO:0006275 | regulation of DNA replication | 128/18888 | 0.018637533 | 0.098133101 | 0.065007433 | CDK1/NBN | 2 | 0.15625 |
| BP | GO:1903052 | positive regulation of proteolysis involved in protein catabolic process | 131/18888 | 0.019465719 | 0.098133101 | 0.065007433 | CDC20/AURKA | 2 | 0.152671756 |
| BP | GO:0000022 | mitotic spindle elongation | 12/18888 | 0.019523863 | 0.098133101 | 0.065007433 | KIF4A | 1 | 0.833333333 |
| BP | GO:0006068 | ethanol catabolic process | 12/18888 | 0.019523863 | 0.098133101 | 0.065007433 | SULT1B1 | 1 | 0.833333333 |
| BP | GO:0043174 | nucleoside salvage | 12/18888 | 0.019523863 | 0.098133101 | 0.065007433 | PGM2 | 1 | 0.833333333 |
| BP | GO:0043476 | pigment accumulation | 12/18888 | 0.019523863 | 0.098133101 | 0.065007433 | RAB32 | 1 | 0.833333333 |
| BP | GO:0043482 | cellular pigment accumulation | 12/18888 | 0.019523863 | 0.098133101 | 0.065007433 | RAB32 | 1 | 0.833333333 |
| BP | GO:0070586 | cell-cell adhesion involved in gastrulation | 12/18888 | 0.019523863 | 0.098133101 | 0.065007433 | IL1RN | 1 | 0.833333333 |
| BP | GO:0071233 | cellular response to leucine | 12/18888 | 0.019523863 | 0.098133101 | 0.065007433 | RRAGD | 1 | 0.833333333 |
| BP | GO:0071493 | cellular response to UV-B | 12/18888 | 0.019523863 | 0.098133101 | 0.065007433 | MAPK14 | 1 | 0.833333333 |
| BP | GO:0090306 | meiotic spindle assembly | 12/18888 | 0.019523863 | 0.098133101 | 0.065007433 | AURKA | 1 | 0.833333333 |
| BP | GO:0009895 | negative regulation of catabolic process | 353/18888 | 0.019760249 | 0.098801245 | 0.06545004 | IGF2BP3/MAPK14/RRAGD | 3 | 0.084985836 |
| BP | GO:0014902 | myotube differentiation | 133/18888 | 0.020026373 | 0.099610344 | 0.06598602 | MAPK14/KLF5 | 2 | 0.15037594 |
| BP | GO:0051384 | response to glucocorticoid | 134/18888 | 0.020309241 | 0.100493911 | 0.066571332 | ALPL/IL1RN | 2 | 0.149253731 |
| BP | GO:0007144 | female meiosis I | 13/18888 | 0.021134096 | 0.10245209 | 0.067868511 | AURKA | 1 | 0.769230769 |
| BP | GO:0009812 | flavonoid metabolic process | 13/18888 | 0.021134096 | 0.10245209 | 0.067868511 | SULT1B1 | 1 | 0.769230769 |
| BP | GO:0019321 | pentose metabolic process | 13/18888 | 0.021134096 | 0.10245209 | 0.067868511 | PGD | 1 | 0.769230769 |
| BP | GO:0032530 | regulation of microvillus organization | 13/18888 | 0.021134096 | 0.10245209 | 0.067868511 | KLF5 | 1 | 0.769230769 |
| BP | GO:0032388 | positive regulation of intracellular transport | 140/18888 | 0.02204165 | 0.103291122 | 0.06842432 | CDK1/MAPK14 | 2 | 0.142857143 |
| BP | GO:0043467 | regulation of generation of precursor metabolites and energy | 140/18888 | 0.02204165 | 0.103291122 | 0.06842432 | CDK1/CCNB1 | 2 | 0.142857143 |
| BP | GO:0035331 | negative regulation of hippo signaling | 14/18888 | 0.02274177 | 0.103291122 | 0.06842432 | MAPK14 | 1 | 0.714285714 |
| BP | GO:0045842 | positive regulation of mitotic metaphase/anaphase transition | 14/18888 | 0.02274177 | 0.103291122 | 0.06842432 | CDC20 | 1 | 0.714285714 |
| BP | GO:0051231 | spindle elongation | 14/18888 | 0.02274177 | 0.103291122 | 0.06842432 | KIF4A | 1 | 0.714285714 |
| BP | GO:0051255 | spindle midzone assembly | 14/18888 | 0.02274177 | 0.103291122 | 0.06842432 | KIF4A | 1 | 0.714285714 |
| BP | GO:0055012 | ventricular cardiac muscle cell differentiation | 14/18888 | 0.02274177 | 0.103291122 | 0.06842432 | CDK1 | 1 | 0.714285714 |
| BP | GO:0072075 | metanephric mesenchyme development | 14/18888 | 0.02274177 | 0.103291122 | 0.06842432 | BASP1 | 1 | 0.714285714 |
| BP | GO:1900180 | regulation of protein localization to nucleus | 144/18888 | 0.023229686 | 0.103291122 | 0.06842432 | CDK1/MAPK14 | 2 | 0.138888889 |
| BP | GO:0051347 | positive regulation of transferase activity | 376/18888 | 0.023296767 | 0.103291122 | 0.06842432 | CDC20/NEK2/NBN | 3 | 0.079787234 |
| BP | GO:0045598 | regulation of fat cell differentiation | 146/18888 | 0.023833492 | 0.103291122 | 0.06842432 | MAPK14/KLF5 | 2 | 0.136986301 |
| BP | GO:0001325 | formation of extrachromosomal circular DNA | 15/18888 | 0.024346888 | 0.103291122 | 0.06842432 | NBN | 1 | 0.666666667 |
| BP | GO:0005980 | glycogen catabolic process | 15/18888 | 0.024346888 | 0.103291122 | 0.06842432 | PGM2 | 1 | 0.666666667 |
| BP | GO:0010457 | centriole-centriole cohesion | 15/18888 | 0.024346888 | 0.103291122 | 0.06842432 | NEK2 | 1 | 0.666666667 |
| BP | GO:0030174 | regulation of DNA-templated DNA replication initiation | 15/18888 | 0.024346888 | 0.103291122 | 0.06842432 | NBN | 1 | 0.666666667 |
| BP | GO:0034310 | primary alcohol catabolic process | 15/18888 | 0.024346888 | 0.103291122 | 0.06842432 | SULT1B1 | 1 | 0.666666667 |
| BP | GO:0034501 | protein localization to kinetochore | 15/18888 | 0.024346888 | 0.103291122 | 0.06842432 | CDK1 | 1 | 0.666666667 |
| BP | GO:0043101 | purine-containing compound salvage | 15/18888 | 0.024346888 | 0.103291122 | 0.06842432 | PGM2 | 1 | 0.666666667 |
| BP | GO:0043201 | response to leucine | 15/18888 | 0.024346888 | 0.103291122 | 0.06842432 | RRAGD | 1 | 0.666666667 |
| BP | GO:0044771 | meiotic cell cycle phase transition | 15/18888 | 0.024346888 | 0.103291122 | 0.06842432 | CDC20 | 1 | 0.666666667 |
| BP | GO:0048308 | organelle inheritance | 15/18888 | 0.024346888 | 0.103291122 | 0.06842432 | CDK1 | 1 | 0.666666667 |
| BP | GO:0048313 | Golgi inheritance | 15/18888 | 0.024346888 | 0.103291122 | 0.06842432 | CDK1 | 1 | 0.666666667 |
| BP | GO:0090656 | t-circle formation | 15/18888 | 0.024346888 | 0.103291122 | 0.06842432 | NBN | 1 | 0.666666667 |
| BP | GO:0090737 | telomere maintenance via telomere trimming | 15/18888 | 0.024346888 | 0.103291122 | 0.06842432 | NBN | 1 | 0.666666667 |
| BP | GO:0141005 | retrotransposon silencing by heterochromatin formation | 15/18888 | 0.024346888 | 0.103291122 | 0.06842432 | TDRD9 | 1 | 0.666666667 |
| BP | GO:1901741 | positive regulation of myoblast fusion | 15/18888 | 0.024346888 | 0.103291122 | 0.06842432 | MAPK14 | 1 | 0.666666667 |
| BP | GO:1902101 | positive regulation of metaphase/anaphase transition of cell cycle | 15/18888 | 0.024346888 | 0.103291122 | 0.06842432 | CDC20 | 1 | 0.666666667 |
| BP | GO:1903083 | protein localization to condensed chromosome | 15/18888 | 0.024346888 | 0.103291122 | 0.06842432 | CDK1 | 1 | 0.666666667 |
| BP | GO:0006119 | oxidative phosphorylation | 148/18888 | 0.024443763 | 0.103291122 | 0.06842432 | CDK1/CCNB1 | 2 | 0.135135135 |
| BP | GO:0033157 | regulation of intracellular protein transport | 149/18888 | 0.024751308 | 0.104129952 | 0.068979996 | CDK1/MAPK14 | 2 | 0.134228188 |
| BP | GO:0006266 | DNA ligation | 16/18888 | 0.025949455 | 0.105904828 | 0.070155747 | TOP2A | 1 | 0.625 |
| BP | GO:0009251 | glucan catabolic process | 16/18888 | 0.025949455 | 0.105904828 | 0.070155747 | PGM2 | 1 | 0.625 |
| BP | GO:0030033 | microvillus assembly | 16/18888 | 0.025949455 | 0.105904828 | 0.070155747 | KLF5 | 1 | 0.625 |
| BP | GO:0030214 | hyaluronan catabolic process | 16/18888 | 0.025949455 | 0.105904828 | 0.070155747 | HMMR | 1 | 0.625 |
| BP | GO:0032048 | cardiolipin metabolic process | 16/18888 | 0.025949455 | 0.105904828 | 0.070155747 | PGS1 | 1 | 0.625 |
| BP | GO:0090336 | positive regulation of brown fat cell differentiation | 16/18888 | 0.025949455 | 0.105904828 | 0.070155747 | MAPK14 | 1 | 0.625 |
| BP | GO:1904177 | regulation of adipose tissue development | 16/18888 | 0.025949455 | 0.105904828 | 0.070155747 | KLF7 | 1 | 0.625 |
| BP | GO:0031960 | response to corticosteroid | 156/18888 | 0.026948572 | 0.107109379 | 0.070953692 | ALPL/IL1RN | 2 | 0.128205128 |
| BP | GO:0001701 | in utero embryonic development | 401/18888 | 0.027509243 | 0.107109379 | 0.070953692 | CCNB1/NEK2/NBN | 3 | 0.074812968 |
| BP | GO:0000712 | resolution of meiotic recombination intermediates | 17/18888 | 0.027549475 | 0.107109379 | 0.070953692 | TOP2A | 1 | 0.588235294 |
| BP | GO:0010831 | positive regulation of myotube differentiation | 17/18888 | 0.027549475 | 0.107109379 | 0.070953692 | MAPK14 | 1 | 0.588235294 |
| BP | GO:0034035 | purine ribonucleoside bisphosphate metabolic process | 17/18888 | 0.027549475 | 0.107109379 | 0.070953692 | SULT1B1 | 1 | 0.588235294 |
| BP | GO:0036005 | response to macrophage colony-stimulating factor | 17/18888 | 0.027549475 | 0.107109379 | 0.070953692 | ALPL | 1 | 0.588235294 |
| BP | GO:0050427 | 3'-phosphoadenosine 5'-phosphosulfate metabolic process | 17/18888 | 0.027549475 | 0.107109379 | 0.070953692 | SULT1B1 | 1 | 0.588235294 |
| BP | GO:0051382 | kinetochore assembly | 17/18888 | 0.027549475 | 0.107109379 | 0.070953692 | CENPF | 1 | 0.588235294 |
| BP | GO:0072074 | kidney mesenchyme development | 17/18888 | 0.027549475 | 0.107109379 | 0.070953692 | BASP1 | 1 | 0.588235294 |
| BP | GO:1904355 | positive regulation of telomere capping | 17/18888 | 0.027549475 | 0.107109379 | 0.070953692 | NEK2 | 1 | 0.588235294 |
| BP | GO:0000723 | telomere maintenance | 158/18888 | 0.027590479 | 0.107109379 | 0.070953692 | NEK2/NBN | 2 | 0.126582278 |
| BP | GO:0016052 | carbohydrate catabolic process | 158/18888 | 0.027590479 | 0.107109379 | 0.070953692 | PGD/PGM2 | 2 | 0.126582278 |
| BP | GO:0006606 | protein import into nucleus | 161/18888 | 0.028564934 | 0.11044337 | 0.073162265 | CDK1/MAPK14 | 2 | 0.124223602 |
| BP | GO:0000272 | polysaccharide catabolic process | 18/18888 | 0.029146951 | 0.110743 | 0.073360753 | PGM2 | 1 | 0.555555556 |
| BP | GO:0060576 | intestinal epithelial cell development | 18/18888 | 0.029146951 | 0.110743 | 0.073360753 | KLF5 | 1 | 0.555555556 |
| BP | GO:1901970 | positive regulation of mitotic sister chromatid separation | 18/18888 | 0.029146951 | 0.110743 | 0.073360753 | CDC20 | 1 | 0.555555556 |
| BP | GO:1904666 | regulation of ubiquitin protein ligase activity | 18/18888 | 0.029146951 | 0.110743 | 0.073360753 | CDC20 | 1 | 0.555555556 |
| BP | GO:0007292 | female gamete generation | 163/18888 | 0.029222237 | 0.110743 | 0.073360753 | TOP2A/AURKA | 2 | 0.122699387 |
| BP | GO:0051170 | import into nucleus | 166/18888 | 0.030219577 | 0.111629284 | 0.073947864 | CDK1/MAPK14 | 2 | 0.120481928 |
| BP | GO:2000058 | regulation of ubiquitin-dependent protein catabolic process | 166/18888 | 0.030219577 | 0.111629284 | 0.073947864 | CDC20/AURKA | 2 | 0.120481928 |
| BP | GO:0000212 | meiotic spindle organization | 19/18888 | 0.030741887 | 0.111629284 | 0.073947864 | AURKA | 1 | 0.526315789 |
| BP | GO:0001502 | cartilage condensation | 19/18888 | 0.030741887 | 0.111629284 | 0.073947864 | MAPK14 | 1 | 0.526315789 |
| BP | GO:0009163 | nucleoside biosynthetic process | 19/18888 | 0.030741887 | 0.111629284 | 0.073947864 | PGM2 | 1 | 0.526315789 |
| BP | GO:0016202 | regulation of striated muscle tissue development | 19/18888 | 0.030741887 | 0.111629284 | 0.073947864 | CENPF | 1 | 0.526315789 |
| BP | GO:0034404 | nucleobase-containing small molecule biosynthetic process | 19/18888 | 0.030741887 | 0.111629284 | 0.073947864 | PGM2 | 1 | 0.526315789 |
| BP | GO:0045091 | regulation of single stranded viral RNA replication via double stranded DNA intermediate | 19/18888 | 0.030741887 | 0.111629284 | 0.073947864 | TOP2A | 1 | 0.526315789 |
| BP | GO:0060231 | mesenchymal to epithelial transition | 19/18888 | 0.030741887 | 0.111629284 | 0.073947864 | BASP1 | 1 | 0.526315789 |
| BP | GO:1902074 | response to salt | 19/18888 | 0.030741887 | 0.111629284 | 0.073947864 | ALPL | 1 | 0.526315789 |
| BP | GO:1903232 | melanosome assembly | 19/18888 | 0.030741887 | 0.111629284 | 0.073947864 | RAB32 | 1 | 0.526315789 |
| BP | GO:0007519 | skeletal muscle tissue development | 170/18888 | 0.031570389 | 0.113106427 | 0.074926385 | MAPK14/KLF5 | 2 | 0.117647059 |
| BP | GO:0072524 | pyridine-containing compound metabolic process | 170/18888 | 0.031570389 | 0.113106427 | 0.074926385 | ALPL/PGD | 2 | 0.117647059 |
| BP | GO:0007351 | tripartite regional subdivision | 20/18888 | 0.032334287 | 0.113106427 | 0.074926385 | BASP1 | 1 | 0.5 |
| BP | GO:0008595 | anterior/posterior axis specification embryo | 20/18888 | 0.032334287 | 0.113106427 | 0.074926385 | BASP1 | 1 | 0.5 |
| BP | GO:0010224 | response to UV-B | 20/18888 | 0.032334287 | 0.113106427 | 0.074926385 | MAPK14 | 1 | 0.5 |
| BP | GO:0034587 | piRNA processing | 20/18888 | 0.032334287 | 0.113106427 | 0.074926385 | TDRD9 | 1 | 0.5 |
| BP | GO:0039692 | single stranded viral RNA replication via double stranded DNA intermediate | 20/18888 | 0.032334287 | 0.113106427 | 0.074926385 | TOP2A | 1 | 0.5 |
| BP | GO:0055062 | phosphate ion homeostasis | 20/18888 | 0.032334287 | 0.113106427 | 0.074926385 | ALPL | 1 | 0.5 |
| BP | GO:0090128 | regulation of synapse maturation | 20/18888 | 0.032334287 | 0.113106427 | 0.074926385 | CDC20 | 1 | 0.5 |
| BP | GO:1901739 | regulation of myoblast fusion | 20/18888 | 0.032334287 | 0.113106427 | 0.074926385 | MAPK14 | 1 | 0.5 |
| BP | GO:0042692 | muscle cell differentiation | 428/18888 | 0.032489416 | 0.113106427 | 0.074926385 | CDK1/MAPK14/KLF5 | 3 | 0.070093458 |
| BP | GO:0022900 | electron transport chain | 175/18888 | 0.033292201 | 0.113106427 | 0.074926385 | CDK1/CCNB1 | 2 | 0.114285714 |
| BP | GO:1903008 | organelle disassembly | 175/18888 | 0.033292201 | 0.113106427 | 0.074926385 | CDK1/AURKA | 2 | 0.114285714 |
| BP | GO:0000729 | DNA double-strand break processing | 21/18888 | 0.033924155 | 0.113106427 | 0.074926385 | NBN | 1 | 0.476190476 |
| BP | GO:0006067 | ethanol metabolic process | 21/18888 | 0.033924155 | 0.113106427 | 0.074926385 | SULT1B1 | 1 | 0.476190476 |
| BP | GO:0006098 | pentose-phosphate shunt | 21/18888 | 0.033924155 | 0.113106427 | 0.074926385 | PGD | 1 | 0.476190476 |
| BP | GO:0032495 | response to muramyl dipeptide | 21/18888 | 0.033924155 | 0.113106427 | 0.074926385 | MAPK14 | 1 | 0.476190476 |
| BP | GO:0046128 | purine ribonucleoside metabolic process | 21/18888 | 0.033924155 | 0.113106427 | 0.074926385 | PGM2 | 1 | 0.476190476 |
| BP | GO:0051383 | kinetochore organization | 21/18888 | 0.033924155 | 0.113106427 | 0.074926385 | CENPF | 1 | 0.476190476 |
| BP | GO:0051443 | positive regulation of ubiquitin-protein transferase activity | 21/18888 | 0.033924155 | 0.113106427 | 0.074926385 | CDC20 | 1 | 0.476190476 |
| BP | GO:0090141 | positive regulation of mitochondrial fission | 21/18888 | 0.033924155 | 0.113106427 | 0.074926385 | AURKA | 1 | 0.476190476 |
| BP | GO:0090266 | regulation of mitotic cell cycle spindle assembly checkpoint | 21/18888 | 0.033924155 | 0.113106427 | 0.074926385 | CCNB1 | 1 | 0.476190476 |
| BP | GO:1903504 | regulation of mitotic spindle checkpoint | 21/18888 | 0.033924155 | 0.113106427 | 0.074926385 | CCNB1 | 1 | 0.476190476 |
| BP | GO:0072331 | signal transduction by p53 class mediator | 177/18888 | 0.033991146 | 0.113106427 | 0.074926385 | AURKA/NBN | 2 | 0.11299435 |
| BP | GO:0051223 | regulation of protein transport | 440/18888 | 0.034845775 | 0.115547621 | 0.076543533 | CDK1/MAPK14/KLF7 | 3 | 0.068181818 |
| BP | GO:0001832 | blastocyst growth | 22/18888 | 0.035511496 | 0.11574566 | 0.076674722 | NBN | 1 | 0.454545455 |
| BP | GO:0051923 | sulfation | 22/18888 | 0.035511496 | 0.11574566 | 0.076674722 | SULT1B1 | 1 | 0.454545455 |
| BP | GO:0061318 | renal filtration cell differentiation | 22/18888 | 0.035511496 | 0.11574566 | 0.076674722 | BASP1 | 1 | 0.454545455 |
| BP | GO:0072112 | podocyte differentiation | 22/18888 | 0.035511496 | 0.11574566 | 0.076674722 | BASP1 | 1 | 0.454545455 |
| BP | GO:0090231 | regulation of spindle checkpoint | 22/18888 | 0.035511496 | 0.11574566 | 0.076674722 | CCNB1 | 1 | 0.454545455 |
| BP | GO:0009410 | response to xenobiotic stimulus | 444/18888 | 0.035650685 | 0.115804095 | 0.076713432 | CDK1/SULT1B1/CENPF | 3 | 0.067567568 |
| BP | GO:0060143 | positive regulation of syncytium formation by plasma membrane fusion | 23/18888 | 0.037096312 | 0.119282754 | 0.07901784 | MAPK14 | 1 | 0.434782609 |
| BP | GO:0072311 | glomerular epithelial cell differentiation | 23/18888 | 0.037096312 | 0.119282754 | 0.07901784 | BASP1 | 1 | 0.434782609 |
| BP | GO:0098743 | cell aggregation | 23/18888 | 0.037096312 | 0.119282754 | 0.07901784 | MAPK14 | 1 | 0.434782609 |
| BP | GO:0000910 | cytokinesis | 188/18888 | 0.037937443 | 0.120712649 | 0.079965062 | AURKA/KIF4A | 2 | 0.106382979 |
| BP | GO:0032200 | telomere organization | 189/18888 | 0.038304617 | 0.120712649 | 0.079965062 | NEK2/NBN | 2 | 0.105820106 |
| BP | GO:0061136 | regulation of proteasomal protein catabolic process | 189/18888 | 0.038304617 | 0.120712649 | 0.079965062 | CDC20/AURKA | 2 | 0.105820106 |
| BP | GO:0006740 | NADPH regeneration | 24/18888 | 0.038678608 | 0.120712649 | 0.079965062 | PGD | 1 | 0.416666667 |
| BP | GO:0031145 | anaphase-promoting complex-dependent catabolic process | 24/18888 | 0.038678608 | 0.120712649 | 0.079965062 | CDC20 | 1 | 0.416666667 |
| BP | GO:0032528 | microvillus organization | 24/18888 | 0.038678608 | 0.120712649 | 0.079965062 | KLF5 | 1 | 0.416666667 |
| BP | GO:0034114 | regulation of heterotypic cell-cell adhesion | 24/18888 | 0.038678608 | 0.120712649 | 0.079965062 | IL1RN | 1 | 0.416666667 |
| BP | GO:0048634 | regulation of muscle organ development | 24/18888 | 0.038678608 | 0.120712649 | 0.079965062 | CENPF | 1 | 0.416666667 |
| BP | GO:1903429 | regulation of cell maturation | 24/18888 | 0.038678608 | 0.120712649 | 0.079965062 | AURKA | 1 | 0.416666667 |
| BP | GO:0006006 | glucose metabolic process | 192/18888 | 0.039414401 | 0.122210238 | 0.080957127 | MAPK14/PGM2 | 2 | 0.104166667 |
| BP | GO:0071897 | DNA biosynthetic process | 192/18888 | 0.039414401 | 0.122210238 | 0.080957127 | NEK2/CENPF | 2 | 0.104166667 |
| BP | GO:0007350 | blastoderm segmentation | 25/18888 | 0.040258388 | 0.1228331 | 0.081369736 | BASP1 | 1 | 0.4 |
| BP | GO:0031954 | positive regulation of protein autophosphorylation | 25/18888 | 0.040258388 | 0.1228331 | 0.081369736 | NBN | 1 | 0.4 |
| BP | GO:0035994 | response to muscle stretch | 25/18888 | 0.040258388 | 0.1228331 | 0.081369736 | MAPK14 | 1 | 0.4 |
| BP | GO:0090335 | regulation of brown fat cell differentiation | 25/18888 | 0.040258388 | 0.1228331 | 0.081369736 | MAPK14 | 1 | 0.4 |
| BP | GO:1901659 | glycosyl compound biosynthetic process | 25/18888 | 0.040258388 | 0.1228331 | 0.081369736 | PGM2 | 1 | 0.4 |
| BP | GO:0051656 | establishment of organelle localization | 472/18888 | 0.041555678 | 0.125638523 | 0.083228165 | CCNB1/NEK2/CENPF | 3 | 0.063559322 |
| BP | GO:0035330 | regulation of hippo signaling | 26/18888 | 0.041835655 | 0.125638523 | 0.083228165 | MAPK14 | 1 | 0.384615385 |
| BP | GO:0042278 | purine nucleoside metabolic process | 26/18888 | 0.041835655 | 0.125638523 | 0.083228165 | PGM2 | 1 | 0.384615385 |
| BP | GO:0070935 | 3'-UTR-mediated mRNA stabilization | 26/18888 | 0.041835655 | 0.125638523 | 0.083228165 | MAPK14 | 1 | 0.384615385 |
| BP | GO:0072010 | glomerular epithelium development | 26/18888 | 0.041835655 | 0.125638523 | 0.083228165 | BASP1 | 1 | 0.384615385 |
| BP | GO:0009060 | aerobic respiration | 199/18888 | 0.042051435 | 0.12589066 | 0.08339519 | CDK1/CCNB1 | 2 | 0.100502513 |
| BP | GO:0030073 | insulin secretion | 200/18888 | 0.042433512 | 0.126243002 | 0.083628596 | KLF7/IL1RN | 2 | 0.1 |
| BP | GO:0030705 | cytoskeleton-dependent intracellular transport | 200/18888 | 0.042433512 | 0.126243002 | 0.083628596 | MYO10/KIF4A | 2 | 0.1 |
| BP | GO:0007141 | male meiosis I | 27/18888 | 0.043410413 | 0.127953533 | 0.084761723 | TDRD9 | 1 | 0.37037037 |
| BP | GO:0046339 | diacylglycerol metabolic process | 27/18888 | 0.043410413 | 0.127953533 | 0.084761723 | PGS1 | 1 | 0.37037037 |
| BP | GO:0099560 | synaptic membrane adhesion | 27/18888 | 0.043410413 | 0.127953533 | 0.084761723 | MAPK14 | 1 | 0.37037037 |
| BP | GO:0022412 | cellular process involved in reproduction in multicellular organism | 482/18888 | 0.04377852 | 0.128641498 | 0.085217459 | TDRD9/TOP2A/AURKA | 3 | 0.062240664 |
| BP | GO:0120032 | regulation of plasma membrane bounded cell projection assembly | 204/18888 | 0.043975008 | 0.128822494 | 0.085337359 | MYO10/KLF5 | 2 | 0.098039216 |
| BP | GO:0007623 | circadian rhythm | 205/18888 | 0.044363655 | 0.129004345 | 0.085457825 | CDK1/TOP2A | 2 | 0.097560976 |
| BP | GO:0045732 | positive regulation of protein catabolic process | 205/18888 | 0.044363655 | 0.129004345 | 0.085457825 | CDC20/AURKA | 2 | 0.097560976 |
| BP | GO:0016051 | carbohydrate biosynthetic process | 206/18888 | 0.0447536 | 0.129004345 | 0.085457825 | PGD/PGM2 | 2 | 0.097087379 |
| BP | GO:0060491 | regulation of cell projection assembly | 206/18888 | 0.0447536 | 0.129004345 | 0.085457825 | MYO10/KLF5 | 2 | 0.097087379 |
| BP | GO:0006027 | glycosaminoglycan catabolic process | 28/18888 | 0.044982667 | 0.129004345 | 0.085457825 | HMMR | 1 | 0.357142857 |
| BP | GO:0034508 | centromere complex assembly | 28/18888 | 0.044982667 | 0.129004345 | 0.085457825 | CENPF | 1 | 0.357142857 |
| BP | GO:0060575 | intestinal epithelial cell differentiation | 28/18888 | 0.044982667 | 0.129004345 | 0.085457825 | KLF5 | 1 | 0.357142857 |
| BP | GO:0009566 | fertilization | 207/18888 | 0.04514484 | 0.129081802 | 0.085509136 | TDRD9/CDK1 | 2 | 0.096618357 |
| BP | GO:0031667 | response to nutrient levels | 490/18888 | 0.045599558 | 0.129992769 | 0.086112598 | ALPL/MAPK14/RRAGD | 3 | 0.06122449 |
| BP | GO:0050808 | synapse organization | 493/18888 | 0.0462922 | 0.130757532 | 0.086619208 | LRRN1/MAPK14/CDC20 | 3 | 0.060851927 |
| BP | GO:0046474 | glycerophospholipid biosynthetic process | 210/18888 | 0.046326272 | 0.130757532 | 0.086619208 | PGS1/PIGK | 2 | 0.095238095 |
| BP | GO:0042403 | thyroid hormone metabolic process | 29/18888 | 0.04655242 | 0.130757532 | 0.086619208 | SULT1B1 | 1 | 0.344827586 |
| BP | GO:0060142 | regulation of syncytium formation by plasma membrane fusion | 29/18888 | 0.04655242 | 0.130757532 | 0.086619208 | MAPK14 | 1 | 0.344827586 |
| BP | GO:1905820 | positive regulation of chromosome separation | 29/18888 | 0.04655242 | 0.130757532 | 0.086619208 | CDC20 | 1 | 0.344827586 |
| BP | GO:0022407 | regulation of cell-cell adhesion | 496/18888 | 0.046990144 | 0.131599962 | 0.087177269 | MYO10/MAPK14/IL1RN | 3 | 0.060483871 |
| BP | GO:2000241 | regulation of reproductive process | 214/18888 | 0.047919327 | 0.132433113 | 0.087729182 | CDC20/AURKA | 2 | 0.093457944 |
| BP | GO:0039694 | viral RNA genome replication | 30/18888 | 0.048119676 | 0.132433113 | 0.087729182 | TOP2A | 1 | 0.333333333 |
| BP | GO:0051156 | glucose 6-phosphate metabolic process | 30/18888 | 0.048119676 | 0.132433113 | 0.087729182 | PGD | 1 | 0.333333333 |
| BP | GO:0090140 | regulation of mitochondrial fission | 30/18888 | 0.048119676 | 0.132433113 | 0.087729182 | AURKA | 1 | 0.333333333 |
| BP | GO:0090382 | phagosome maturation | 30/18888 | 0.048119676 | 0.132433113 | 0.087729182 | RAB32 | 1 | 0.333333333 |
| BP | GO:0097421 | liver regeneration | 30/18888 | 0.048119676 | 0.132433113 | 0.087729182 | AURKA | 1 | 0.333333333 |
| BP | GO:0060348 | bone development | 215/18888 | 0.048320739 | 0.132604328 | 0.087842603 | ALPL/MAPK14 | 2 | 0.093023256 |
| BP | GO:0045637 | regulation of myeloid cell differentiation | 218/18888 | 0.049532453 | 0.134415406 | 0.089042336 | MAPK14/HMGB3 | 2 | 0.091743119 |
| BP | GO:0003338 | metanephros morphogenesis | 31/18888 | 0.049684438 | 0.134415406 | 0.089042336 | BASP1 | 1 | 0.322580645 |
| BP | GO:0007064 | mitotic sister chromatid cohesion | 31/18888 | 0.049684438 | 0.134415406 | 0.089042336 | CDC20 | 1 | 0.322580645 |
| BP | GO:0030262 | apoptotic nuclear changes | 31/18888 | 0.049684438 | 0.134415406 | 0.089042336 | TOP2A | 1 | 0.322580645 |
| BP | GO:0045070 | positive regulation of viral genome replication | 31/18888 | 0.049684438 | 0.134415406 | 0.089042336 | TOP2A | 1 | 0.322580645 |
| BP | GO:0009952 | anterior/posterior pattern specification | 219/18888 | 0.049938833 | 0.134721994 | 0.089245432 | AURKA/BASP1 | 2 | 0.091324201 |
| CC | GO:0005819 | spindle | 433/19894 | 1.82E-08 | 2.31E-06 | 1.57E-06 | HMMR/CDK1/CCNB1/MAPK14/CDC20/AURKA/NEK2/CENPF/KIF4A | 9 | 0.207852194 |
| CC | GO:0098687 | chromosomal region | 402/19894 | 1.78E-07 | 1.08E-05 | 7.35E-06 | CDK1/CCNB1/TOP2A/CDC20/AURKA/NEK2/CENPF/NBN | 8 | 0.199004975 |
| CC | GO:0000793 | condensed chromosome | 281/19894 | 2.59E-07 | 1.08E-05 | 7.35E-06 | CCNB1/MKI67/TOP2A/CDC20/AURKA/NEK2/CENPF | 7 | 0.24911032 |
| CC | GO:0000922 | spindle pole | 177/19894 | 3.41E-07 | 1.08E-05 | 7.35E-06 | CCNB1/MAPK14/CDC20/AURKA/NEK2/CENPF | 6 | 0.338983051 |
| CC | GO:0000775 | chromosome centromeric region | 257/19894 | 3.00E-06 | 7.61E-05 | 5.17E-05 | CCNB1/TOP2A/CDC20/AURKA/NEK2/CENPF | 6 | 0.233463035 |
| CC | GO:0000776 | kinetochore | 170/19894 | 7.18E-06 | 0.0001519 | 0.000103239 | CCNB1/CDC20/AURKA/NEK2/CENPF | 5 | 0.294117647 |
| CC | GO:0000779 | condensed chromosome centromeric region | 181/19894 | 9.73E-06 | 0.000176572 | 0.000120008 | CCNB1/CDC20/AURKA/NEK2/CENPF | 5 | 0.276243094 |
| CC | GO:0030496 | midbody | 205/19894 | 1.78E-05 | 0.00028206 | 0.000191703 | CDK1/AURKA/NEK2/CENPF/KIF4A | 5 | 0.243902439 |
| CC | GO:0045120 | pronucleus | 14/19894 | 0.000225367 | 0.003180182 | 0.002161417 | AURKA/CENPF | 2 | 1.428571429 |
| CC | GO:0005876 | spindle microtubule | 82/19894 | 0.000307102 | 0.003900194 | 0.002650774 | CDK1/AURKA/KIF4A | 3 | 0.365853659 |
| CC | GO:0000940 | outer kinetochore | 20/19894 | 0.000467719 | 0.005400029 | 0.00367014 | CCNB1/CENPF | 2 | 1 |
| CC | GO:0016363 | nuclear matrix | 128/19894 | 0.001125894 | 0.011915707 | 0.008098533 | CENPF/BASP1/KIF4A | 3 | 0.234375 |
| CC | GO:0034399 | nuclear periphery | 150/19894 | 0.001775205 | 0.017342385 | 0.011786784 | CENPF/BASP1/KIF4A | 3 | 0.2 |
| CC | GO:0000307 | cyclin-dependent protein kinase holoenzyme complex | 56/19894 | 0.003656575 | 0.033170361 | 0.022544298 | CDK1/CCNB1 | 2 | 0.357142857 |
| CC | GO:0043073 | germ cell nucleus | 66/19894 | 0.005042376 | 0.042692117 | 0.029015778 | TOP2A/AURKA | 2 | 0.303030303 |
| CC | GO:0005874 | microtubule | 468/19894 | 0.006453766 | 0.051226764 | 0.034816367 | CDK1/AURKA/NEK2/KIF4A | 4 | 0.085470085 |
| CC | GO:0016605 | PML body | 108/19894 | 0.013024963 | 0.096598052 | 0.065653048 | BASP1/NBN | 2 | 0.185185185 |
| CC | GO:0034774 | secretory granule lumen | 322/19894 | 0.014729465 | 0.096598052 | 0.065653048 | S100P/MAPK14/PGM2 | 3 | 0.093167702 |
| CC | GO:0060205 | cytoplasmic vesicle lumen | 325/19894 | 0.015097279 | 0.096598052 | 0.065653048 | S100P/MAPK14/PGM2 | 3 | 0.092307692 |
| CC | GO:0031983 | vesicle lumen | 326/19894 | 0.015221042 | 0.096598052 | 0.065653048 | S100P/MAPK14/PGM2 | 3 | 0.09202454 |
| CC | GO:0008303 | caspase complex | 10/19894 | 0.015972906 | 0.096598052 | 0.065653048 | PIGK | 1 | 1 |
| CC | GO:1904813 | ficolin-1-rich granule lumen | 124/19894 | 0.01691912 | 0.097669468 | 0.066381238 | MAPK14/PGM2 | 2 | 0.161290323 |
| CC | GO:0042405 | nuclear inclusion body | 12/19894 | 0.019137694 | 0.105673354 | 0.071821094 | NBN | 1 | 0.833333333 |
| CC | GO:1902554 | serine/threonine protein kinase complex | 136/19894 | 0.020125285 | 0.106496298 | 0.07238041 | CDK1/CCNB1 | 2 | 0.147058824 |
| CC | GO:0031616 | spindle pole centrosome | 14/19894 | 0.02229262 | 0.107477324 | 0.073047166 | AURKA | 1 | 0.714285714 |
| CC | GO:0001674 | female germ cell nucleus | 15/19894 | 0.023866395 | 0.107477324 | 0.073047166 | AURKA | 1 | 0.666666667 |
| CC | GO:0072687 | meiotic spindle | 15/19894 | 0.023866395 | 0.107477324 | 0.073047166 | AURKA | 1 | 0.666666667 |
| CC | GO:1902911 | protein kinase complex | 150/19894 | 0.024160679 | 0.107477324 | 0.073047166 | CDK1/CCNB1 | 2 | 0.133333333 |
| CC | GO:0005875 | microtubule associated complex | 157/19894 | 0.026292993 | 0.107477324 | 0.073047166 | AURKA/KIF4A | 2 | 0.127388535 |
| CC | GO:0042599 | lamellar body | 17/19894 | 0.027006585 | 0.107477324 | 0.073047166 | CKAP4 | 1 | 0.588235294 |
| CC | GO:0005814 | centriole | 160/19894 | 0.027229584 | 0.107477324 | 0.073047166 | TOP2A/AURKA | 2 | 0.125 |
| CC | GO:0031527 | filopodium membrane | 18/19894 | 0.028573008 | 0.107477324 | 0.073047166 | MYO10 | 1 | 0.555555556 |
| CC | GO:0032433 | filopodium tip | 19/19894 | 0.030136988 | 0.107477324 | 0.073047166 | MYO10 | 1 | 0.526315789 |
| CC | GO:0000781 | telomeric region | 173/19894 | 0.031441601 | 0.107477324 | 0.073047166 | CDK1/NBN | 2 | 0.115606936 |
| CC | GO:0036064 | ciliary basal body | 173/19894 | 0.031441601 | 0.107477324 | 0.073047166 | AURKA/CENPF | 2 | 0.115606936 |
| CC | GO:0005680 | anaphase-promoting complex | 22/19894 | 0.034814305 | 0.107477324 | 0.073047166 | CDC20 | 1 | 0.454545455 |
| CC | GO:0033162 | melanosome membrane | 22/19894 | 0.034814305 | 0.107477324 | 0.073047166 | RAB32 | 1 | 0.454545455 |
| CC | GO:0044233 | mitochondria-associated endoplasmic reticulum membrane | 22/19894 | 0.034814305 | 0.107477324 | 0.073047166 | RAB32 | 1 | 0.454545455 |
| CC | GO:0045009 | chitosome | 22/19894 | 0.034814305 | 0.107477324 | 0.073047166 | RAB32 | 1 | 0.454545455 |
| CC | GO:0090741 | pigment granule membrane | 22/19894 | 0.034814305 | 0.107477324 | 0.073047166 | RAB32 | 1 | 0.454545455 |
| CC | GO:0072686 | mitotic spindle | 184/19894 | 0.035194218 | 0.107477324 | 0.073047166 | CDK1/AURKA | 2 | 0.108695652 |
| CC | GO:0101002 | ficolin-1-rich granule | 185/19894 | 0.035543682 | 0.107477324 | 0.073047166 | MAPK14/PGM2 | 2 | 0.108108108 |
| CC | GO:0043186 | P granule | 27/19894 | 0.042561316 | 0.120117492 | 0.08163808 | TDRD9 | 1 | 0.37037037 |
| CC | GO:0045495 | pole plasm | 27/19894 | 0.042561316 | 0.120117492 | 0.08163808 | TDRD9 | 1 | 0.37037037 |
| CC | GO:0060293 | germ plasm | 27/19894 | 0.042561316 | 0.120117492 | 0.08163808 | TDRD9 | 1 | 0.37037037 |
| MF | GO:0008301 | DNA binding bending | 18/18522 | 0.000434859 | 0.041124702 | 0.027855633 | TOP2A/HMGB3 | 2 | 1.111111111 |
| MF | GO:0035173 | histone kinase activity | 23/18522 | 0.000715212 | 0.041124702 | 0.027855633 | CDK1/AURKA | 2 | 0.869565217 |
| MF | GO:0004712 | protein serine/threonine/tyrosine kinase activity | 43/18522 | 0.002498299 | 0.076395126 | 0.051745898 | MAPK14/AURKA | 2 | 0.465116279 |
| MF | GO:0016853 | isomerase activity | 161/18522 | 0.002657222 | 0.076395126 | 0.051745898 | TOP2A/PIGK/PGM2 | 3 | 0.186335404 |
| MF | GO:0106310 | protein serine kinase activity | 363/18522 | 0.003382165 | 0.077789791 | 0.052690568 | CDK1/MAPK14/AURKA/NEK2 | 4 | 0.110192837 |
| MF | GO:0004674 | protein serine/threonine kinase activity | 427/18522 | 0.006009636 | 0.113910663 | 0.077156879 | CDK1/MAPK14/AURKA/NEK2 | 4 | 0.093676815 |
| MF | GO:0000287 | magnesium ion binding | 227/18522 | 0.006933693 | 0.113910663 | 0.077156879 | S100P/TOP2A/PGM2 | 3 | 0.13215859 |
| MF | GO:0140297 | DNA-binding transcription factor binding | 478/18522 | 0.008888313 | 0.127769504 | 0.086544103 | MAPK14/KLF5/CENPF/NBN | 4 | 0.083682008 |
| MF | GO:0003774 | cytoskeletal motor activity | 112/18522 | 0.015970707 | 0.138882536 | 0.094071466 | MYO10/KIF4A | 2 | 0.178571429 |
| MF | GO:0008353 | RNA polymerase II CTD heptapeptide repeat kinase activity | 10/18522 | 0.017147184 | 0.138882536 | 0.094071466 | CDK1 | 1 | 1 |
| MF | GO:0016868 | intramolecular phosphotransferase activity | 10/18522 | 0.017147184 | 0.138882536 | 0.094071466 | PGM2 | 1 | 1 |
| MF | GO:0140996 | histone H3 kinase activity | 11/18522 | 0.018846152 | 0.138882536 | 0.094071466 | AURKA | 1 | 0.909090909 |
| MF | GO:0035497 | cAMP response element binding | 12/18522 | 0.020542274 | 0.138882536 | 0.094071466 | CREB5 | 1 | 0.833333333 |
| MF | GO:1990247 | N6-methyladenosine-containing RNA reader activity | 12/18522 | 0.020542274 | 0.138882536 | 0.094071466 | IGF2BP3 | 1 | 0.833333333 |
| MF | GO:0046982 | protein heterodimerization activity | 353/18522 | 0.022631201 | 0.138882536 | 0.094071466 | TOP2A/AURKA/RRAGD | 3 | 0.084985836 |
| MF | GO:0019903 | protein phosphatase binding | 142/18522 | 0.024910624 | 0.138882536 | 0.094071466 | MAPK14/NEK2 | 2 | 0.14084507 |
| MF | GO:0097027 | ubiquitin-protein transferase activator activity | 15/18522 | 0.025613617 | 0.138882536 | 0.094071466 | CDC20 | 1 | 0.666666667 |
| MF | GO:0004707 | MAP kinase activity | 16/18522 | 0.027298404 | 0.138882536 | 0.094071466 | MAPK14 | 1 | 0.625 |
| MF | GO:0004708 | MAP kinase kinase activity | 16/18522 | 0.027298404 | 0.138882536 | 0.094071466 | MAPK14 | 1 | 0.625 |
| MF | GO:0061575 | cyclin-dependent protein serine/threonine kinase activator activity | 16/18522 | 0.027298404 | 0.138882536 | 0.094071466 | CCNB1 | 1 | 0.625 |
| MF | GO:0005149 | interleukin-1 receptor binding | 17/18522 | 0.02898037 | 0.138882536 | 0.094071466 | IL1RN | 1 | 0.588235294 |
| MF | GO:0017110 | nucleoside diphosphate phosphatase activity | 17/18522 | 0.02898037 | 0.138882536 | 0.094071466 | ALPL | 1 | 0.588235294 |
| MF | GO:0000400 | four-way junction DNA binding | 18/18522 | 0.030659517 | 0.138882536 | 0.094071466 | HMGB3 | 1 | 0.555555556 |
| MF | GO:0003756 | protein disulfide isomerase activity | 18/18522 | 0.030659517 | 0.138882536 | 0.094071466 | PIGK | 1 | 0.555555556 |
| MF | GO:0016864 | intramolecular oxidoreductase activity transposing S-S bonds | 18/18522 | 0.030659517 | 0.138882536 | 0.094071466 | PIGK | 1 | 0.555555556 |
| MF | GO:0140517 | protein-RNA adaptor activity | 20/18522 | 0.034009378 | 0.138882536 | 0.094071466 | IGF2BP3 | 1 | 0.5 |
| MF | GO:0030742 | GTP-dependent protein binding | 21/18522 | 0.0356801 | 0.138882536 | 0.094071466 | RAB32 | 1 | 0.476190476 |
| MF | GO:0016780 | phosphotransferase activity for other substituted phosphate groups | 22/18522 | 0.037348023 | 0.138882536 | 0.094071466 | PGS1 | 1 | 0.454545455 |
| MF | GO:0140994 | RNA polymerase II CTD heptapeptide repeat modifying activity | 23/18522 | 0.03901315 | 0.138882536 | 0.094071466 | CDK1 | 1 | 0.434782609 |
| MF | GO:0005540 | hyaluronic acid binding | 24/18522 | 0.040675488 | 0.138882536 | 0.094071466 | HMMR | 1 | 0.416666667 |
| MF | GO:0048027 | mRNA 5'-UTR binding | 24/18522 | 0.040675488 | 0.138882536 | 0.094071466 | IGF2BP3 | 1 | 0.416666667 |
| MF | GO:0019902 | phosphatase binding | 189/18522 | 0.042053057 | 0.138882536 | 0.094071466 | MAPK14/NEK2 | 2 | 0.105820106 |
| MF | GO:0070840 | dynein complex binding | 25/18522 | 0.042335039 | 0.138882536 | 0.094071466 | CENPF | 1 | 0.4 |
| MF | GO:0030507 | spectrin binding | 26/18522 | 0.043991809 | 0.138882536 | 0.094071466 | MYO10 | 1 | 0.384615385 |
| MF | GO:0016866 | intramolecular transferase activity | 28/18522 | 0.047297024 | 0.138882536 | 0.094071466 | PGM2 | 1 | 0.357142857 |
| MF | GO:0051019 | mitogen-activated protein kinase binding | 28/18522 | 0.047297024 | 0.138882536 | 0.094071466 | MAPK14 | 1 | 0.357142857 |
| MF | GO:0004693 | cyclin-dependent protein serine/threonine kinase activity | 29/18522 | 0.048945477 | 0.138882536 | 0.094071466 | CDK1 | 1 | 0.344827586 |
| MF | GO:0051861 | glycolipid binding | 29/18522 | 0.048945477 | 0.138882536 | 0.094071466 | PIGK | 1 | 0.344827586 |
| MF | GO:0055106 | ubiquitin-protein transferase regulator activity | 29/18522 | 0.048945477 | 0.138882536 | 0.094071466 | CDC20 | 1 | 0.344827586 |
| MF | GO:0097472 | cyclin-dependent protein kinase activity | 29/18522 | 0.048945477 | 0.138882536 | 0.094071466 | CDK1 | 1 | 0.344827586 |
| MF | GO:0140993 | histone modifying activity | 207/18522 | 0.049514643 | 0.138882536 | 0.094071466 | CDK1/AURKA | 2 | 0.096618357 |

Listed are enriched GO-BP terms with their GO ID, term name, gene ratio, adjusted *P*-value, and core enrichment genes.

**Supplementary Table S3. KEGG pathway enrichment analysis of candidate genes.**

|  | category | subcategory | ID | Description | BgRatio | pvalue | p.adjust | qvalue | geneID | Count | richFactor |  |
| --- | --- | --- | --- | --- | --- | --- | --- | --- | --- | --- | --- | --- |
| 1 | Cellular Processes | Cell growth and death | hsa04114 | Oocyte meiosis | 139/8846 | 1.14298923036232e-05 | 0.00131443761491666 | 0.00120314655827612 | CDK1/CCNB1/MAPK14/CDC20/AURKA | 5 | 0.359712230215827 |  |
| 2 | Organismal Systems | Endocrine system | hsa04914 | Progesterone-mediated oocyte maturation | 111/8846 | 9.74383561666018e-05 | 0.0056027054795796 | 0.00512833453508431 | CDK1/CCNB1/MAPK14/AURKA | 4 | 0.36036036036036 |  |
| 3 | Cellular Processes | Cell growth and death | hsa04218 | Cellular senescence | 157/8846 | 0.000370663863866688 | 0.0142087814482231 | 0.0130057496093575 | CDK1/CCNB1/MAPK14/NBN | 4 | 0.254777070063694 |  |
| 5 | Cellular Processes | Cell growth and death | hsa04110 | Cell cycle | 158/8846 | 0.00509837517073494 | 0.117262628926904 | 0.107334214120736 | CDK1/CCNB1/CDC20 | 3 | 0.189873417721519 |  |
| 6 | Human Diseases | Cancer: overview | hsa05203 | Viral carcinogenesis | 205/8846 | 0.0104529184861105 | 0.175508889685856 | 0.160648869277671 | CDK1/CREB5/CDC20 | 3 | 0.146341463414634 |  |
| 7 | Human Diseases | Infectious disease: viral | hsa05170 | Human immunodeficiency virus 1 infection | 213/8846 | 0.0115972427523803 | 0.175508889685856 | 0.160648869277671 | CDK1/CCNB1/MAPK14 | 3 | 0.140845070422535 |  |
| 4 | Metabolism | Carbohydrate metabolism | hsa00030 | Pentose phosphate pathway | 31/8846 | 0.00217126006140394 | 0.0624237267653634 | 0.0571384226685249 | PGD/PGM2 | 2 | 0.645161290322581 |  |
| 8 | Cellular Processes | Cell growth and death | hsa04115 | p53 signaling pathway | 75/8846 | 0.012209314065103 | 0.175508889685856 | 0.160648869277671 | CDK1/CCNB1 | 2 | 0.266666666666667 |  |
| 9 | Environmental Information Processing | Signal transduction | hsa04668 | TNF signaling pathway | 119/8846 | 0.0291140167524066 | 0.29343115384411 | 0.268586868507194 | CREB5/MAPK14 | 2 | 0.168067226890756 |  |
| 10 | Organismal Systems | Endocrine system | hsa04935 | Growth hormone synthesis, secretion and action | 122/8846 | 0.0304837466886859 | 0.29343115384411 | 0.268586868507194 | CREB5/MAPK14 | 2 | 0.163934426229508 |  |
| 12 | Organismal Systems | Endocrine system | hsa04926 | Relaxin signaling pathway | 130/8846 | 0.0342603679928361 | 0.29343115384411 | 0.268586868507194 | CREB5/MAPK14 | 2 | 0.153846153846154 |  |
| 13 | Organismal Systems | Nervous system | hsa04728 | Dopaminergic synapse | 132/8846 | 0.0352321153299011 | 0.29343115384411 | 0.268586868507194 | CREB5/MAPK14 | 2 | 0.151515151515152 |  |
| 14 | Environmental Information Processing | Signal transduction | hsa04068 | FoxO signaling pathway | 133/8846 | 0.0357220535114568 | 0.29343115384411 | 0.268586868507194 | CCNB1/MAPK14 | 2 | 0.150375939849624 |  |
| 15 | Organismal Systems | Circulatory system | hsa04261 | Adrenergic signaling in cardiomyocytes | 154/8846 | 0.0466123378391049 | 0.357361256766471 | 0.327104125186701 | CREB5/MAPK14 | 2 | 0.12987012987013 |  |
| 11 | Metabolism | Metabolism of cofactors and vitamins | hsa00730 | Thiamine metabolism | 15/8846 | 0.0334081543551299 | 0.29343115384411 | 0.268586868507194 | ALPL | 1 | 0.666666666666667 |  |

Pathways were identified using the KEGG database. Listed are the top enriched pathways with adjusted *P* < 0.05.
